# Elucidation of Structure–Activity Relationships in Indolobenzazepine-Derived Ligands and Their Copper(II) Complexes: the Role of Key Structural Components and Insight into the Mechanism of Action

**DOI:** 10.1021/acs.inorgchem.2c01375

**Published:** 2022-06-17

**Authors:** Irina Kuznetcova, Felix Bacher, Samah Mutasim Alfadul, Max Jing Rui Tham, Wee Han Ang, Maria V. Babak, Peter Rapta, Vladimir B. Arion

**Affiliations:** † Institute of Inorganic Chemistry of the University of Vienna, Währinger Strasse 42, A-1090 Vienna, Austria; ‡ Drug Discovery Lab, Department of Chemistry, City University of Hong Kong, Kowloon, Hong Kong SAR 999077, China; § Department of Chemistry, National University of Singapore, 4 Science Drive 2, Singapore 117544, Singapore; ∥ Institute of Physical Chemistry and Chemical Physics, Faculty of Chemical and Food Technology, Slovak University of Technology in Bratislava, Radlinského 9, SK-81237 Bratislava, Slovak Republic

## Abstract

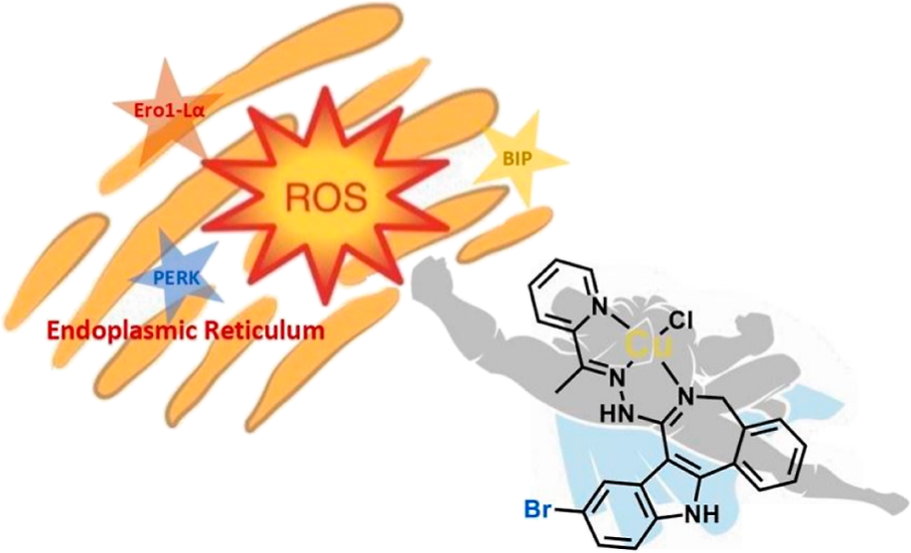

Indolo[3,2-*d*][1]benzazepines (paullones), indolo[3,2-*d*][2]benzazepines, and indolo[2,3-*d*][2]benzazepines (latonduines) are isomeric scaffolds of current medicinal interest. Herein, we prepared a small library of novel indolo[3,2-*d*][2]benzazepine-derived ligands **HL**^**1**^–**HL**^**4**^ and copper(II) complexes **1**–**4**. All compounds were characterized by spectroscopic methods (^1^H and ^13^C NMR, UV–vis, IR) and electrospray ionization (ESI) mass spectrometry, while complexes **2** and **3**, in addition, by X-ray crystallography. Their purity was confirmed by HPLC coupled with high-resolution ESI mass spectrometry and/or elemental analysis. The stability of compounds in aqueous solutions in the presence of DMSO was confirmed by ^1^H NMR and UV–vis spectroscopy measurements. The compounds revealed high antiproliferative activity in vitro in the breast cancer cell line MDA-MB-231 and hepatocellular carcinoma cell line LM3 in the low micromolar to nanomolar concentration range. Important structure–activity relationships were deduced from the comparison of anticancer activities of **HL**^**1**^–**HL**^**4**^ and **1**–**4** with those of structurally similar paullone-derived (**HL**^**5**^–**HL**^**7**^ and **5**–**7**) and latonduine-derived scaffolds (**HL**^**8**^–**HL**^**11**^ and **8**–**11**). The high anticancer activity of the lead drug candidate **4** was linked to reactive oxygen species and endoplasmic reticulum stress induction, which were confirmed by fluorescent microscopy and Western blot analysis.

## Abstract 2

Highly antiproliferative copper(II) complexes with Schiff bases derived from indolo[3,2-*d*]benzazepines and 2-formyl- and 2-acetylpyridine have been synthesized and characterized. Western blot analysis of endoplasmic reticulum stress biomarkers PERK, BIP, and Ero-1-Lα after treatment of breast cancer cells MDA-MB-231 with increasing concentrations of lead drug candidate **4** revealed ER stress activation and production of toxic ROS, which finally led to cell death.

## Introduction

The search for effective metal-based anticancer drugs with new mechanisms of action to reduce side effects and overcome acquired drug resistance continues to attract the attention of researchers.^1−12^ Various mechanisms of drug resistance in cancer cells were shown to be intertwined with their ability to adapt to the proteotoxic stress.^13^ Therefore, one promising direction is the development of anticancer drug candidates inducing severe endoplasmic reticulum (ER) stress, resulting in the inability of cancer cells to restore protein homeostasis and cancer cell death.^14−16^ ER is the largest organelle in eukaryotic cells for calcium storage, lipid biosynthesis, entry, folding, and assembly of proteins for a secretory pathway.^17^ If one of the three main ER functions is disturbed, ER stress is induced. This stress activates an adaptive mechanism in cells to restore the ER proteostasis known as unfolded protein response (UPR). There are three major UPR pathways to restore protein homeostasis in cells: PKR-like ER kinase (PERK), inositol requiring 1 (IRE1), and activating transcription factor 6 (ATF6).^18^ If the ability of a cell to remedy stress is disrupted, this leads to cell death.^19^

ER induction was reported as the main mechanism of cell death for small organic molecules including naturally occurring anticancer drugs, such as thapsigargin.^20^ Similarly, various metal complexes, including Pt, Ru, Au, Os, were shown to induce ER stress,^19,21,22^ leading to cancer cell death and induction of immunogenic response.^23−25^ Metal complexes offer a number of advantages over classic organic molecules since their structure can be easily fine-tuned to ensure the desired mechanism of action, including activation of ER stress. For example, it was shown that replacement of labile chlorido ligands in the cyclometalated Pt(II) complexes with nonleaving groups enabled the switch of the mechanism of action from DNA binding to ER stress.^26^ Similarly, variations in the π-acidity of the Schiff bases in Ru(II)-arene complexes governed the underlying mechanism of ER stress induction.^15^

Recently, we^27,28^ and other researchers^21,22^ demonstrated that various copper(II) complexes could also disrupt cancer cell function via induction of ER perturbations. Tetrahedrally distorted and square-planar copper(II) complexes with bidentate *N*,*O*-Schiff bases derived from substituted salicylaldehydes and 2-(2-fluorophenyl)-3-aminoquinoline showed remarkable cytotoxicity in a number of cancer cell lines in vitro and in vivo. This is due to their ability to generate reactive oxygen species (ROS) inducing mitochondrial disruption, caspase cascade activation, and ER stress, leading to sub-G1 cell cycle arrest and apoptosis.^29^ Five-coordinate copper(II)-bis(phenanthroline) complexes containing substituted imidazolidine-2-thione ligands induced ER stress and UPR in human ovarian A2780 cancer cells as confirmed by morphological TEM studies and Western blotting.^30^

Overall, the most common features of metal-based drugs as ER-stress inducers have been specified as (i) positively charged species with (ii) high lipophilicity and (iii) molecular weight > 500 g/mol.^14^

We hypothesized that copper(II) complexes with indolobenzazepine-derived ligands might effectively induce ER stress in cancer cells. Previously, we showed that indolobenzazepines and structurally similar compounds formed monocationic complexes of 1:1 or 1:2 metal-to-ligand stoichiometry at the physiological pH with the molecular weight close to or even higher than 500 g/mol.^31−34^ The hydrophobic nature of the ligands implies that their metal complexes could be readily taken up by the cells and localize in lipid dense ER. This suggestion is further supported by our recent findings established by fluorescence microscopy that an indolo[3,2-*c*]quinoline-derived ligand ^EtOOC^HL^COOEt^ with inherent fluorescence properties and dizinc(II) complex [Zn_2_(^MeOOC^L^COO^)(OAc)_2_] were taken by SW480 cells and accumulated in the ER and lysosomes.^35^

**Chart 1:**
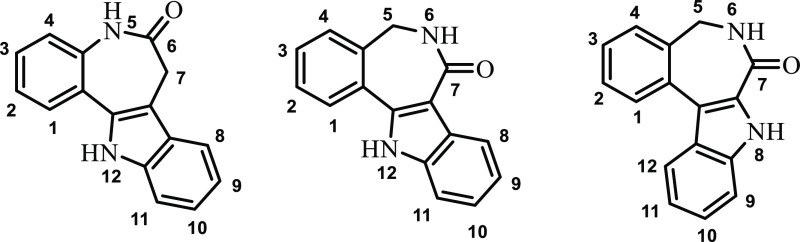
Indolo[3,2-*d*][1]benzazepine (Paullone) (Left), Indolo[3,2-*d*][2]benzazepine (Middle), and Indolo[2,3-*d*][2]benzazepine (Latonduine) Backbone (Right).

It should be noted that indolo[3,2-*d*][2]benzazepine scaffolds are isomeric to indolo[3,2-*d*][1]benzazepines (paullones) and indolo[2,3-*d*][2]benzazepines (latonduines) (Chart 1). Both paullone- and latonduine-derived ligands and their copper(II) complexes demonstrated high antiproliferative activity in various cell lines.^33,36^

**Scheme 1:**
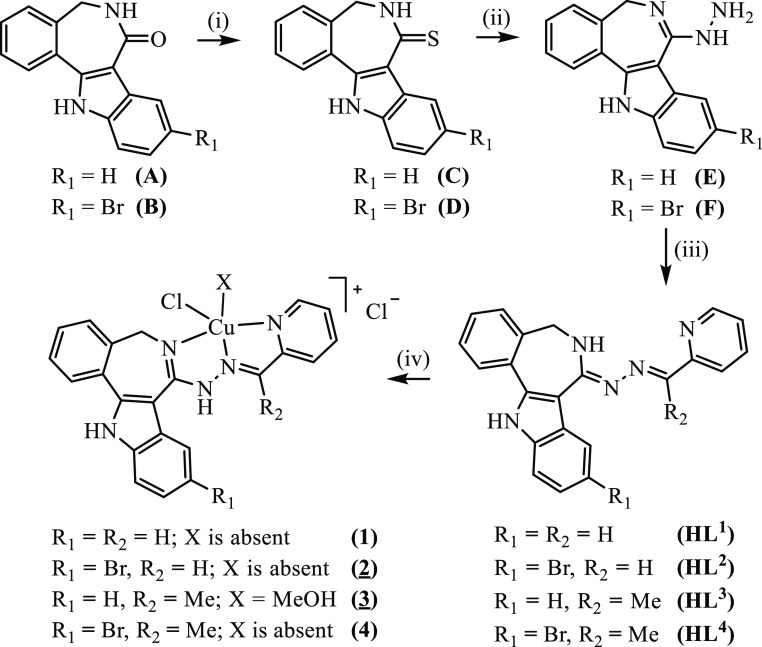
Synthetic Pathway to **HL**^**1**^–**HL**^**4**^ and **1**–**4** Underlined numbers indicate compounds studied by X-ray diffraction. Reagents and conditions: (i) P_4_S_10_/Al_2_O_3_, THF_abs_, 75 °C, overnight; (ii) freshly distilled N_2_H_4_, reflux, overnight; (iii) **HL**^**1**^, **HL**^**2**^: 2-formypyridine, MeOH, 75 °C, overnight; **HL**^**3**^, **HL**^**4**^: 2-acetylpyridine, MeOH, 75 °C, overnight; (iv) CuCl_2_·2H_2_O, MeOH, reflux, 30 min. Interstitial solvent molecules are not shown, but have been specified in the Experimental part.

**Chart 2:**
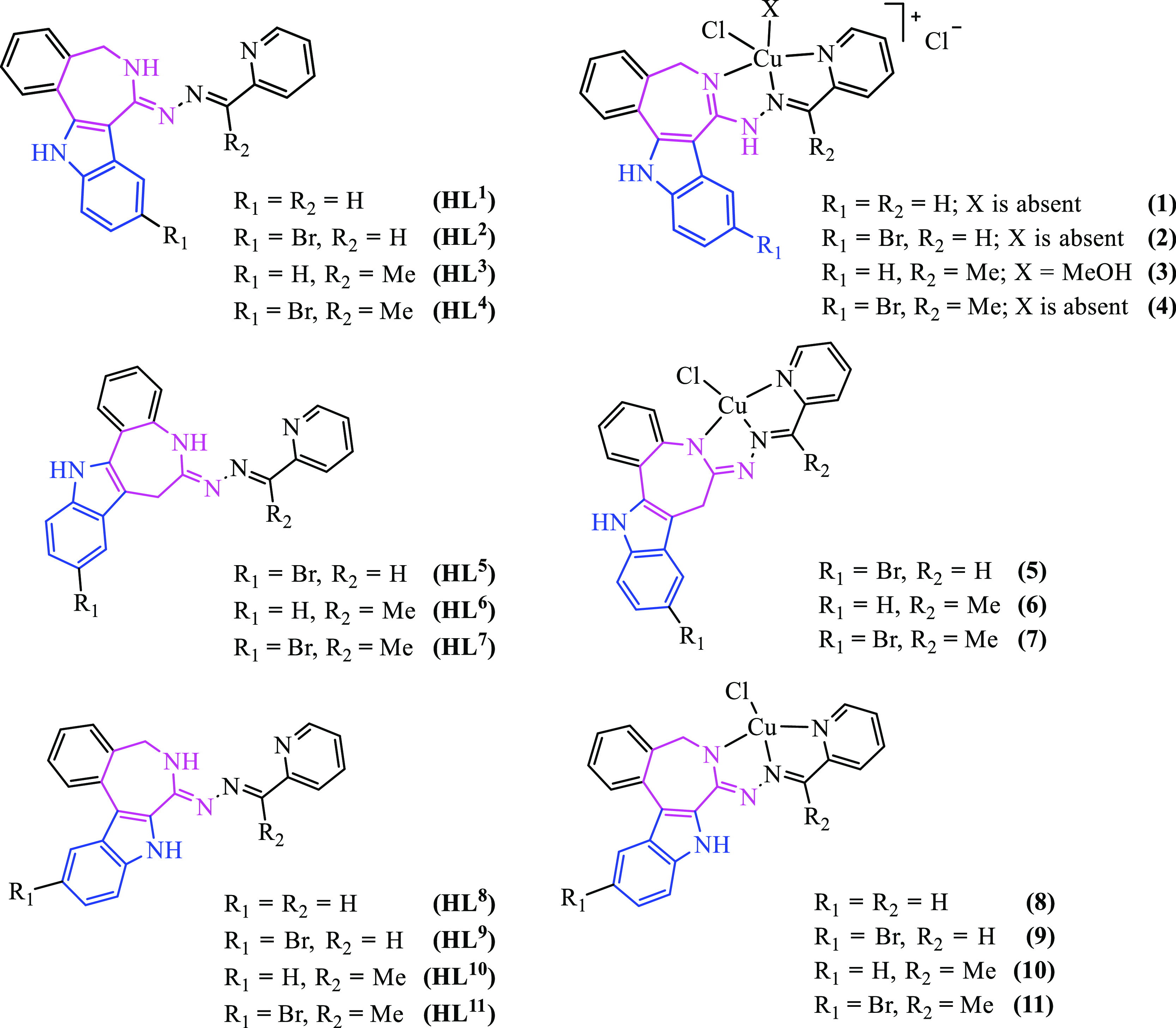
Compounds Studied in this Work.

Previously, we attempted to deduce structure–activity relationships (SARs) by comparing structurally similar paullone-derived (Chart 1, left) and latonduine-derived (Chart 1, right) scaffolds.^33^ However, latonduines differ from paullones by both the flipped indole moiety and the position of the lactam group; therefore, the main shortcoming of our previous study was the modification of two structural features at once. Hence, the effect of each of these two structural changes on the anticancer properties of the resulting compounds, namely, of the flip of the indole moiety and the position of the lactam group in the azepine ring, remained unclear.^33^ Therefore, in this work, we designed four Schiff bases **HL**^**1**^–**HL**^**4**^ (Scheme 1) based on the indolo[3,2-*d*][2]benzazepine scaffold (Chart 1, middle) and compared them with structurally similar compounds **HL**^**5**^–**HL**^**7**^ (Chart 2) based on the indolo[3,2-*d*][1]benzazepine (paullone) scaffold, which differs only by the position of the lactam group (Chart 1, left), and **HL**^**8**^–**HL**^**11**^ (Chart 2) based on the indolo[2,3-*d*][2]benzazepine (latonduine) scaffold (Chart 1, right), which differs only by the flipped indole moiety.

Additionally, their copper(II) complexes **1**–**4**, **5**−**7**, and **8**−**11** were compared under the same experimental conditions. The comparison of indolo[3,2-*d*][2]benzazepine-, latonduine-, and paullone-derived ligands and their copper(II) complexes allowed for a more ample elucidation of SARs. Moreover, we showed that the anticancer activity of one of the most cytotoxic copper(II) complexes **4** was linked to the induction of ROS insult and severe ER stress.

## Results and Discussion

### Synthesis and Characterization of Ligands and Copper(II) Complexes

The synthesis of the main core structure **A** (Scheme 1) was performed by following the published literature protocols.^37^

**Scheme 2:**
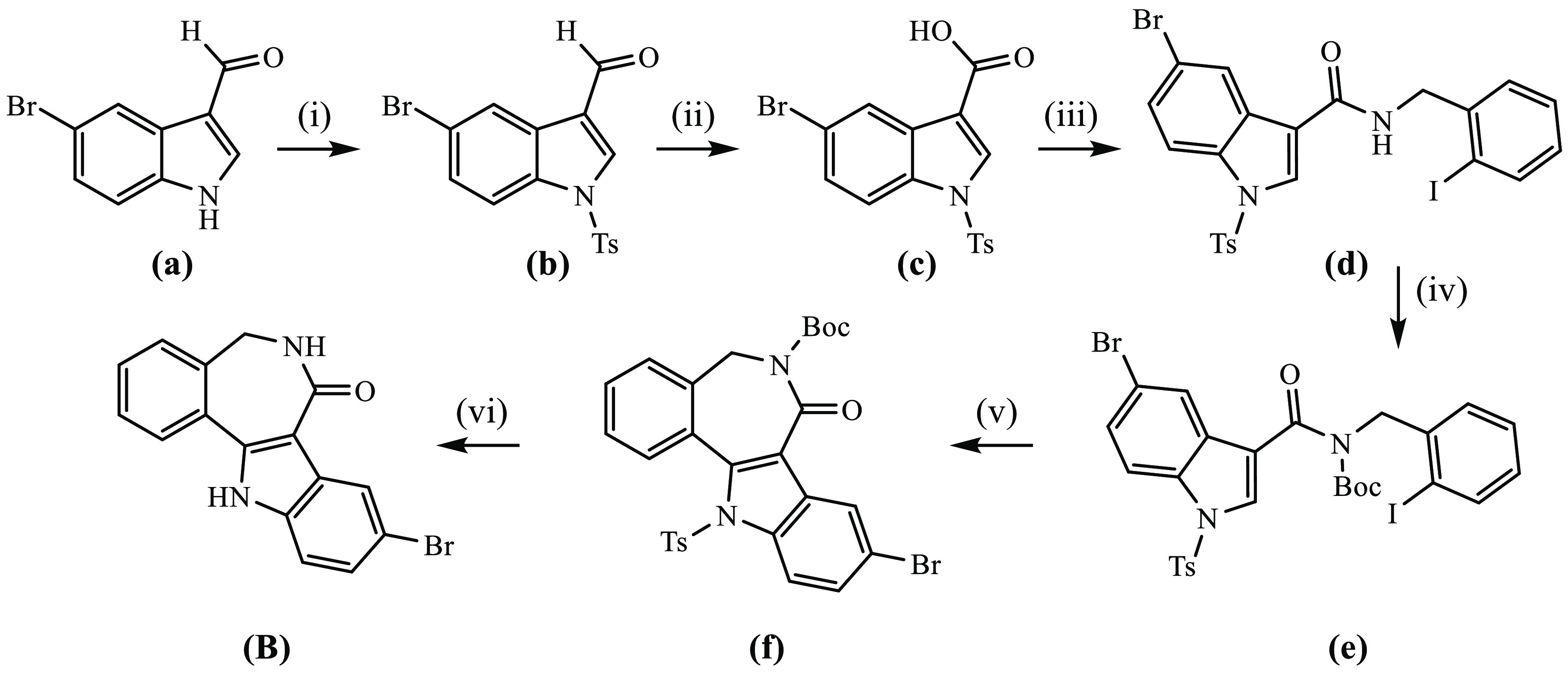
Synthetic Pathway to 11-Bromo-5,12-dihydroindolo[3,2-*d*]benzazepin-7(6*H*)-one (**B**) Reagents and conditions: (i) TsCl, Et_3_N, DCM, rt, 16 h; (ii) NaClO_2_, H_3_NSO_3_, ACN, rt, 15 min; (iii) 2-iodobenzylamine, DMAP, EDCI·HCl, DCM, 0 °C for 4 h, room temperature for 20 h; (iv) Boc_2_O, DMAP, DCM, rt, 12 h; (v) PPh_3_, Pd(OAc)_2_, Ag_2_CO_3_, DMF, 75 °C, 2.5 h. (vi) TFA, DCM, rt, 2 h; TBAF, THF, reflux, 30 min.

The new core structure **B** was obtained similarly by using 5-bromoindole-3-carboxaldehyde as the starting material (Scheme 2). First, the nitrogen atom of 5-bromoindole-3-carboxaldehyde was protected by the reaction with 4-toluenesulfonyl chloride and triethylamine in tetrahydrofuran (THF) in 92% yield. In the next step, species **b** was oxidized to 5-bromo-1-tosyl-1*H*-indole-3-carboxylic acid **c** by using excess sodium chlorite and sulfamic acid in THF in 74% yield. Then, by reacting **c** with 2-iodobenzylamine in dry dichloromethane (DCM) in the presence of 4-dimethylaminopyridine (DMAP) and 1-ethyl-3-(3-dimethylaminopropyl)carbodiimide hydrochloride (EDCI·HCl), compound **d** was prepared in 64% yield. The protection of the amide nitrogen of **d** with di-*tert*-butyldicarbonate (Boc_2_O) in the presence of a catalytic amount of DMAP in dry acetonitrile afforded **e** in 90% yield. Next, ring-closure reaction of **e** in the presence of palladium(II) acetate, triphenylphosphine, and silver(I) carbonate in dry dimethyl formamide (DMF) delivered **f** in 72% yield. The core structure **B** was obtained by removing both the tosyl group by reacting with trifluoroacetic acid and the Boc-protecting group by treatment with excess tetra-*n*-butylammonium fluoride (TBAF) in situ in 54% yield. The lactam derivatives **A** and **B** were further converted into thiolactams **C** and **D** by reaction with phosphorus pentasulfide and aluminum oxide^38^ in dry boiling THF in 60% yields (Scheme 1). In the next step, by using absolute hydrazine as the reagent and solvent, species **E** and **F** were obtained in 37 and 79% yields, respectively. The ^1^H NMR spectra of all isolated intermediate species are displayed in Figures S1–S10 in the Supporting Information. The Schiff bases **HL**^**1**^–**HL**^**4**^ were prepared in 91, 72, 72, and 60% yields, respectively, from **E** and **F** and 2-formyl- and 2-acetylpyridine in boiling methanol. ^1^H and ^13^C NMR spectra of **HL**^**1**^–**HL**^**4**^ (Figures S11–S18) were in agreement with the proposed formulae and their *C*_1_ molecular symmetry. Electrospray ionization (ESI) mass spectra measured in the positive ion mode (Figures S19–S22) showed peaks with *m*/*z* 352.25, 432.18, 366.29, and 446.20 attributed to the [M + H]^+^ ions. Complexes **1**–**4** were synthesized by reaction of **HL**^**1**^–**HL**^**4**^ in methanol with a methanolic solution of CuCl_2_·2H_2_O in a 1:1 molar ratio in 77 to 93% yields. The purity of **HL**^**1**^–**HL**^**4**^ (>95%) was confirmed by elemental analysis and NMR spectroscopy, while that of **1**–**4** by elemental analysis. In addition, the purity of **4** was evidenced by HPLC coupled with high-resolution ESI mass spectrometry (HR ESI MS) (Figure S23). The positive ion ESI mass spectrum of **1** showed peaks with *m*/*z* 449.17 and 863.16 due to [Cu^II^Cl(HL^1^)]^+^ and {[Cu^II^Cl(L^1^)][Cu^II^(L^1^)]}^+^, respectively (Figure S24). The HR ESI mass spectra of **2** and **4** contain peaks with *m*/*z* 246.9927 and 254.0008, 528.9539 and 542.9702 attributed to [Cu^II^(HL^2^)]^2+^ and [Cu^II^(HL^4^)]^2+^, [Cu^II^Cl(HL^2^)]^+^ and [Cu^II^Cl(HL^4^)]^+^, respectively (Figures S25 and S27). The HR ESI mass spectrum of **3** contains a peak with *m*/*z* 427.0856 due to [Cu^II^(L^3^)]^+^ (Figure S26).

Further evidence for the formation of copper(II) complexes with the prepared Schiff bases and disclosure of the coordination geometry adopted by copper(II) were obtained from single-crystal X-ray diffraction studies.

### X-ray Crystallography

The results of X-ray diffraction study of intermediate species **f**, complexes [**CuCl**(**HL**^**2**^)]**Cl** (**2**), [**CuCl**(**HL**^**3**^)]**Cl**, [**CuCl**(**HL**^**3**^)(**MeOH**)]**Cl**·**MeOH**, and [**CuCl**(**L**^**3**^)]·**EtOH** are shown in Figures S28 (Supporting Information), **1** and **2**, respectively. Single crystals of [**CuCl**(**HL**^**2**^)]**Cl** and [**CuCl**(**HL**^**3**^)(**MeOH**)]**Cl**·**MeOH** were grown from methanol, those of [**CuCl**(**HL**^**3**^)]**Cl** from a DMF solution layered with diethyl ether, and those of [**CuCl**(**L**^**3**^)]·**EtOH** from ethanol in the presence of Et_3_N as a base. Selected bond distances (Å) and bond angles (deg) are quoted in the legends to figures. The structure of [**CuCl**(**HL**^**2**^)]**Cl** (**2**) is built up of linear μ-chlorido-bridged one-dimensional coordination polymers (Figure S29). In the chain, each Cu(II) ion adopted a 4+1 distorted square-pyramidal coordination geometry.

**Figure 1:**
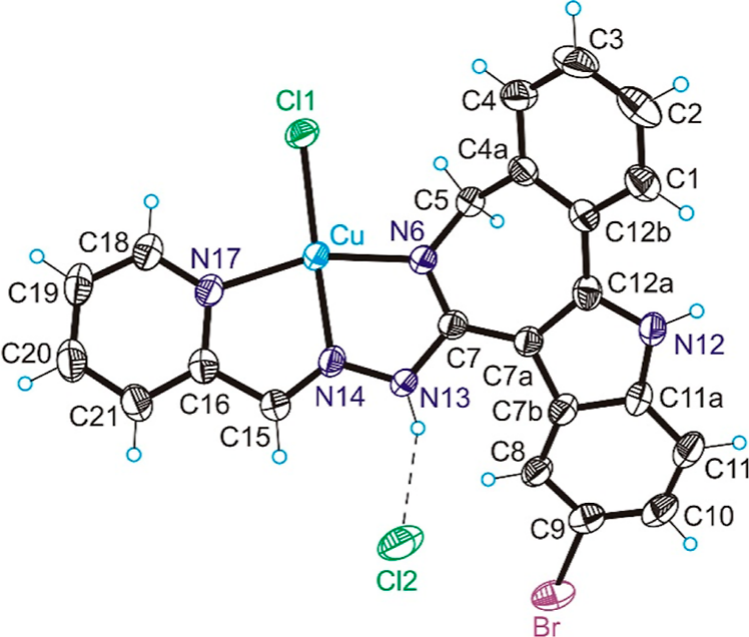
ORTEP views of **2** with thermal ellipsoids at 40% probability level. Selected bond distances (Å) and bond angles (deg): (a) Cu–N6 = 1.953(3), Cu–N14 = 1.958(5), Cu–N17 = 2.041(5), Cu–Cl1 = 2.2399; N6–Cu–N14 = 80.0(2), N14–Cu–N17 = 79.4(2); Θ_C7a–C12a–C12b–C4a_ = −31.1(9).

The tridentate neutral ligand **HL**^**2**^ occupies three coordination sites in the base of the pyramid of copper(II) via the azepine nitrogen atom N6, the hydrazinic nitrogen N14, and the pyridine nitrogen N17. The fourth coordination site is filled by the chlorido coligand Cl1. The apical site is occupied by the chlorido coligand from the neighboring complex. The bond length from Cu(II) to the basal chlorido coligand is 0.464 Å shorter than that to apical chlorido coligand provided by the adjacent molecule. The overall positive charge of each individual copper(II) complex [**CuCl**(**HL**^**2**^)]^+^ is counterbalanced by the chloride counteranion, which acts as a proton acceptor in hydrogen bonding with the N13–H group as shown in Figure 1.

One feature of note is the folding of the ligand backbone due to the presence of one sp^3^-hybridized carbon atom in the seven-membered azepine ring. This is also the case for related complexes with paullone ligands and latonduines.^33,39^ The dihedral angle between the mean plane through benzene ring atoms C1–C2–C3–C4–C4a–C12b and Cu(II) mean coordination plane through N6–N14–N17–Cu is 128.7(2)°.

**Figure 2:**
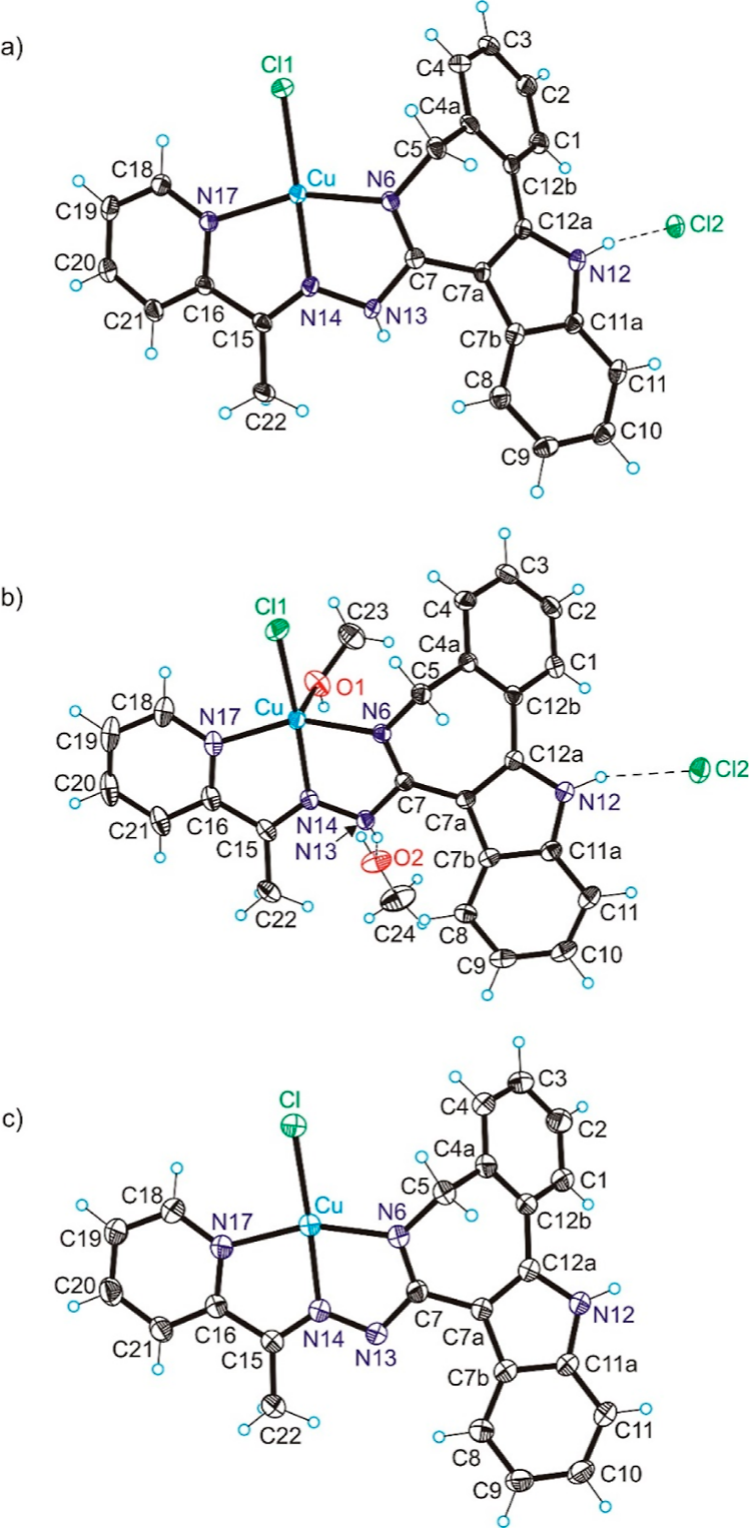
ORTEP views of (a) [**CuCl**(**HL**^**3**^)]**Cl**, (b) [**CuCl**(**HL**^**3**^) (**MeOH**)]**Cl**·**MeOH** (**3**·**MeOH**), and (c) [**CuCl**(**L**^**3**^)]·**EtOH** with thermal ellipsoids at 50% probability level. Selected bond distances (Å) and bond angles (deg): (a) Cu–N6 = 1.965(3), Cu–N14 = 1.950(3), Cu–N17 = 2.022(3), Cu–Cl = 2.2016(8); N6–Cu–N14 = 79.56(11), N14–Cu–N17 = 79.48(11); Θ_C7a–C12a–C12b–C4a_ = 31.3; (b) Cu–N6 = 2.0022(15), Cu–N14 = 1.9590(15), Cu–N17 = 2.0396(16), Cu–Cl = 2.2113(5), Cu–O1 = 2.2625(15); N6–Cu–N14 = 78.80(6), N14–Cu–N17 = 78.70(7); Θ_C7a–C12a–C12b–C4a_ = 34.2(3); (c) Cu–N6 = 1.954(2), Cu–N14 = 1.946(2), Cu–N17 = 2.031(3), Cu–Cl = 2.2186; N6–Cu–N14 = 79.40(10), N14–Cu–N17 = 79.90(10); Θ_C7a–C12a–C12b–C4a_ = 32.4(5).

**Figure 3:**
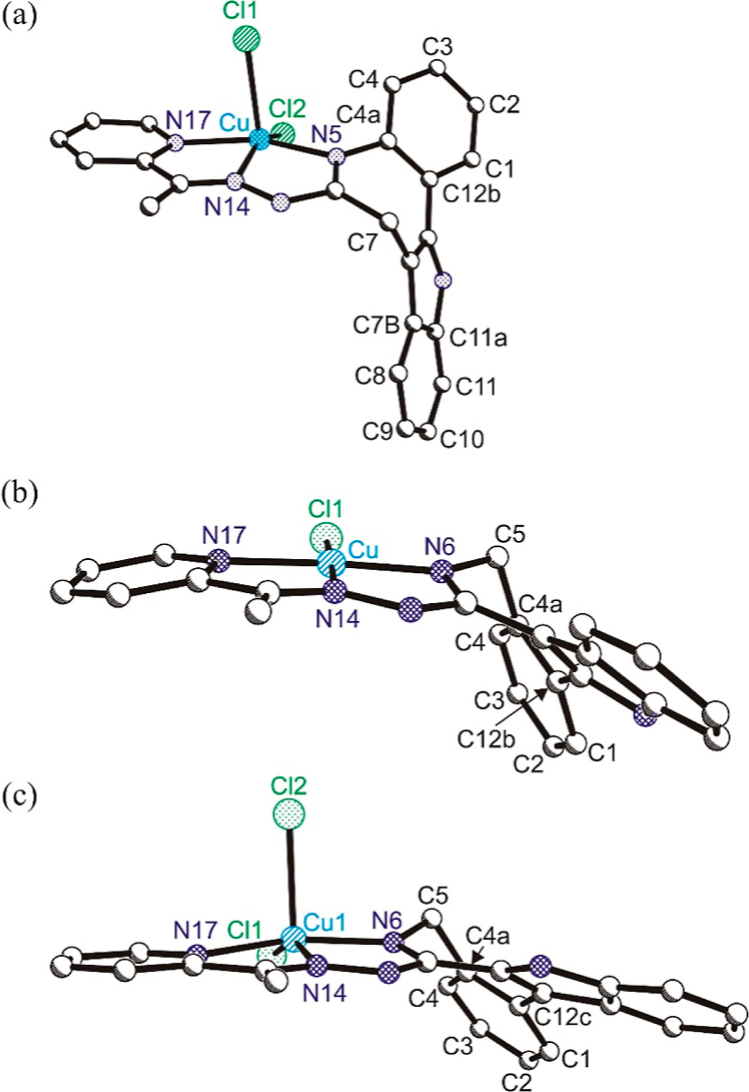
Comparative effects of sp^3^-hybridized carbon atom (C7 or C5) in the seven-membered azepine ring on the folding of the indolo[3,2-*d*][1]benzazepine (paullone) backbone in complex [**CuCl**_**2**_(**HL**^**6**^)] (**6**) (a), indolo[3,2-*d*][2]benzazepine core in [**CuCl**(**HL**^**3**^)]**Cl** (b), and indolo[2,3-*d*][2]benzazepine (latonduine) backbone in [**CuCl**_**2**_(**HL**^**10**^)] (**10**) (c).

Like **2**, the complex [**CuCl**(**HL**^**3**^)]**Cl** (Figure 2a) has a square-planar coordination geometry. One marked difference is the value of the dihedral angle between mean planes through C1–C2–C3–C4–C4a–C12b and N6–N14–N17–Cu which is now 113.69(9)°. The folding of the coordinated ligand **HL**^**3**^ is compared with that of coordinated paullone **HL**^**6**^ and coordinated latonduine **HL**^**10**^ in Figure 3. The strongest folding is seen in the complex with paullone ligand [**CuCl**_**2**_(**HL**^**6**^)] (**6**) taking place about the line going through atom C7 and the middle of the opposite bond C4a–C12b. The dihedral angle between mean planes through C7b–C8–C9–C10–C11–C11a and N5–N14–N17–Cu is 101.31(5)°. The folding of latonduine **HL**^**10**^ in the complex [**CuCl**_**2**_(**HL**^**10**^)] (**10**) is the smallest. The dihedral angle between mean planes through C1–C2–C3–C4–C4a–C12c and N6–N14–N17–Cu is 127.17(15)° and very close to that in **2** (vide supra).

The complex [**CuCl**(**HL**^**3**^)(**MeOH**)]**Cl**·**MeOH** in contrast to [**CuCl**(**HL**^**3**^)]**Cl** is five-coordinate and is prone to adopt a coordination geometry, which is closer to square-pyramidal than to trigonal bipyramidal (Figure 2b). The τ_5_-value is 0.28 (τ_5_ = 0 for a square pyramid and 1.00 for a trigonal bipyramid).^40^ The neutral tridentate ligand **HL**^**3**^ and chlorido coligand Cl1 are bound to Cu(II) in the base of the pyramid, and a molecule of methanol is coordinated in the apical position. As expected, an expanding of coordination sphere is observed when going from four-coordinate to five-coordinate species due to the increase of interatomic repulsions. The bond lengths in the five-coordinate complex in Figure 2b are markedly longer when compared to the four-coordinate complex in Figure 2a (see legend to Figure 2).

The dihedral angle between mean planes through C1–C2–C3–C4–C4a–C12b and N6–N14–N17–Cu is 128.76(5)°. We have also noticed pyramidalization of the atom N13 in [**CuCl**(**HL**^**3**^)]**Cl** and [**CuCl**(**HL**^**3**^)(**MeOH**)]**Cl**·**MeOH**, which is more pronounced in the square-planar complex. The sum of bond angles around N13 deviates markedly from 360°, being 343.9° in the square-planar complex (Figure 2a) and 348.0° in the square-pyramidal species (Figure 2b).

Complex [**CuCl**(**L**^**3**^)]·**EtOH** (Figure 2c) is square-planar. The organic ligand acts as a tridentate monoanion, coordinating to copper(II) via nitrogen atoms N6, N14, and N17 (Figure 2c). The fourth coordination site is occupied by the chlorido coligand. The dihedral angle between mean planes through C1–C2–C3–C4–C4a–C12b and N6–N14–N17–Cu in [**CuCl**(**L**^**3**^)]·**EtOH** is 124.79(9)°.

### Molecular Descriptors for HL^1^–HL^4^ and Complexes **1–4**

The lipophilicity and aqueous solubility of both organic compounds as potential ligands and their metal complexes are two important pharmacokinetic parameters, the first determining their ability to cross the plasmatic membrane and reach the intracellular environment and the second predicting the absorption and distribution of the drug in the body. In other words, both the parameters help in the assessment of drug-likeness. Several physico-chemical parameters, including log *P* and log *S*, have been calculated for **HL**^**1**^**–HL**^**4**^ and complexes **1–4** by using the pharmacokinetic program SwissADME and are presented in Table S1.^41^ As can be seen from Table S1, all compounds studied in this work have a molecular weight lower but close to 500 g/mol or slightly exceeding this value (complex **2**) and lie mostly within the druglike chemical space. The octanol/PBS (pH = 7.4) partition coefficient log *P* for copper(II) complexes was assessed by the shake-flask procedure^42^ indicating their hydrophobic nature. The calculated log *P* of complex **4** was 4.23 and according to Lipinski’s rules can be considered as regular lipophilic (4 < log *P* < 5). The other three complexes were found less lipophilic than **4**. HD (hydrogen bond donors) and HA (hydrogen bond acceptors) in all compounds studied are also in accordance with Lipinski’s rules. The predicted aqueous solubility of **HL**^**1**^**–HL**^**4**^ can be characterized as moderate (−5 < log *S* < −4). As noticed recently,^43^ the online program used fails to predict reliably the aqueous solubility of metal complexes. The calculations predict the poor solubility for **1–3** and moderate for complex **4**. In fact, as determined experimentally, all four complexes are soluble in water containing 1% DMSO at 1 mg mL^–1^ (∼2 mM) concentration corresponding to log *S* ∼ −2.7.

### Stability Studies

First, UV–vis kinetic studies were performed with the ligands **HL**^**2**^ and **HL**^**4**^ (Figure S30) and the copper(II) complexes **1–4** in aqueous solutions containing 1% DMSO (Figures S31 and 32). Additionally, the stability of the copper(II) complex **4** was also measured in phosphate-buffered saline (PBS) (pH ∼ 7.4) containing 3% DMSO over 24 h. All compounds studied demonstrated excellent stability as no changes in the UV–vis absorption spectra over 72 h were observed. In addition, ESI mass spectra of **4** in 1% DMSO/water and in PBS containing 3% of DMSO measured directly after dissolution and 24 h later have confirmed that the compound remained intact under these conditions. Additional peaks which could be assigned to the dissociated ligand or any its fragments due to a hypothetical ligand degradation have not been registered. In addition to UV–vis data, the stability and purity of complex **4** were tested by analytical HPLC-HR ESI MS using methanol or acetonitrile with 0.1% formic acid as the eluent over 10 min. A single peak at around 1 min corresponding to [Cu^II^(L^4^)]^+^ (found *m*/*z* = 506.9939 (Figure S23), calcd *m*/*z* for C_23_H_17_BrCuN_5_ 507.0063) was registered in agreement with other experiments. In addition, the stability of **HL**^**4**^ in 1:1 DMSO-*d*_6_/D_2_O at 25 °C was monitored by ^1^H NMR spectroscopy. The spectra measured immediately after dissolution, 1 h later, and after 24 h did not show any changes attesting their stability in aqueous DMSO solution (Figure S33). Based on these data, we concluded that the compounds did not undergo any transformations in aqueous solution over 72 h, as was also the case for compounds **HL**^**5**^–**HL**^**7**^ and complexes **5**–**7**, as well as for **HL**^**8**^–**HL**^**11**^ and **8**–**11**, reported previously, and they are suitable for the in vitro experiments. In addition, solution stability of the lead complex **4** was studied in ethanol-cell culture medium (1:1 v/v) by monitoring the electronic absorption spectra in the visible region within a time period of 8 h (Figure S34, Supporting Information). The complex was fairly stable up to 8 h and showed no remarkable change in the absorption intensity within the given time period. In addition, positive ion ESI mass spectrum of the complex after 8 h showed a peak with *m*/*z* 443 due to [CuCl(HL^4^)]^+^ (Figure S35), providing further evidence of its stability in ethanol-cell culture medium.

### Antiproliferative Activity

The in vitro antiproliferative activity of novel organic compounds **HL**^**1**^–**HL**^**4**^ and corresponding copper(II) complexes **1**–**4** was tested in the breast cancer cell line MDA-MB-231, hepatocellular carcinoma cell line LM3, and human embryonic kidney cell line HEK293 and then compared to those of the previously reported paullones **HL**^**5**^–**HL**^**7**^ and copper(II) complexes **5**–**7**,^39,44^ as well as latonduines **HL**^**8**^–**HL**^**11**^ and copper(II) complexes **8**–**11**.^34^ The two cancer cell lines were reported to exhibit quite aggressive behavior in patients, high rates of metastasis, and proliferation.^45,46^ The in vitro anticancer activity was determined by the colorimetric MTT assay with an exposure time of 72 h. The IC_50_ values of compounds of interest are listed in Table 1. All compounds showed high antiproliferative activity with IC_50_ values from the low micromolar to the submicromolar and even nanomolar concentration range, which is superior to that exhibited by cisplatin as positive control. The highest cytotoxicity in the two cancer cell lines MDA-MB-231 and LM3 revealed the organic compounds **HL**^**4**^ and **HL**^**11**^, both Schiff bases being methylated at the Schiff base double bond and brominated at position 9 and position 11 of indolo[3,2-*d*][2]benzazepine and latonduine backbone, respectively. Compound **HL**^**4**^ showed higher selectivity for these two cancer cell lines when compared to the noncancerous cell line HEK293 than the latonduine derivative **HL**^**11**^. Both compounds showed superior activity and better selectivity for the two cancer cell lines compared to the paullone derivative **HL**^**7**^. Analogously, the copper(II) complex of **HL**^**4**^ (complex **4** in Table 1) revealed higher antiproliferative activity than the copper(II) complex of paullone **HL**^**7**^ (complex **7** in Table 1) but lower activity than the copper(II) complex of latonduine **HL**^**11**^ (complex **11** in Table 1) in the two cancer cell lines.

**Table 1:** 50% Inhibitory Concentrations (IC_50_, μM) of **HL**^**1**^–**HL**^**11**^ and **1**–**11** in Comparison with Cisplatin Determined by the MTT Assay after Exposure for 72 h

type	compound	MDA-MB-231	LM3	HEK293
indolo-benzazepine	**HL**^**1**^	1.5 ± 0.1	1.4 ± 0.2	2.0 ± 0.1
	**HL**^**2**^	1.2 ± 0.1	0.87 ± 0.16	0.97 ± 0.19
	**HL**^**3**^	0.17 ± 0.03	0.10 ± 0.03	0.18 ± 0.01
	**HL**^**4**^	0.16 ± 0.02	0.07 ± 0.01	0.26 ± 0.07
paullone	**HL**^**5**^	0.91 ± 0.30	0.71 ± 0.21	0.03 ± 0.00
	**HL**^**6**^	0.35 ± 0.05	0.28 ± 0.02	0.15 ± 0.03
	**HL**^**7**^	0.33 ± 0.08	0.25 ± 0.07	0.05 ± 0.02
latonduine	**HL**^**8**^	1.6 ± 0.2	0.98 ± 0.05	0.82 ± 0.05
	**HL**^**9**^	0.70 ± 0.22	0.59 ± 0.06	0.13 ± 0.03
	**HL**^**10**^	0.19 ± 0.01	0.13 ± 0.01	0.17 ± 0.01
	**HL**^**11**^	0.13 ± 0.03	0.11 ± 0.01	0.15 ± 0.02
Cu(II)-indolobenzazepine	**1**	1.9 ± 0.3	1.8 ± 0.3	1.6 ± 0.1
	**2**	1.8 ± 0.4	1.2 ± 0.0	1.1 ± 0.1
	**3**	0.18 ± 0.02	0.10 ± 0.00	0.12 ± 0.03
	**4**	0.22 ± 0.04	0.11 ± 0.02	0.11 ± 0.02
Cu(II)-paullone	**5**	2.8 ± 1.0	2.4 ± 0.2	1.5 ± 0.8
	**6**	0.45 ± 0.04	0.28 ± 0.05	0.33 ± 0.06
	**7**	0.45 ± 0.00	0.41 ± 0.01	0.30 ± 0.08
Cu(II)-latonduine	**8**	1.1 ± 0.3	0.91 ± 0.09	1.5 ± 0.0
	**9**	0.81 ± 0.26	0.73 ± 0.09	0.17 ± 0.02
	**10**	0.16 ± 0.01	0.09 ± 0.02	0.06 ± 0.01
	**11**	0.11 ± 0.02	0.08 ± 0.02	0.11 ± 0.01
	**cisplatin**	21 ± 5^a^	10 ± 3^a^	3.4 ± 1.1^b^

a Data taken from ref (43).

b Data taken from ref (47).

Coordination of **HL**^**4**^, **HL**^**7**^, and **HL**^**11**^ to copper(II) resulted in a slight decrease of cytotoxicity in cancer cell lines, and, in addition, the loss of selectivity for MDA-MB-231 and LM3 cancer cell lines when compared to noncancerous HEK293 cells. However, coordination of these ligands to copper(II) might induce a new mechanism of action.^48^ In particular, fully distinct inhibitory profiles in enzyme inhibition assays were disclosed recently for indolo[3,2-*c*]quinoline-based ligand and its copper(II) complex,^34^ highlighting a special role of copper(II) in the underlying mechanism of cytotoxicity. The anticancer activity of copper(II) complexes **1**–**4** is mostly due to their redox activity (when compared to redox silent ligands **HL**^**1**^–**HL**^**4**^) which resulted in the generation of cytotoxic ROS and induction of ER stress (vide infra).

The cytotoxic activities of complexes **1**–**4** were superior or comparable in terms of IC_50_ values to those of ER-targeting copper(II) complexes with bis-pyrazole and pyrazole-pyridine derivatives,^49^ dithiocarbamates,^50^ as well as mixed-ligand copper(II) complexes targeting mitochondria.^51,52^ The most active new compounds reported in this work **HL**^**4**^ and **4** were selected for further biological studies in order to get more insight into the underlying mechanism of their antiproliferative activity.

### ROS Detection by Fluorescent Microscopy

**Figure 4:**
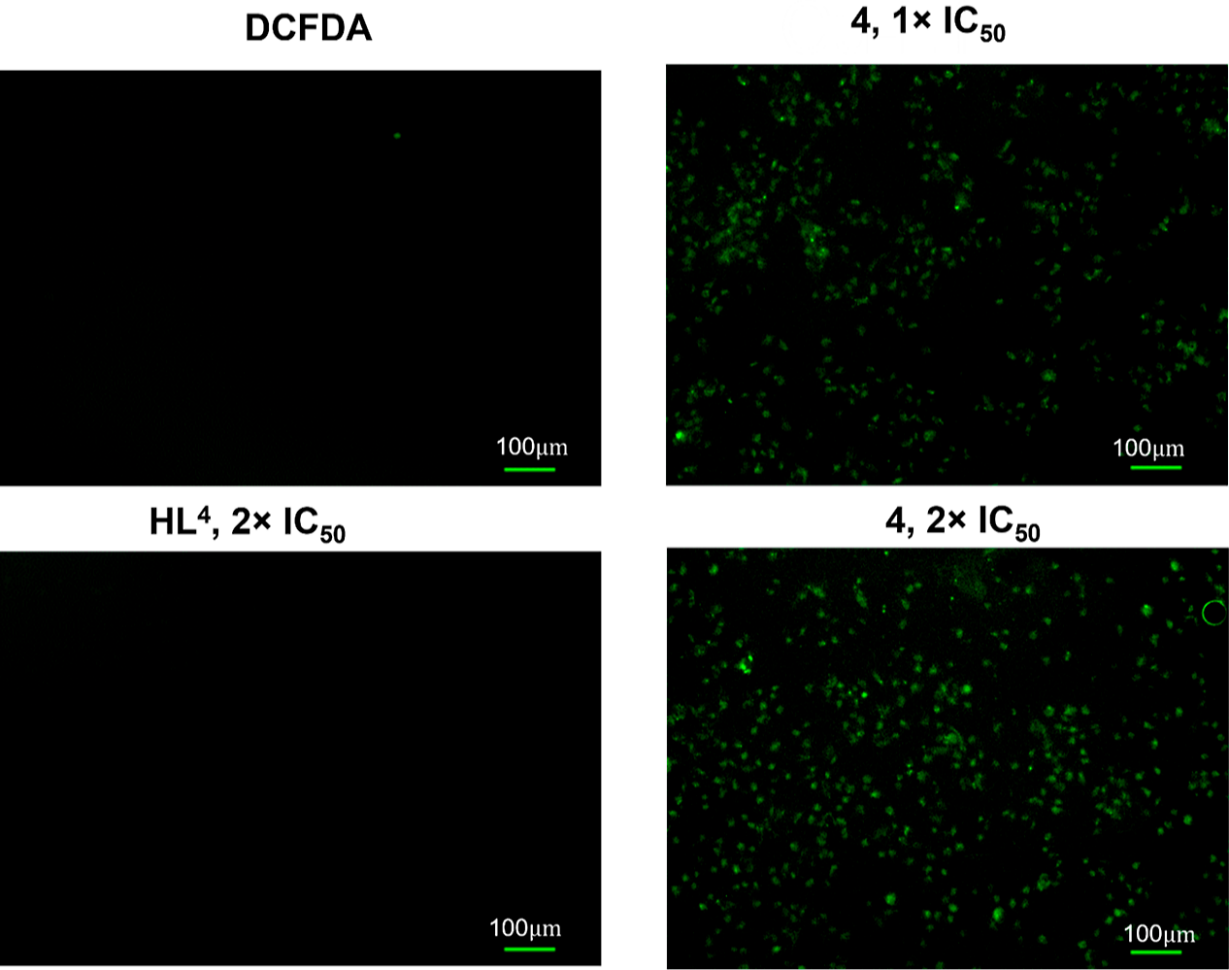
ROS was visualized by staining MDA-MB-231 cancer cells with the H_2_DCFDA dye. ROS generation was observed under a fluorescence microscope after 4 h incubation of the samples with different concentrations of **4** (0.25 and 0.5 μM) and **HL**^**4**^ (0.4 μM). Scale bar = 100 μm.

ROS are produced due to one or more electron reduction of oxygen by cellular enzymes or in the mitochondrial respiratory pathway, even though there are also other sources of endogenous ROS, that is, Fenton-like reactions. Oxygen molecule (O_2_), superoxide anion radical (O_2_^–•^), hydroxyl free radical (HO^•^), and hydrogen peroxide (H_2_O_2_) are all examples of ROS.^53^ Multiple ways for boosting cancer cells’ intracellular ROS levels appeared to be therapeutically advantageous. It was recently reported^54,55^ that the most effective anticancer drugs nowadays widely used in clinics are ROS inducers. To address the issue about the role of Cu in the generation of intracellular damaging ROS in the triple-negative breast cancer cell line (MDA-MB-231), we compared the effects of complex **4** and its corresponding ligand **HL**^**4**^. As expected, complex **4** induced dose-dependent ROS generation. The strongest ROS insult was observed when cells were treated with 2× IC_50_ of **4**. On the contrary, the ligand **HL**^**4**^, even at 2× IC_50_, did not induce ROS production (Figure 4). Quantification of ROS generation is shown in Figure S36 in the Supporting Information.

### EPR Spin-Trapping Experiments

**Figure 5:**
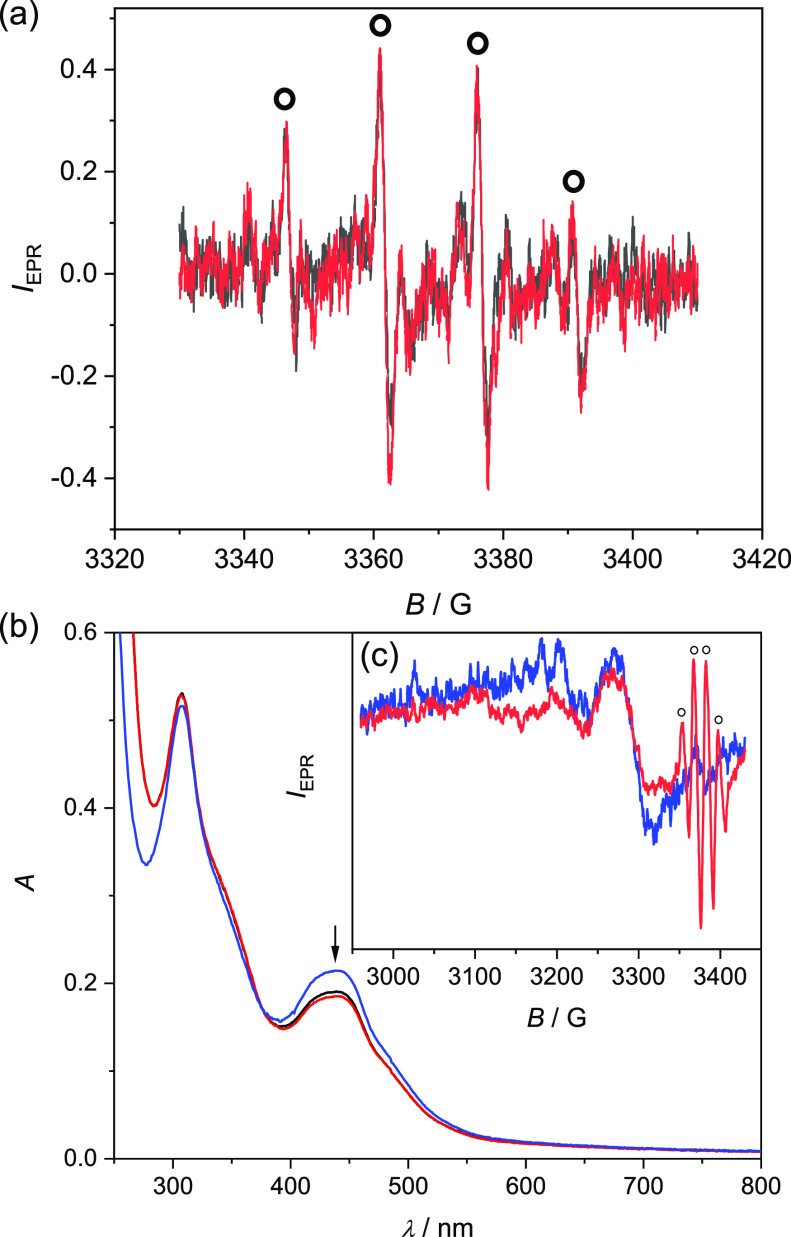
(a) EPR spectra monitored 5 min (black trace) and 10 min (red trace) after the addition of H_2_O_2_ to the aqueous 5% DMSO (v/v) solution of **4** under air in the presence of the spin-trapping agent DMPO. Initial concentrations: *c*_0_(**4**) = 0.5 mM, *c*_0_(DMPO) = 0.02 M, *c*_0_(H_2_O_2_) = 0.02 M. (b) UV–vis spectra monitored 5 min (black trace) and 10 min (red trace) after the addition of H_2_O_2_ to the aqueous 1% DMSO (v/v) solution of **4** under air in the presence of the spin-trapping agent DMPO. Initial concentrations: *c*_0_(**4**) = 50 μM, *c*_0_(DMPO) = 0.02 mM, *c*_0_(H_2_O_2_) = 0.02 mM; reference UV–vis spectrum of **4** where in an analogous experiment deionized water was added instead of aqueous solutions of H_2_O_2_ and DMPO (blue trace). (c) EPR spectra monitored for the reference solution of **4** under air (black trace, *c*_0_(**4**) = 0.5 mM) and for the solution of **4** under air after the addition of H_2_O_2_ into the aqueous 5% DMSO (v/v) solution of **4** under air in the presence of the spin-trapping agent DMPO (red trace). EPR spectra of ^•^DMPO–OH adducts are marked with circles (initial concentrations: *c*_0_(**4**) = 0.5 mM, *c*_0_(DMPO) = 0.02 M, *c*_0_(H_2_O_2_) = 0.02 M). Experimental parameters: microwave frequency ∼ 9.5 GHz; power of the microwave radiation ∼ 25 mW; modulation amplitude 10 G; 20 scans; room temperature.

By an independent EPR spin-trapping experiment, it was also shown that lead compound **4** is able to generate ROS via the Fenton-like reactions (Figure 5).^56^ The formation of hydroxyl radicals via the Fenton reactions requires hydrogen peroxide, which is produced in living organisms from the superoxide radical anion by manganese superoxide dismutase.^57^

Complex **4** was dissolved in water containing 5% DMSO and mixed with the 5,5-dimethyl-1-pyrroline-*N*-oxide (DMPO) spin-trapping agent under air. The EPR spectra of the prepared reaction mixture were recorded 5 and 10 min after the addition of the hydrogen peroxide into the system. As seen in Figure 5, complex **4** induced ROS generation along with reactive radical intermediates evidenced by the presence of the dominating characteristic four-line EPR signal assigned to the ^•^DMPO–OH spin adduct. Based on the simulation analysis, the main signal belongs to the ^•^DMPO–OH spin adduct (*a*_N_ = 14.8 G, *a*_H_ = 14.3 G; *g* = 2.0057), and the additional low-intensity EPR signal was assigned to the ^•^DMPO-CH_3_ (*a*_N_ = 15.9 G, *a*_H_ = 22.8 G; *g* = 2.0055) originating from reactions of ROS with DMSO.^58^

To provide further evidence that complex **4** is partially reduced to copper(I) species in the spin-trapping assay (presence of H_2_O_2_ and DMPO), UV–vis and EPR spectroscopy were used to monitor the changes in electronic absorption and EPR spectra of copper(II) complex before and after the addition of H_2_O_2_ and DMPO to its 5% DMSO aqueous solution (Figure 5b). A decrease of the characteristic Cu(II) state optical band in the region 400–500 nm after the addition of H_2_O_2_ and DMPO to 5% DMSO aqueous solution of **4** was observed by UV–vis spectroscopy (see black and red traces in Figure 5b). Additionally, a decrease of intensity of a broad EPR signal with *S* = 1/2 for d^9^ copper(II) at room temperature was observed due to the formation of diamagnetic (*S* = 0) cuprous species indicating the occurrence of Fenton-like reaction, leading to the formation of hydroxyl free radicals (see the EPR spectrum of ^•^DMPO-OH adduct marked with circles in Figure 5c).

### Induction of ER Stress

**Figure 6:**
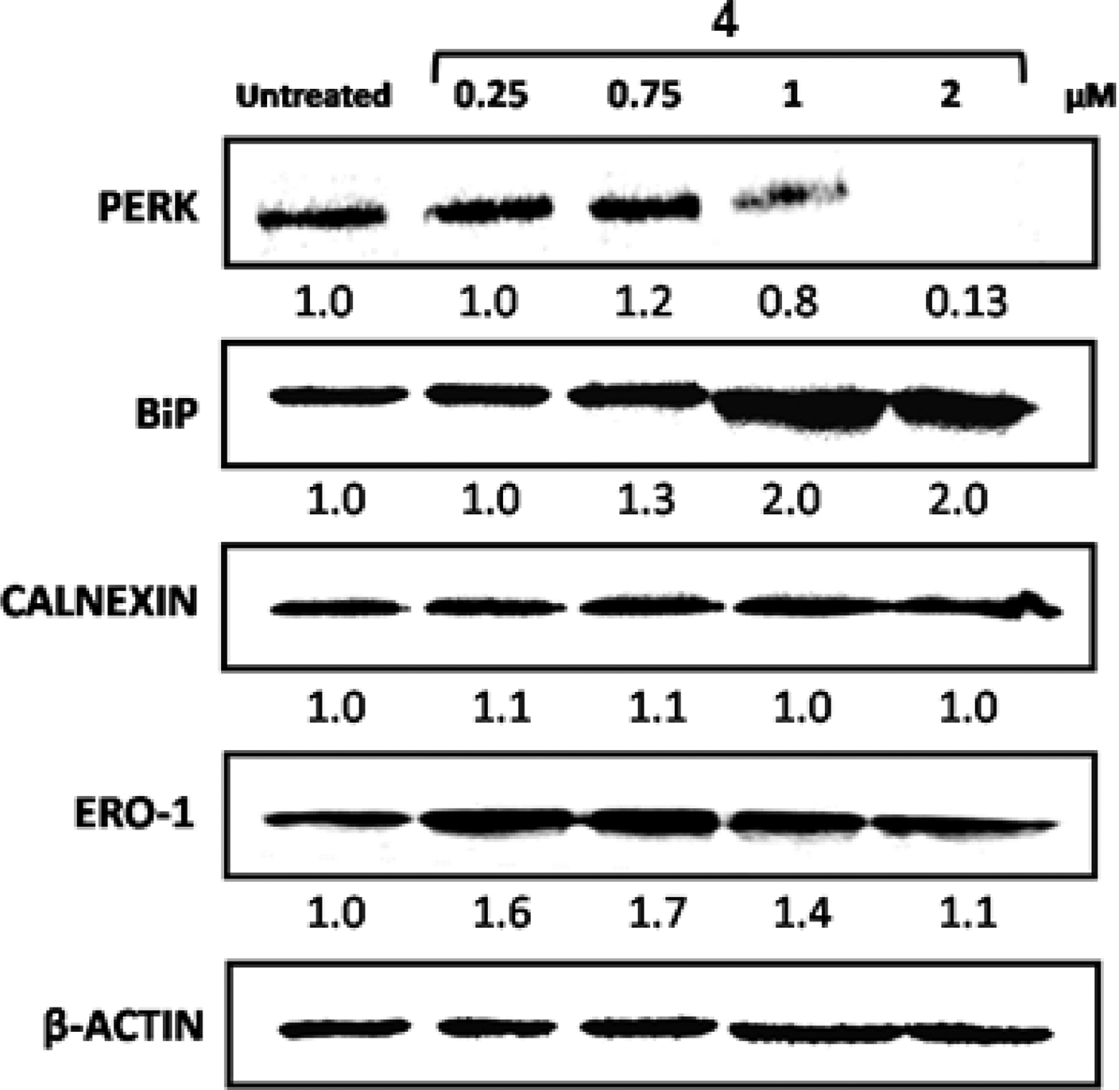
Western blot analysis of various ER stress biomarkers after 24 h treatment with increasing concentrations of **4** (0.25–2 μM). Actin was used as a loading control. Fold change in protein expression is calculated versus the intensity of untreated cells and normalized based on the intensity of respective actin bands.

ER stress is a protective mechanism used by the cells to redress their homeostasis as the level of unfolded or misfolded proteins is increased in the cell.^59^ ER stress has different pathways to restore cellular balance, and one of them, as mentioned previously, is UPR. The main functions of UPR are the reduction of protein translation and activation of degradation of misfolded and unfolded proteins.^60^ The UPR consists of three major pathways: PERK, IRE1, and ATF6.^61^ Herein, we investigated the concentration-dependent effects of **4** on ER stress activation, in particular, PERK, BiP, calnexin, and Ero1-Lα markers using the Western blotting technique. MDA-MB-231 cells were treated with four increasing doses of **4** with respect to IC_50_ values determined from the 72 h MTT experiment. The level of expression of ER markers was compared to that for untreated cells as shown in Figure 6.

It is known that under unstressed conditions, immunoglobin binding protein (BiP), which is an ER chaperone that assists in protein folding, binds to PERK and prevents its activity. Upon ER stress, BiP dissociates from PERK resulting in the activation of the latter by autophosphorylation.^62^ The Western blot results for PERK and BiP supported the activation of PERK as the protein expression significantly decreased with increasing drug dosages. For instance, at the highest drug dose of 2 μM, the PERK intensity was approximately 90% lower than for the control. On the other hand, the intensity of BiP increased 2 times compared to control, indicating its disassociation from PERK and activation of UPR.^63^ Calnexin, another key ER chaperone that binds to nascent glycoprotein, showed no associations with ER stress activation as its expression had no significant difference between different drug-treated and untreated cells. Ero1-Lα is an oxidoreductase enzyme that catalyzes disulfide bond formation, and as a result, H_2_O_2_ is produced. The expression of Ero1-Lα increased at first drug concentrations, 0.25 and 0.75 μM, and then decreased when higher concentrations, 1 and 2 μM, were added. The increase in the Ero1-Lα intensity at the first doses indicates ER stress activation. At the higher concentrations, the level of Ero1-Lα decreased as an adaptive mechanism for cells in severe ER stress conditions because Ero1-Lα activates the production of toxic ROS.^64^

## Conclusions

Development of ER-targeting metal complexes inducing ER stress in cancer cells leading finally to their disfunction and death is currently a hot topic in cancer research. In this work, we developed novel ER-inducing compounds by using the indolo[3,2-*d*][2]benzazepine backbone, whose structure is related to indolo[3,2-*c*]quinoline, paullone, and latonduine scaffolds. It was shown that the new compounds **HL**^**1**^**–HL**^**4**^ and complexes **1–4** showed high antiproliferative activity in breast cancer and hepatocellular cancer cell lines MDA-MB-231 and LM3, respectively, with IC_50_ values from 0.07 to 1.8 μM. The antiproliferative activity of lead drug candidate **4** in the two mentioned cancer cell lines (0.22 ± 0.04 and 0.11 ± 0.02 μM, respectively) was superior to that of paullone-derived complex **7** (0.45 ± 0.01 and 0.41 ± 0.01 μM) and inferior to that of latonduine-derived compound **11** (0.11 ± 0.02 and 0.08 ± 0.02 μM), even though the cytotoxicity of **4**, **7**, and **11** as representatives of three related series of compounds should be considered as excellent. Taken together, the flipping of indole moiety enhanced the cytotoxicity, while the change of the position of the lactam group had no significant effect on antiproliferative activity. Western blot analysis of ER stress biomarkers PERK, BiP, calnexin, and Ero-1-Lα revealed ER stress activation which finally led to breast cancer cell death. The ability of **4** to produce ROS in the presence of hydrogen peroxide was confirmed by an independent EPR spin-trapping experiment, as well as fluorescent microscopy. This work provides a new platform for further development of ER-inducing metal-based anticancer drugs.

## Experimental Section

2-Iodobenzonitrile, indole-3-carboxaldehyde, and 5-bromoindole-3-carboxaldehyde were purchased from ABCR. Borane solution (1 M in THF), absolute DMF, 4-dimethylaminopyridine (DMAP), di-*tert*-butyl-dicarbonate (Boc_2_O), absolute acetonitrile (ACN), palladium(II) acetate, sodium bicarbonate, tetrabutylammoinium fluoride (TBAF), basic aluminium oxide, and 2-formylpyridine were bought from Fisher/Acros Organics. 2-Acetylpyridine was obtained from TCI. Sodium hydride (NaH, 60% dispersion in mineral oil), copper(II) chloride dihydrate, *p*-toluenesulfonyl chloride, phosphorus pentasulfide, methanol, DCM, THF, ethyl acetate (EtOAc), hexane, celite, and hydrazine monohydrate were purchased from Sigma-Aldrich. EDCI·HCl was obtained from IRIS biotech, while silver(I) carbonate was purchased from Merck. 2-Iodobenzylamine was prepared by a known method.^65^ The proligands **HL**^**5**^–**HL**^**11**^ and the corresponding complexes **5–11** were prepared as reported previously,^33,39^ while 5,12-dihydroindolo[3,2-*d*]benzazepin-7(6*H*)-one was synthesized by following a literature protocol.^37^ Absolute hydrazine was preabsoluted over sodium hydroxide.

### Synthesis of 11-Bromo-5,12-dihydroindolo[3,2-*d*]benzazepin-7(6*H*)-one (**B**)

#### 5-Bromo-1-tosyl-1*H*-indole-3-carboxaldehyde (**b**)^66^

Under argon-flush, 5-bromoindole-3-carboxaldehyde (6.0 g, 26.8 mmol) was dissolved in THF (150 mL). The solution was cooled to 0 °C. Et_3_N (11.16 mL, 80 mmol) and TsCl (8.4 g, 44.0 mmol) were added. The suspension was stirred at room temperature for 12 h. The solvent was removed under reduced pressure, and the residue was afterward recrystallized in methanol. The product was obtained as a white solid. Yield: 9.33 mg, 92%. ^1^H NMR (500 MHz, DMSO-*d*_6_): δ, ppm: 10.05 (s, 1H, CHO), 8.94 (s, 1H, H_Ar_), 8.23 (d, *J* = 2.0 Hz, 1H, H_Ar_), 8.01 (d, *J* = 8.5 Hz, 2H, H_Ar_), 7.94 (d, *J* = 8.9 Hz, 1H, H_Ar_), 7.62 (dd, *J* = 8.9, 2.0 Hz, 1H, H_Ar_), 7.47 (d, *J* = 8.1 Hz, 2H, H_Ar_), 2.35 (s, 3H, CH_3_).

#### 5-Bromo-1-tosyl-1*H*-indole-3-carboxylic Acid (**c**)

5-Bromo-1-tosyl-1*H*-indole-3-carboxaldehyde (**b**) (5.5 g, 14.55 mmol) was dissolved in THF (100 mL). A solution of NaClO_2_ (2.7 g, 70.54 mmol) in water (100 mL) was added followed by sulfamic acid (7.7 g, 79.3 mmol). The mixture was stirred at room temperature for 30 min. Then, a saturated solution of NaHCO_3_ was added to reach pH = 8. Then, THF was removed under reduced pressure, and the aqueous solution was acidified with 6 M HCl to generate the formation of a white precipitate. This was extracted with EtOAc (3× 80 mL). The combined organic phases were dried over magnesium sulfate and concentrated in vacuo. The crude product was recrystallized in methanol to give a white powder. Yield: 4.25 g, 74%. ^1^H NMR (500 MHz, DMSO-*d*_6_): δ, ppm: 13.18 (br s, 1H, COOH), 8.40 (s, 1H, H_Ar_), 8.16 (d, *J* = 1.9 Hz, 1H, H_Ar_), 8.03 (d, *J* = 8.5 Hz, 2H, H_Ar_), 7.93 (d, *J* = 8.9 Hz, 1H, H_Ar_), 7.57 (dd, *J* = 8.9, 2.0 Hz, 1H, H_Ar_), 7.44 (d, *J* = 8.1 Hz, 2H, H_Ar_), 2.34 (s, 3H, CH_3_). ESI-MS (acetonitrile/methanol + 1% water), negative: *m*/*z* 391.84 [M – H]^−^.

#### 5-Bromo-*N*-(2-iodobenzyl)-1-tosyl-1*H*-indole-2-carboxamide (**d**)

Under argon-flush, to a solution of 2-iodobenzylamine (4.25 g, 10.81 mmol) in DCM (100 mL) cooled to 0 °C, 5-bromo-1-tosyl-1*H*-indole-3-carboxylic acid (**c**) (2.77 g, 11.89 mmol) was added, followed by EDCI·HCl (2.27 g, 11.82 mmol) and DMAP (1.32 g, 10.81 mmol). Then, this reaction mixture was stirred at 0 °C for 4 h and at room temperature for 20 h. Water (60 mL) was added. The solution was acidified with 6 M HCl to pH = 1, and the crude product was extracted with DCM (3× 50 mL). The combined organic phases were dried over magnesium sulfate and concentrated in vacuo. The product was washed with ice-cold diethyl ether to give a white solid. Yield: 4.16 g, 64%. ^1^H NMR (500 MHz, DMSO-*d*_6_): δ, ppm: 9.01 (t, *J* = 5.6 Hz, 1H, NH), 8.72 (s, 1H, H_Ar_), 8.31 (d, *J* = 2.0 Hz, 1H, H_Ar_), 7.93 (d, *J* = 8.4 Hz, 2H, H_Ar_), 7.89 (d, *J* = 8.7 Hz, 2H, H_Ar_), 7.55 (dd, *J* = 8.9, 2.1 Hz, 1H, H_Ar_), 7.45 (d, *J* = 8.2 Hz, 2H, H_Ar_), 7.43–7.36 (m, 2H, H_Ar_), 7.09–7.03 (m, 1H, H_Ar_), 4.44 (d, *J* = 5.6 Hz, 2H, CH_2_), 2.35 (s, 3H, CH_3_).

#### *tert*-Butyl (5-Bromo-2-iodobenzyl)(1-tosyl-1*H*-indole-3-carbonyl)carbamate (**e**)

Under argon-flush, to a solution of 5-bromo-*N*-(2-iodobenzyl)-1-tosyl-1*H*-indole-2-carboxamide (**d**) (4.16 g, 6.83 mmol) in ACN (110 mL), Boc_2_O (2.38 g, 10.91 mmol) and a catalytic amount of DMAP were added. The yellow solution was stirred at room temperature overnight. The solvent was evaporated under reduced pressure, and the residue was taken up in EtOAc (80 mL) and washed with water (80 mL). The aqueous phase was extracted with EtOAc (3× 100 mL). The combined organic phases were dried over MgSO_4_ and the solvent was removed on a rotary evaporator. The raw product was purified on a silica column by using EtOAc/hexane 1:3 as eluent to give a yellow oil. Yield: 4.36 mg, 90%. ^1^H NMR (500 MHz, DMSO-*d*_6_): δ, ppm: 8.47 (s, 1H, H_Ar_), 8.04 (d, *J* = 8.4 Hz, 2H, H_Ar_), 8.00 (d, *J* = 8.9 Hz, 1H, H_Ar_), 7.91 (dd, *J* = 7.8, 0.9 Hz, 1H, H_Ar_), 7.87 (d, *J* = 1.8 Hz, 1H, H_Ar_), 7.59 (dd, *J* = 8.9, 1.9 Hz, 1H, H_Ar_), 7.46 (d, *J* = 8.2 Hz, 2H, H_Ar_), 7.42 (t, *J* = 7.5 Hz, 1H, H_Ar_), 7.13 (d, *J* = 7.7 Hz, 1H, H_Ar_), 7.06 (t, *J* = 7.6 Hz, 1H, H_Ar_), 4.84 (s, 2H, CH_2_), 2.35 (s, 3H, CH_3_), 0.93 (s, 9H, 3CH_3_).

#### *tert*-Butyl 9-Bromo-7-oxo-12-tosyl-7,12-dihydrobenzo[5,6]azepino[3,4-*b*]indole-6(5*H*)-carboxylate (**f**)

Under argon-flush, to a solution of *tert*-butyl (5-bromo-2-iodobenzyl)(1-tosyl-1*H*-indole-3-carbonyl)carbamate (**e**) (4.25 g, 6.0 mmol) in absolute DMF (200 mL) were added palladium(II) acetate (0.63 g, 3.0 mmol), triphenylphosphine (0.787 g, 3.0 mmol), and silver(I) carbonate (4.13 g, 15.0 mmol) and stirred at 75 °C for 2.5 h. DMF was removed in vacuo, and the black residue was taken up in DCM. The suspension was filtered through celite and rinsed with DCM. The product was purified on silica by using EtOAc/hexane 1:3 as the eluent and isolated as a white solid. Yield: 2.5 g, 72%. ^1^H NMR (500 MHz, DMSO-*d*_6_): δ, ppm: 8.18–8.13 (m, 2H, *H*_Ar_), 7.90 (d, *J* = 7.9 Hz, 1H, H_Ar_), 7.68 (dd, *J* = 8.8, 2.2 Hz, 1H, H_Ar_), 7.65–7.61 (m, 1H, H_Ar_), 7.58 (m, 2H, H_Ar_), 7.23 (d, *J* = 8.2 Hz, 2H, H_Ar_), 7.13 (d, *J* = 8.4 Hz, 2H, H_Ar_), 5.12 (d, *J* = 15.0 Hz, 1H, CH_2_), 3.98 (d, *J* = 15.0 Hz, 1H, CH_2_), 2.27 (s, 3H, CH_3_), 1.45 (s, 9H, 3CH_3_).

#### 9-Bromo-5,12-dihydrobenzo[5,6]azepino[4,3-*b*]indol-7(6*H*)-one (**B**)

To a solution of *tert*-butyl 9-bromo-7-oxo-12-tosyl-7,12-dihydrobenzo[5,6]azepino[3,4-*b*]indole-6(5*H*)-carboxylate (**f**) (1.7 g, 2.93 mmol) in DCM (77 mL), trifluoroacetic acid (14.3 mL) was added, and the mixture was stirred at room temperature for 2 h. Then, water (110 mL) was added, and the intermediate species was extracted with DCM (3× 100 mL). The combined organic phases were concentrated in vacuo to afford a crude solid, which was used in the next step without further purification. The crude solid was dissolved in absolute THF (51.1 mL), and TBAF (20.4 mL) was added. The reaction mixture was stirred for 30 min and concentrated in vacuo, and the product was purified by column chromatography (MeOH/DCM 2:98) and, finally, crystallized in MeOH to give a white solid. Yield: 0.52 g, 54%. ^1^H NMR (500 MHz, DMSO-*d*_6_): δ, ppm: 12.29 (s, 1H, NH), 8.20 (d, *J* = 2.0 Hz, 1H, H_Ar_), 7.95 (t, *J* = 5.3 Hz, 1H, NH), 7.84 (d, *J* = 7.6 Hz, 1H, H_Ar_), 7.58–7.53 (m, 1H, H_Ar_), 7.50–7.45 (m, 3H, H_Ar_), 7.37 (dd, *J* = 8.6, 2.0 Hz, 1H, H_Ar_), 4.10 (d, *J* = 5.3 Hz, 2H, CH_2_). ESI-MS (ACN/MeOH + 1% water), positive: *m*/*z* 327.13 [M + H]^+^.

### Synthesis of Proligands

#### 5,12-Dihydroindolo[3,2-*d*]benzazepin-7(6*H*)-thione (**C**)

To a solution of 5,12-dihydroindolo[3,2-*d*]benzazepin-7(6*H*)-one (**A**) (1.2 g, 4.84 mmol) in absolute THF (80 mL) in a Schlenk tube under argon atmosphere, a mixture of phosphorus pentasulfide and basic aluminium oxide (0.6:1 w/w) (3.48 g) was added, and the reaction mixture was stirred at 75 °C overnight. The next day, the mixture was cooled to room temperature and filtered. The filtrate was concentrated in vacuo and purified by column chromatography by using MeOH/DCM 1:99 as the eluent. (The starting material was also eluted by using MeOH/DCM 5:95.) The reaction was repeated several times using the recovered starting material with about 15% conversion per circle. The product was obtained as a yellow powder. Yield: 766 mg, 60%. ^1^H NMR (500 MHz, DMSO-*d*_6_): δ, ppm: 12.28 (s, 1H, NH), 10.00 (t, *J* = 5.7 Hz, 1H, NH), 8.61 (d, *J* = 8.1 Hz, 1H, H_Ar_), 7.91–7.85 (m, 1H, H_Ar_), 7.59 (td, *J* = 7.5, 1.5 Hz, 1H, H_Ar_), 7.54 (td, *J* = 7.4, 1.3 Hz, 1H, H_Ar_), 7.50 (m, 2H, H_Ar_), 7.31–7.26 (m, 1H, H_Ar_), 7.21–7.16 (m, 1H, H_Ar_), 4.18 (br d, *J* = 91.9 Hz, 2H, CH_2_).

#### 11-Bromo-5,12-dihydroindolo[3,2-*d*]benzazepin-7(6*H*)-thione (**D**)

To a solution of 5,12-dihydroindolo[3,2-*d*]benzazepin-7(6*H*)-one (**B**) (0.54 g, 1.65 mmol) in absolute THF (26 mL) in a Schlenk tube under argon atmosphere, a mixture of phosphorus pentasulfide and basic aluminium oxide (0.6:1 w/w) (1.17 g) was added, and the reaction mixture was stirred at 75 °C overnight. The next day, the mixture was cooled to room temperature and filtered. The filtrate was concentrated in vacuo and purified by column chromatography by using MeOH/DCM 1:99 as the eluent. (The starting material was also eluted with MeOH/DCM 5:95.) The reaction was repeated several times using the recovered starting material with about 15% conversion per circle. The product was obtained as a yellow powder. Yield: 766 mg, 60%. ^1^H NMR (500 MHz, DMSO-*d*_6_): δ, ppm: 12.50 (s, 1H, NH), 10.11 (t, *J* = 5.5 Hz, 1H, NH), 8.79 (d, *J* = 2.0 Hz, 1H, H_Ar_), 7.89–7.86 (m, 1H, H_Ar_), 7.62–7.54 (m, 2H, H_Ar_), 7.52–7.49 (m, 1H, H_Ar_), 7.48 (d, *J* = 8.6 Hz, 1H, H_Ar_), 7.41 (dd, *J* = 8.6, 2.0 Hz, 1H, H_Ar_), 4.19 (d, *J* = 54.0 Hz, 2H, CH_2_).

#### 7-Hydrazin-yl-5,12-dihydroindolo[3,2-*d*]benzazepin-(6*H*)-one (**E**)

A suspension of 5,12-dihydroindolo[3,2-*d*]benzazepin-7(6*H*)-thione (**C**) (500 mg, 2.08 mmol) in freshly distilled hydrazine (15 mL) was refluxed under argon atmosphere at 135 °C overnight. The reaction mixture was cooled to room temperature, and water (15 mL) was added. The white precipitate was filtered off, washed with water, and dried in vacuo. Yield: 200 mg, 37%. ^1^H NMR (500 MHz, DMSO-*d*_6_): δ, ppm: 11.64 (br s, 1H, NH), 8.12 (d, *J* = 8.5 Hz, 1H, H_Ar_), 7.76 (d, *J* = 7.6 Hz, 1H, H_Ar_), 7.48 (t, *J* = 6.9 Hz, 1H, H_Ar_), 7.39 (m, 3H, H_Ar_), 7.17 (t, *J* = 7.6 Hz, 1H, H_Ar_), 7.05 (t, *J* = 7.4 Hz, 1H, H_Ar_), 6.25 (br s, 1H, NH), 4.71 (br s, 2H, NH_2_), 4.10 (s, 2H, CH_2_). ESI-MS (ACN/MeOH + 1% water), positive: *m*/*z* 263.08 [M + H]^+^.

#### 11-Bromo-7-hydrazin-yl-5,12-dihydroindolo[3,2-*d*][2]benzazepin-(6*H*)-one (**F**)

A suspension of 11-bromo-5,12-dihydroindolo[3,2-*d*]benzazepin-7(6*H*)-thione (**D**) (400 mg, 1.17 mmol) in freshly distilled hydrazine (10 mL) was refluxed under argon atmosphere at 135 °C overnight. The reaction mixture was cooled to room temperature, and water (6 mL) was added. The white precipitate was filtered off and dried in vacuo. Yield: 316 mg, 79%. ^1^H NMR (500 MHz, DMSO-*d*_6_): δ, ppm: 11.84 (br s, 1H), 8.32 (s, 1H), 7.77 (d, *J* = 7.6 Hz, 1H), 7.50–7.46 (m, 1H), 7.41–7.36 (m,3H), 7.28 (dd, *J* = 8.6, 1.9 Hz, 1H), 6.23 (br s, 1H), 4.77 (br s, 1H), 4.10 (s, 2H, CH_2_). ESI-MS (acetonitrile/methanol + 1% water), positive: *m*/*z* 342.17 [M + H]^+^.

#### **HL**^**1**^·0.6H_2_O

A solution of 7-hydrazin-yl-5,12-dihydroindolo[3,2-*d*]benzazepin-(6*H*)-one (**E**) (200 mg, 0.76 mmol) in MeOH (5.7 mL) in a 25 mL Schlenk tube was deoxygenated by bubbling argon through the solution for 10 min. 2-Formylpyridine (79 μL, 1 equiv) was added, and the mixture was stirred at 75 °C overnight. The reaction mixture was cooled to room temperature, and the solvent was evaporated under reduced pressure. The yellow product precipitated by the addition of Et_2_O (6 mL) was filtered off. Yield: 242 mg, 91%. Anal. Calcd for C_22_H_17_N_5_·0.6H_2_O (*M*_r_ 361.95): C, 72.94; H, 5.07; N, 19.34. Found: C, 73.21; H, 4.86; N, 18.83. ^1^H NMR (600 MHz, DMSO-*d*_6_): δ, ppm: 12.11 (s, 1H, H^12^), 8.57 (d, *J* = 4.4 Hz, 1H, H^18^), 8.38–8.28 (m, 3H, H^8^, H^15^, H^21^), 8.13 (m, 1H, H^6^), 7.87 (d, *J* = 7.7 Hz, 1H, H^1^), 7.83 (dd, *J* = 11.0, 4.2 Hz, 1H, H^20^), 7.55 (m, 1H, H^2^), 7.52 (d, *J* = 8.1 Hz, 1H, H^11^), 7.48 (m, 2H, H^3^, H^4^), 7.33 (dd, *J* = 6.5, 5.1 Hz, 1H, H^19^), 7.26 (t, *J* = 7.2 Hz, 1H, H^10^), 7.17 (t, *J* = 7.4 Hz, 1H, H^9^), 4.27 (s, 2H, H^5^). ^13^C{H} NMR (176 MHz, DMSO-*d*_6_): δ, ppm: 159.16 (Cq, C^7^), 154.97 (Cq, C^16^), 150.12 (CH,C^15^), 149.22 (CH, C^18^), 138.93 (Cq, C^4a^), 138.81 (Cq, C^12a^), 136.60 (Cq, C^11a^), 136.12 (CH, C^20^), 130.88 (Cq, C^12b^), 128.82 (CH, C^3^), 128.00 (CH, C^4^), 127.99 (CH, C^2^), 126.84 (Cq, C^7b^), 126.83 (CH, C^1^), 123.37 (CH, C^19^), 123.15 (CH, C^8^), 123.10 (CH, C^10^), 120.54 (CH, C^21^), 120.32 (CH, C^9^), 111.40 (CH, C^11^), 107.98 (Cq, C^7a^), 45.67 (CH_2_, C^5^). ESI-MS (ACN/MeOH + 1% water), positive: *m*/*z* 352.25 [M + H]^+^.

#### **HL**^**2**^·0.8H_2_O

A solution of 11-bromo-7-hydrazin-yl-5,12-dihydroindolo[3,2-*d*]benzazepin(6*H*)one (**F**) (150 mg, 0.44 mmol) in MeOH (6.3 mL) in a 25 mL Schlenk tube was deoxygenated by bubbling argon through the solution for 10 min. 2-Formylpyridine (52.9 μL, 1 equiv) was added, and the mixture was stirred at 75 °C overnight. The reaction mixture was cooled to room temperature, and the solvent was removed under reduced pressure. The yellow product precipitated by addition of Et_2_O (6 mL) was filtered off. Yield: 135 mg, 72%. Anal. Calcd for C_22_H_16_BrN_5_·0.8H_2_O (*M*_r_ 443.47): C, 60.06; H, 4.24; N, 15.36. Found: C, 60.10; H, 3.96; N, 15.23. ^1^H NMR (600 MHz, DMSO-*d*_6_): δ, ppm: 12.32 (s, 1H, H^12^), 8.58 (d, *J* = 4.5 Hz, 1H, H^18^), 8.46 (d, *J* = 1.8 Hz, 1H, H^8^), 8.36–8.28 (m, 2H, H^15,21^), 8.15 (t, *J* = 5.2 Hz, 1H, H^6^), 7.87 (d, *J* = 7.7 Hz, 1H, H^1^), 7.83 (dd, *J* = 11.7, 4.9 Hz, 1H, H^20^), 7.59–7.53 (m, 1H, H^2^), 7.49 (m, 3H, H^3,4,11^), 7.42–7.36 (m, 1H, H^10^), 7.34 (dd, *J* = 6.5, 5.0 Hz, 1H, H^19^), 4.27 (d, *J* = 4.1 Hz, 2H, H^5^). ^13^C{H} NMR (176 MHz, DMSO-*d*_6_): δ, ppm: 158.82 (Cq, C^7^), 154.82 (Cq, C^16^), 150.53 (CH, C^15^), 149.26 (CH, C^18^), 140.05 (Cq, C^4a^), 139.07 (Cq, C^12a^), 136.19 (CH, C^20^), 135.35 (Cq, C^11a^), 130.44 (Cq, C^12b^), 129.27 (CH, C^3^), 128.50 (Cq, C^7b^), 128.13 (CH, C^4^), 128.12 (CH, C^2^), 126.97 (CH, C^1^), 125.64 (CH, C^10^), 125.10 (CH, C^8^), 123.51 (CH, C^19^), 120.65 (CH, C^21^), 113.51 (CH, C^11^), 112.93 (Cq, C^9^), 107.44 (Cq, C^7a^), 45.62 (CH_2_, C^5^). ESI-MS (acetonitrile/methanol + 1% water), positive: *m*/*z* 432.18 [M + H]^+^.

#### **HL**^**3**^·0.8H_2_O

A solution of 7-hydrazin-yl-5,12-dihydroindolo[3,2-*d*]benzazepin(6*H*)one (**E**) (200 mg, 0.76 mmol) in methanol (5.7 mL) in a 25 mL Schlenk tube was deoxygenated by bubbling argon through the solution for 10 min. 2-Acetylpyridine (94 μL, 1.1 equiv) was added, and the mixture was stirred at 75 °C overnight. The reaction mixture was cooled to room temperature, and the solvent was removed under reduced pressure. The yellow product was precipitated by addition of diethyl ether (6 mL) and filtered off. Yield: 200 mg, 72%. Anal. Calcd for C_23_H_19_N_5_·0.8H_2_O (*M*_r_ 379.57): C, 72.73; H, 5.46; N, 18.43. Found: C, 72.75; H, 5.08; N, 18.36. ^1^H NMR (600 MHz, DMSO-*d*_6_) δ, ppm: 12.06 (s, 1H, H^12^), 8.57 (d, *J* = 4.3 Hz, 1H, H^18^), 8.49 (d, *J* = 8.0 Hz, 1H, H^21^), 8.39 (d, *J* = 8.0 Hz, 1H, H^8^), 7.93 (t, *J* = 5.0 Hz, 1H, H^6^), 7.87 (d, *J* = 7.7 Hz, 1H, H^1^), 7.82–7.75 (m, 1H, H^20^), 7.58–7.50 (m, 2H, H^2^, H^11^), 7.47 (d, *J* = 4.0 Hz, 2H, H^4^, H^3^), 7.33 (dd, *J* = 6.5, 5.2 Hz, 1H, H^19^), 7.26 (t, *J* = 7.3 Hz, 1H, H^10^), 7.17 (t, *J* = 7.4 Hz, 1H. H^9^), 4.28 (d, *J* = 3.0 Hz, 2H, H^5^) 2.50 (s, 3H, H^22^). ^13^C{H} NMR (176 MHz, DMSO-*d*_6_) δ, ppm: 157.37 (Cq, C^7^), 156.81 (Cq, C^16^), 155.82 (Cq, C^15^), 148.36 (CH, C^18^), 138.98 (Cq, C^4a^), 138.41 (Cq, C^12a^), 136.66 (Cq, C^11a^), 135.75 (CH, C^20^), 131.03 (Cq, C^12b^), 128.68 (Cq, C^3^), 127.96 (CH, C^4^), 127.95 (CH, C^2^), 127.05 (Cq, C^7b^), 126.77 (CH, C^1^), 123.06 (CH, C^19^), 123.03 (CH, C^10^), 123.00 (CH, C^8^), 120.60 (CH, C^21^), 120.33 (CH, C^9^), 111.41 (CH, C^11^), 108.85 (Cq, C^7a^), 45.68 (CH_2_, C^5^), 12.94 (CH_3_, C^22^). ESI-MS (ACN/MeOH + 1% water), positive: *m*/*z* 366.29 [M + H]^+^.

#### **HL**^**4**^·0.5H_2_O

A solution of 11-bromo-7-hydrazin-yl-5,12-dihydroindolo[3,2-*d*]benzazepin(6*H*)one (**F**) (150 mg, 0.43 mmol) in methanol (6.5 mL) in a 10 mL Schlenk tube was deoxygenated by bubbling argon through the solution for 10 min. 2-Acetylpyridine (48.2 μL, 1 equiv) was added, and the mixture was stirred at 75 °C overnight. The reaction mixture was cooled to room temperature, and the solvent was removed under reduced pressure. The yellow product was precipitated by addition of diethyl ether (6.5 mL) and filtered off. Yield: 125 mg, 60%. Anal. Calcd for C_23_H_18_BrN_5_·0.5H_2_O (*M*_r_ 452.08): C, 61.05; H, 4.23; N, 15.49. Found: C, 61.26; H, 4.18; N, 14.95. IR spectrum (selected bands, ATR, ν_max_, cm^–1^): 1593 (s), 1536 (s), 1470 (s), 1432 (s), 1294 (m), 1253 (m), 1160 (m), 992 (m), 791 (w), 651 (s). ^1^H NMR (600 MHz, DMSO-*d*_6_): δ, ppm: 12.27 (s, 1H, H^12^), 8.58 (d, *J* = 4.1 Hz, 1H, H^18^), 8.56 (d, *J* = 1.9 Hz, 1H, H^8^), 8.49 (d, *J* = 8.0 Hz, 1H, H^21^), 7.94 (t, *J* = 5.2 Hz, 1H, H^6^), 7.86 (d, *J* = 7.6 Hz, 1H, H^1^), 7.79 (td, *J* = 7.9, 1.7 Hz, 1H, H^20^), 7.58–7.53 (m, 1H, H^4^), 7.51–7.45 (m, 3H, H^2,3,11^), 7.39 (dd, *J* = 8.6, 2.0 Hz, 1H, H^10^), 7.36–7.32 (m, 1H, H^19^), 4.28 (d, *J* = 4.6 Hz, 2H, H^5^), 2.49 (s, 3H, H^22^). ^13^C{H} NMR (176 MHz, DMSO-*d*_6_) δ, ppm: 157.05 (Cq, C^7^), 156.69 (Cq, C^16^), 156.14 (Cq, C^15^), 148.40 (CH, C^18^), 139.68 (Cq, C^4a^), 139.07 (Cq, C^12a^), 135.81 (CH, C^20^), 135.37 (Cq, C^11a^), 130.56 (Cq, C^12b^), 129.15 (CH, C^3^), 128.70 (Cq, C^7b^), 128.13 (CH, C^4^), 128.07 (CH, C^2^), 126.91 (CH, C^1^), 125.53 (CH, C^10^), 125.19 (CH, C^8^), 123.20 (CH, C^19^), 120.69 (CH, C^21^), 113.47 (CH, C^11^), 112.91 (Cq, C^9^), 108.31 (Cq, C^7a^), 45.66 (CH_2_, C^5^), 12.83 (CH_3_, C^22^). ESI-MS (acetonitrile/methanol + 1% water), positive: *m*/*z* 444.20 [M + H]^+^.

### Synthesis of Copper(II) Complexes

#### [CuCl(**HL**^**1**^)]Cl·H_2_O (1·H_**2**_O)

To a solution of **HL**^**1**^ (125 mg, 0.35 mmol) in MeOH (40 mL), a solution of CuCl_2_·2H_2_O (59 mg, 0.35 mmol) in MeOH (2 mL) was added. The reaction mixture was refluxed for 30 min, cooled down and allowed to stand at 4 °C overnight. The product was filtered off and dried in vacuo to give a brown powder. Yield: 152.5 mg, 91%. Anal. Calcd for C_22_H_17_Cl_2_CuN_5_·H_2_O (*M*_r_ 502.03): C, 52.44; H, 3.80; N, 13.90. Found: C, 52.13; H, 3.79; N, 13.61. IR spectrum (selected bands, ATR, ν_max_, cm^–1^): 1592 (w), 1499 (m), 1439 (m), 1336 (w), 1292 (w), 1219 (m), 1160 (m), 1122 (m), 1022 (w), 749 (s), 652 (s). Solubility in water/1% DMSO ≥ 1.0 mg mL^–1^. ESI-MS (ACN/MeOH + 1% water), positive: *m*/*z* 449.17 [Cu^II^Cl(HL^1^)]^+^, 863.16 {[Cu^II^Cl(L^1^)][Cu^II^(L^1^)]}^+^.

#### [CuCl(**HL**^**2**^)]Cl (2·2H_2_O)

To a solution of **HL**^**2**^ (65.5 mg, 0.15 mmol) in methanol (20 mL), a solution of CuCl_2_·2H_2_O (26 mg, 0.15 mmol) in methanol (2 mL) was added. The reaction mixture was refluxed for 30 min, cooled down, and allowed to stand at 4 °C overnight. Green crystals were filtered off and dried in vacuo. Yield: 66.5 mg, 77%. Anal. Calcd for C_22_H_16_BrCl_2_CuN_5_·2H_2_O (*M*_r_ 597.95): C, 44.15; H, 3.37; N, 11.71. Found: C, 43.97; H, 3.50; N, 11.34. IR spectrum (selected bands, ATR, ν_max_, cm^–1^): 1582 (m), 1535 (w), 1499 (s), 1422 (vs), 1337 (w), 1296 (m), 1214 (s), 1158 (s), 1124 (m), 747 (s), 709 (m), 670 (m), 644 (w). Solubility in water/1% DMSO ≥ 1.0 mg mL^–1^. ESI-MS (ACN/MeOH + 1% water), positive: *m*/*z* 528.95 [Cu^II^Cl(HL^2^)]^+^. HRMS (ESI): *m*/*z* [Cu^II^Cl(HL^2^)]^+^ calcd for C_22_H_16_BrClCuN_5_ 528.9548; found 528.9539.

#### [CuCl(**HL**^**3**^)(MeOH)]Cl·2H_2_O (3·2H_2_O)

To a solution of **HL**^**3**^ (126 mg, 0.35 mmol) in methanol (40 mL), a solution of CuCl_2_·2H_2_O (59 mg, 0.35 mmol) in methanol (2 mL) was added. The reaction mixture was refluxed for 30 min, cooled down, and allowed to stand at 4 °C overnight. Green crystals were filtered off and dried in vacuo. Yield: 140 mg, 81%. Anal. Calcd for C_24_H_23_Cl_2_CuN_5_O·2H_2_O (*M*_r_ 566.08): C, 50.75; H, 4.79; N, 12.33. Found: C, 50.88; H, 4.81; N, 12.37. IR spectrum (selected bands, ATR, ν_max_, cm^–1^): 1591 (m), 1493 (s), 1435 (vs), 1329 (m), 1185 (s), 1102 (m), 1020 (w), 953 (w), 752 (vs), 670 (w), 640 (w). Solubility in water/1% DMSO ≥ 1.0 mg mL^–1^. HRMS (ESI), positive: *m*/*z* 427.0856 calcd for [Cu^II^(L^3^)]^+^ (C_23_H_18_CuN_5_), 427.0853.

#### [CuCl(**HL**^**4**^)]Cl·1.5H_2_O (4·1.5H_2_O)

To a solution of **HL**^**4**^ (110 mg, 0.25 mmol) in methanol (73 mL), a solution of CuCl_2_·2H_2_O (42 mg, 0.25 mmol) in methanol (2 mL) was added. The reaction mixture was refluxed for 30 min, cooled down, and allowed to stand at 4 °C overnight. The product was filtered off and dried in vacuo to give a green powder. Yield: 134 mg, 93%. Anal. Calcd for C_23_H_18_BrCl_2_CuN_5_·1.5H_2_O (*M*_r_ 602.96): C, 45.77; H, 3.51; N, 11.61. Found: C, 45.73; H, 3.36; N, 11.71. IR spectrum (selected bands, ATR, ν_max_, cm^–1^): 1597 (s), 1537 (w), 1504 (m), 1462 (s), 1433 (vs), 1378 (w), 1297 (m), 1199 (s), 1106 (m), 1025 (w), 959 (w), 923 (w), 861 (w), 777 (m), 747 (s), 716 (m), 640 (w). Solubility in water/1% DMSO ≥ 1.0 mg mL^–1^. HRMS (ESI) positive: *m*/*z* 542.9702 calcd for [Cu^II^Cl(HL^4^)]^+^ (C_23_H_18_BrClCuN_5_), 542.9705.

Electrospray ionization (ESI) mass spectra were recorded on a Bruker amaZon SL ion trap spectrometer or a Bruker maXis UHR-TOF mass spectrometer in the positive mode by direct infusion at the Mass Spectrometry Centre of the University of Vienna. One- and two-dimensional ^1^H and ^13^C NMR spectra were recorded on a Bruker AV Neo 500 or AV III 600 spectrometer at 25 °C. For ^1^H and ^13^C NMR spectra, the solvent residual peak was taken as the internal reference. UV–vis spectra were recorded on an Agilent 8453 UV–visible spectroscopy system at 25 °C. Elemental analysis measurements were done on a PerkinElmer 2400 CHN elemental analyzer at the Microanalytical Laboratory of the University of Vienna and are within ±0.4%, confirming >95% purity of the compounds. The infrared spectra were recorded by a Bruker Vertex 70 spectrometer, and the spectra were treated by extended ATR correction.

### Additional Determination of Purity of Complex **4**

Reverse-phase HPLC analysis of compound **4** was performed on a system composed of a maXis UHR ESI-Qq-TOF mass spectrometer (Bruker Daltonics, Bremen, Germany) coupled to a HPLC-system (UltiMate 3000, Dionex). Separation was carried out on a C18 analytical column AcclaimTM 120 (Thermo Scientific, 2.1 × 150 mm, 3 μm, 120 Å) at a flow rate of 0.3 mL/min. Column temperature: 25 °C. Mobile phase A: (100% MeOH + 0.1% FA); mobile phase B: (100% ACN + 0.1% FA). UV: 254, 280, and 350 nm. The sum formulas of the detected ions were determined using Bruker Compass DataAnalysis 5.1 based on the mass accuracy (Δ*m*/*z* ≤ 5 ppm) and isotopic pattern matching (SMART Formula algorithm).

### Crystallographic Structure Determination

The measurements were performed on Bruker X8 APEXII CCD and Bruker D8 Venture diffractometers. Single crystals were positioned at 30, 30, 30, and 30 mm from the detector, and 180, 4848, 816, and 287 frames were measured, each for 15, 15, 60, and 3 s over 1, 0.5. 0.5, and 1° scan width for species **f**, [**CuCl**(**HL**^**2**^)]**Cl** (**2**), [**CuCl**(**HL**^**3**^)]**Cl**, and [**CuCl**(**HL**^**3**^)(**MeOH**)]**Cl**·**MeOH** (**3**·**MeOH**), respectively. The data were processed using SAINT software.^67^ Data collection for [**CuCl**(**L**^**3**^)]**Cl**·**EtOH** was performed on the XRD2 beamline, Sincrotrone Elettra Trieste SCpA. A superconducting wiggler acted as a light source for the beamline, with a dual crystal Si(111) monochromator providing wavelength selection in the 8–30 keV range. The beamline was equipped with an Arinax MD2S high-throughput diffractometer, a Pilatus 6M detector, and an open flow nitrogen cryostat. Data processing and frame integration were performed using the XDS package,^68^ as implemented in the XRD4Elettra interface, and space group confirmation was provided by Pointless from the CCP4 suite. Crystal data, data collection parameters, and structure refinement details are given in Table 2. The structures were solved by direct methods and refined by full-matrix least-squares techniques. Non-H atoms were refined with anisotropic displacement parameters. H atoms were inserted in calculated positions and refined with a riding model. The following computer programs and hardware were used: structure solution, *SHELXS-2014* and refinement, *SHELXL-2014*;^69^ molecular diagrams, ORTEP;^70^ computer, Intel CoreDuo. CCDC 2165424 (**f**), 2165425 (**2**), 2165426 ([**CuCl**(**HL**^**3**^)]**Cl**), 2165427 (**3·MeOH**), and 2165428 ([**CuCl**(**L3**)]·**EtOH**).

**Table 2:** Crystal Data and Details of Data Collection for Species **f**, [**CuCl**(**HL**^**2**^)]**Cl** (**2**), [**CuCl**(**HL**^**3**^)]**Cl**, **3·MeOH**, and [**CuCl**(**L**^**3**^)]·**EtOH**

compound	**f**	**2**	[**CuCl**(**HL**^**3**^)]**Cl**	**3·MeOH**	[**CuCl**(**L**^**3**^)]·**EtOH**
empirical formula	C_24.25_H_23_N_2_O_3.25_S	C_22_H_16_BrCl_2_CuN_5_	C_23_H_19_Cl_2_CuN_5_	C_25_H_27_Cl_2_CuN_5_O_2_	C_25_H_24_ClCuN_5_O N_4_o_2_SCuCl
fw	442.50	564.75	499.87	563.96	509.48
space group	monoclinic, *P*2_1_/*c*	monoclinic, *C*/*c*	orthorhombic, *Pbca*	monoclinic, *P*2_1_/*c*	monoclinic, *P*2_1_/*n*
*a*, Å	9.906(2)	11.8133(15)	13.7377(4)	12.9587(7)	10.344(2)
*b*, Å	10.419(2)	26.403(4)	14.3794(5)	14.2828(8)	14.404(3)
*c*, Å	21.194(5)	7.8396(10)	20.6147(6)	13.6405(5)	15.761(3)
α, °	90				
β, °	96.380(7)	90.584(4)		99.273(3)	108.50(3)
	90				
*V* [Å^3^]	2173.8(8)	2445.1(6)	4072.2(2)	2491.7(2)	2226.9(8)
*Z*	4	4	8	4	4
λ [Å]	0.71073	0.71073	0.71073	0.71073	0.70000
ρ_calcd_, g cm^–3^	1.352	1.534	1.631	1.124	1.520
cryst size, mm^3^	0.10 × 0.10 × 0.04	0.10 × 0.08 × 0.04	0.10 × 0.02 × 0.01	0.18 × 0.15 × 0.10	0.03 × 0.02 × 0.01
*T* [K]	150(2)	100(2)	120(2)	120(2)	100(2)
μ, mm^–1^	0.184	2.764	1.358	1.124	1.087
*R*_1_^a^	0.0432	0.0330	0.0474	0.0326	0.0489
*wR*_2_^b^	0.0966	0.0994	0.1189	0.0857	0.1390
GOF^c^	1.063	1.079	0.998	1.060	1.051

a *R*_1_ = Σ||*F*_o_| – |*F*_c_||/Σ|*F*_o_|.

b *wR*_2_ = {Σ[*w*(*F*_o_^2^ – *F*_c_^2^)^2^]/Σ[*w*(*F*_o_^2^)^2^]}^1/2^.

c GOF = {Σ[*w*(*F*_o_^2^ – *F*_c_^2^)^2^]/(*n* – *p*)}^1/2^, where *n* is the number of reflections and *p* is the total number of parameters refined.

### Biological Investigations

#### Cell Lines and Culture Conditions

Human breast adenocarcinoma MDA-MB-231 and human embryonic kidney HEK293 cells were obtained from ATCC. Hepatocellular carcinoma LM3 cells were a kind gift of Prof. Kan Man Hui (Duke-NUS Medical School). All cells were cultured in DMEM medium containing 10% FBS in tissue culture 75 cm^2^ flasks (BDBiosciences, Singapore) at 37 °C in a humidified atmosphere of 95% air and 5% CO_2_. All stock solutions were prepared in DMSO. The amount of actual Cu concentration in stock solutions was verified by ICP-OES.

#### Inhibition of Cell Viability Assay

The cytotoxicity of the compounds was determined by the colorimetric MTT assay. The cells were harvested from culture flasks by trypsinization and seeded into CELLSTAR 96-well microculture plates at the seeding density of 6000 cells per well (6 × 10^4^ cells/mL). After the cells were allowed to resume exponential growth for 24 h, they were exposed to drugs at different concentrations in media for 72 h. The drugs were diluted in complete medium at the desired concentration and added to each well (100 μL) and serially diluted to other wells. After exposure for 72 h, the media was replaced with MTT in media (5 mg/mL, 100 μL) and incubated for additional 45 min. Subsequently, the medium was aspirated, and the purple formazan crystals formed in viable cells were dissolved in DMSO (100 μL). Optical densities were measured at 570 nm using the BioTekH1 Synergy microplate reader. The quantity of viable cells was expressed in terms of treated/control (T/C) values by comparison to untreated control cells, and 50% inhibitory concentrations (IC_50_) were calculated from concentration–effect curves by interpolation. Evaluation was based on means from at least three independent experiments, each comprising six replicates per concentration level.

#### Western Blotting

MDA-MB-231 cells were seeded into 100 mm dishes at a density of 22 × 10^5^ cells/dish (7 mL per dish). After the cells were allowed to grow for 48 h, they were treated with **4** at different concentrations for 24 h. After treatment, the cells were washed twice with ice-cold PBS and lysed directly on the dish using RIPA buffer [100 μL, 0.1% SDS, 0.5% sodium deoxycholate, 1% IGEPAL CA-630, 150 mM NaCl, 25 mM Tris-HCl (pH 8.0), protease and phosphatase inhibitor cocktail]. The cell lysates were scraped from the dishes and transferred to separate 1.5 mL microtubes. The lysate was incubated with shaking at 4 °C for 10 min and centrifuged at 12,000 × *g* at 4 °C for 20 min. The supernatant was collected, and the protein concentration for each sample was measured using the BCA assay (Thermo Scientific Pierce BCA Protein Assay Kit). Samples with the same protein concentration (40 μg) were reconstituted in loading buffer (100 mM DTT, 1× protein loading dye) and heated at 95 °C for 5 min. The protein mixtures were separated on SDS-PAGE gel (10%) and then transferred to a 0.2 μm nitrocellulose membrane. Afterward, the membrane was blocked in 5% BSA for 1 h and incubated with primary antibodies [CST: calnexin (C5C9) rabbit mAb #2679, PERK (D11A8) rabbit mAb #5683, Ero1-Lα antibody #3264, BiP (C50B12) rabbit mAb #3177] overnight at 4 °C. The samples were subsequently incubated with β-actin antibody [CST: β-Actin (D6A8) rabbit mAb #8457] as a loading control. The membranes were washed four times (5 min each) with TBST and incubated for 2 h with a secondary antibody. After incubation, the membranes were washed again with TBST four times (5 min each) and visualized using an Immobilon Crescendo Western HPR substrate and a chemiluminescence imaging machine (ChemiDoc Touch Imaging System, BioRad). The level of expression for each protein was analyzed using Bio-Rad Image Lab Software.

#### ROS Detection by Fluorescent Microscopy

*Coverslips preparation*: Coverslips were soaked in ethanol (95%, 15 mL) for 15 min, washed with water (Milli-Q, 2× 1 mL), and placed into the wells of a 6-well culture plate containing poly-l-lysine solution (1 mg/mL, 1 mL). Coverslips were left in the diluted poly-l-lysine solution for 1 h at room temperature. Subsequently, the free amino acid solution was removed, and coverslips were washed with water (Milli-Q, 5× 2 mL) and left to dry for 2 h. *Sample preparation*: Poly-l-lysine-coated coverslips were placed into each well of a 6-well culture plate (Greiner Bio-One), and MDA-MB-231 cells were seeded onto poly-l-lysine-coated coverslips at a density of 5.5 × 10^5^ cells per mL (2 mL per well). Cells were allowed to resume exponential growth for 24 h. The cell culture medium was aspirated and washed with PBS (2× 1 mL). In a low light environment, the DCFDA (2′,7′-dichlorodihydrofluorescein diacetate, Sigma-Aldrich) solution prepared in 1× HBSS (20 μM, 1 mL) was added to each well and incubated for 15 min (37 °C, 5% CO_2_). DCFDA solution was aspirated and washed with HBSS (2× 800 μL). The drug solutions at desired concentrations were prepared in colorless cell culture media, and the culture plates were incubated for 4 h (37 °C, 5% CO_2_). After the drug solutions were aspirated and washed with 1× HBSS (2× 1 mL), the coverslips were carefully lifted with a needle and mounted on glass slides with mounting media glycerol/PBS (9:1). The samples were protected from photodegradation by covering them with an aluminium foil before imaging. *Fluorescent microscopy*: Glass slides were inspected using a Nikon inverted microscope Ti-U Imaging System in a dark room via a 10× dry lens objective. Fluorescent images were obtained using a fluorescein isothiocyanate filter with a Nikon monochrome camera Qi1Mc, and bright-field images were obtained using a Nikon color camera Fi3. The images were processed and analyzed using the NIS Elements BR imaging software (version 5.30.03).

#### EPR Spin-Trapping Experiments

The generation of paramagnetic intermediates was monitored by cw-EPR spectroscopy using the Adani spectrometer EPR PS 110.X. The application of EPR spin-trapping experiments involved the spin-trapping agent DMPO (Sigma-Aldrich), which was distilled prior to the use (70 Pa; 80–90 °C). The solution of the spin trap and the studied complex **4** was mixed with hydrogen peroxide to initiate the Fenton-like reaction. EPR spectra were measured 5 and 10 min after the addition of H_2_O_2_. The standard settings during EPR spin trapping experiments were microwave frequency ∼9.5 GHz, power of the microwave radiation ∼25 mW, modulation amplitude 1.5 G, and 10 scans.

## Supporting Information Available

NMR numbering schemes; NMR spectra; HPLC-MS report; X-ray crystallography; and UV–vis spectra (PDF)
