# Supplementary material for: Elucidation of Structure–Activity Relationships in Indolobenzazepine-Derived Ligands and Their Copper(II) Complexes: the Role of Key Structural Components and Insight into the Mechanism of Action
Source: Inorg Chem. 2022 Jun 17;61(26):10167–81. doi: 10.1021/acs.inorgchem.2c01375 (PMC9490829; doi:10.1021/acs.inorgchem.2c01375)
Supplement: Supplementary file 1 — ic2c01375_si_001.pdf [file ic2c01375_si_001.pdf]

Supporting Information

for

Elucidation of structure-activity relationships in indolo[3,2-*d*]benzazepine-derived ligands and their copper(II) complexes: the role of key structural components and insight into the mechanism of action

Irina Kuznetcova,[a] Felix Bacher,[a,≠] Samah Mutasim Alfadul,[b,≠] Max Jing Rui Tham,[c] Wee Han Ang,[c] Maria V. Babak,[b,*] Peter Rapta[d] and Vladimir B. Arion[a,*]

[a]Institute of Inorganic Chemistry of the University of Vienna, Währinger Strasse 42, A-1090 Vienna, Austria

[b]Drug Discovery Lab, Department of Chemistry, City University of Hong Kong, Hong Kong SAR 999077

[c]Department of Chemistry, National University of Singapore, 4 Science Drive 2, 117544, Singapore

[d]Institute of Physical Chemistry and Chemical Physics, Faculty of Chemical and Food Technology, Slovak University of Technology in Bratislava, Radlinského 9, SK-81237 Bratislava, Slovak Republic

≠ equally contributing authors

*corresponding author

Contents

NMR numbering schemes *3*

NMR spectra *3*

High Performance Liquid Chromatography-MS report of 1 .... *26*

X-ray crystallography *31*

UV–vis spectra *32*

NMR numbering schemes

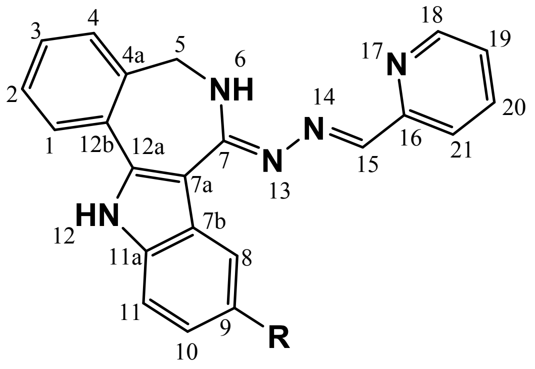

R = H (**HL^1**)

R = Br (**HL^2**)

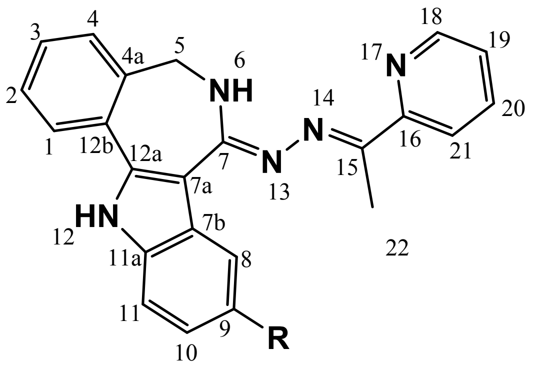

R = H (**HL^3**)

R = Br (**HL^4**)

NMR spectra

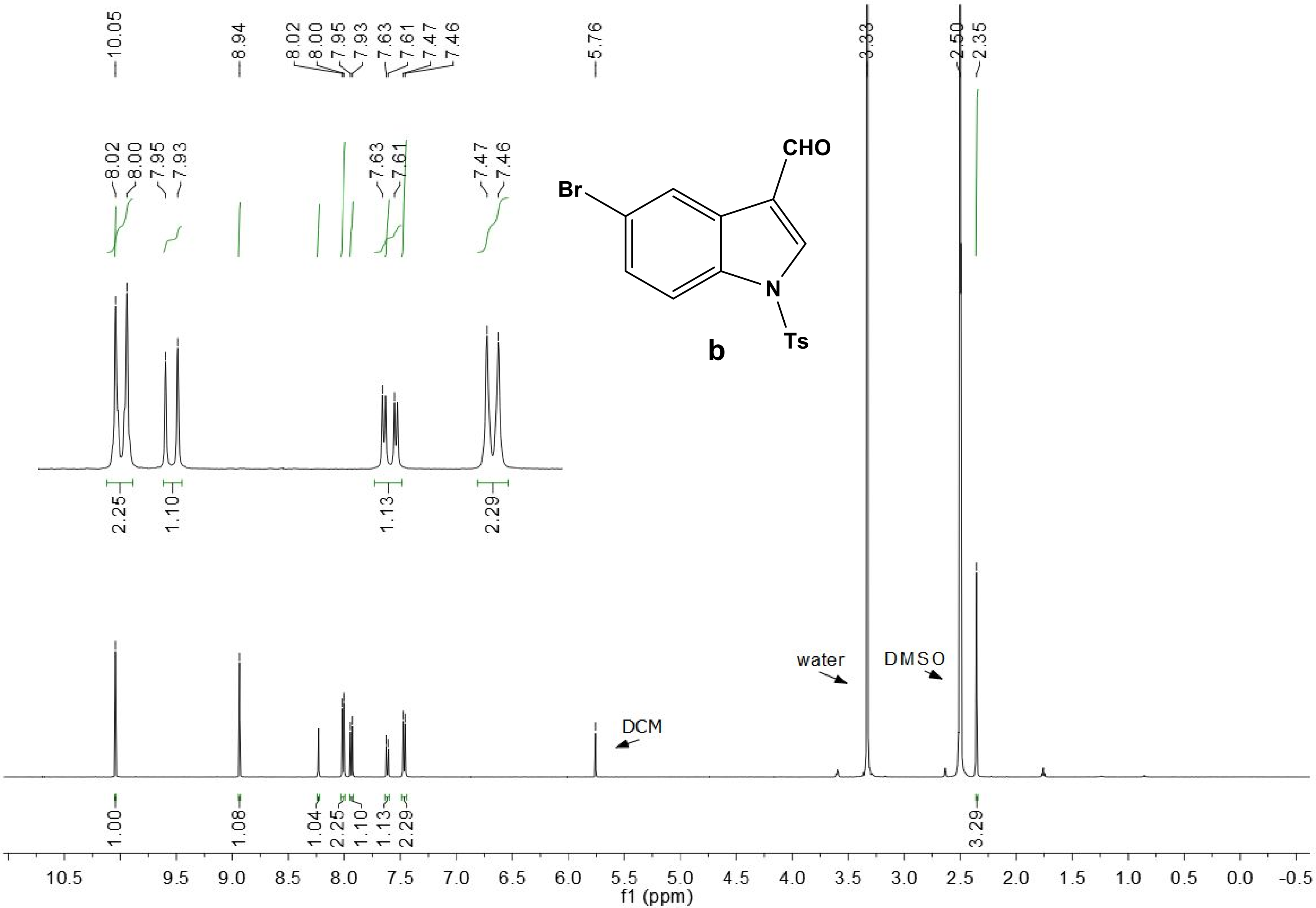

Figure S1. ^1H NMR spectrum of **b** in DMSO-d_6.

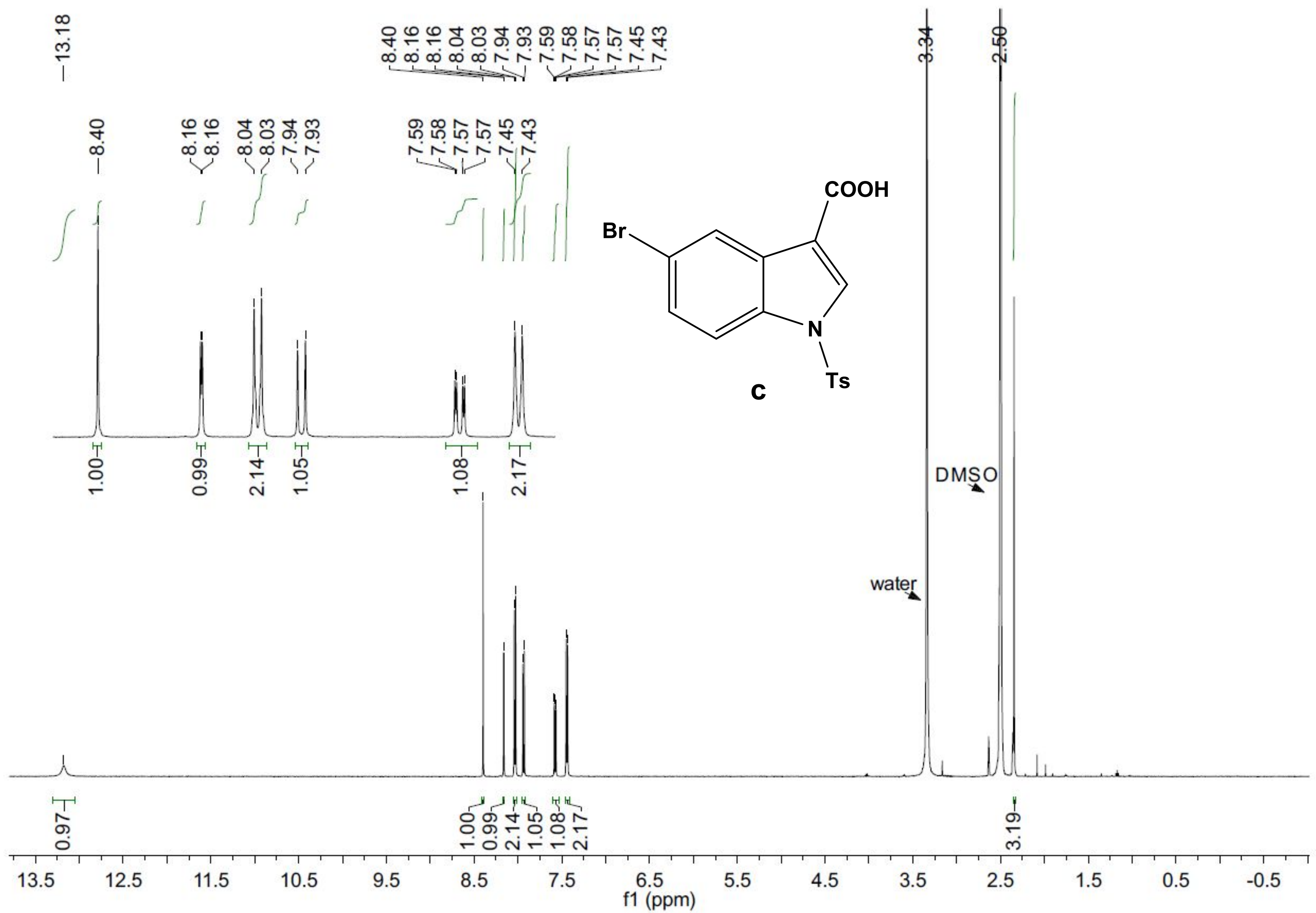

Figure S2. ^{1}H NMR spectrum of **c** in DMSO-d_6.

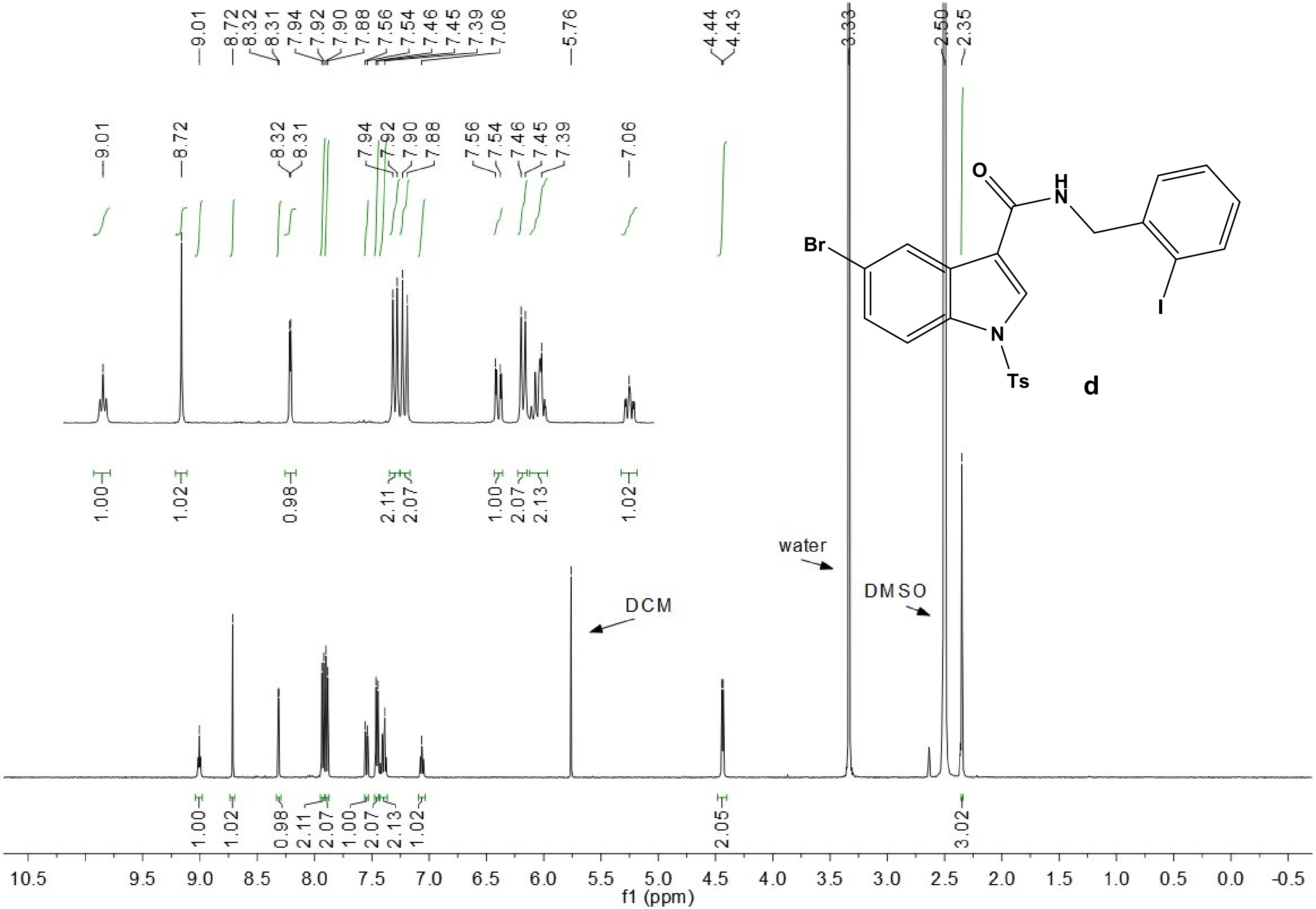

Figure S3. ^{1}H NMR spectrum of **d** in DMSO-d_6.

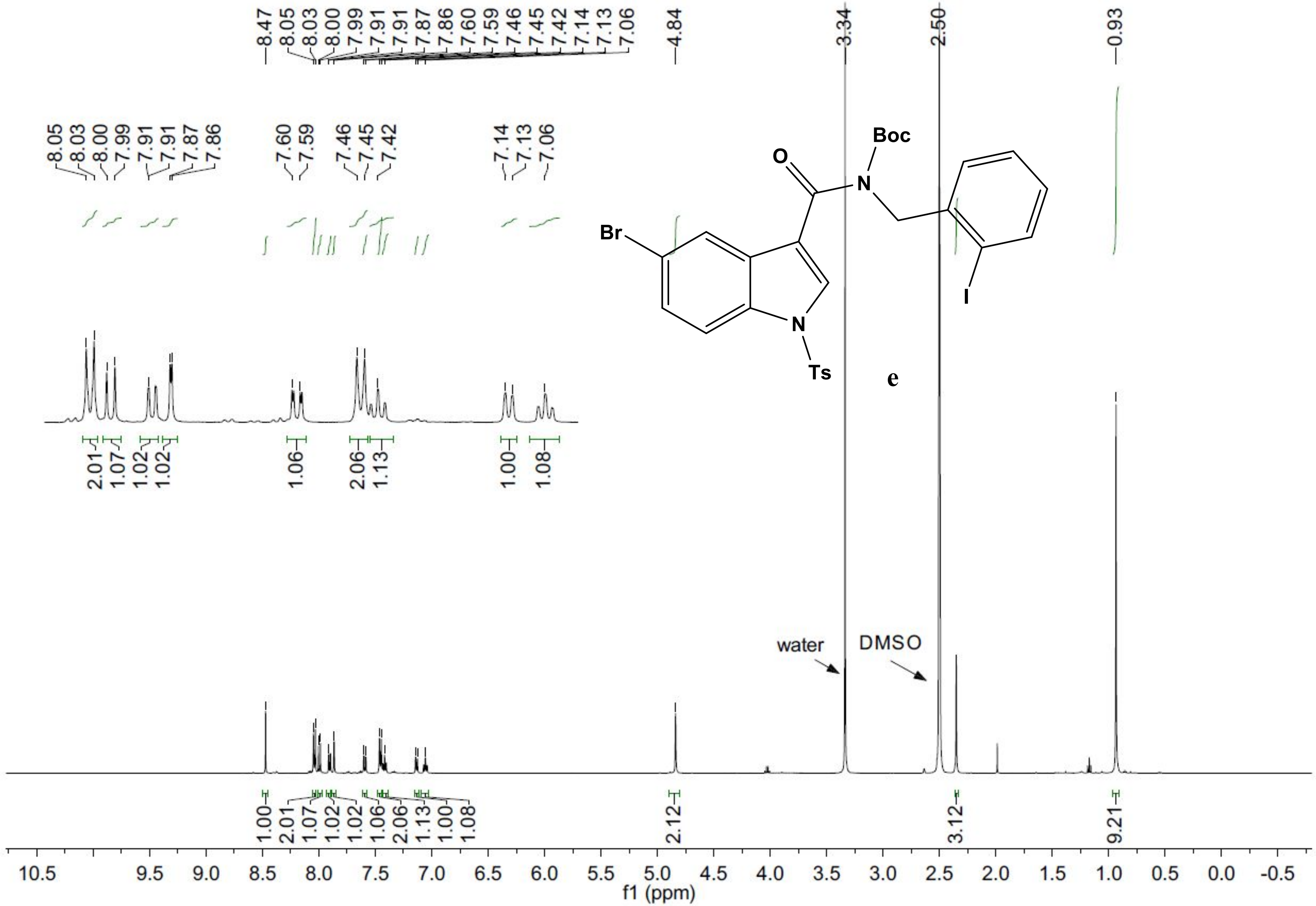

Figure S4. ^{1}H NMR spectrum of **e** in DMSO-d_6.

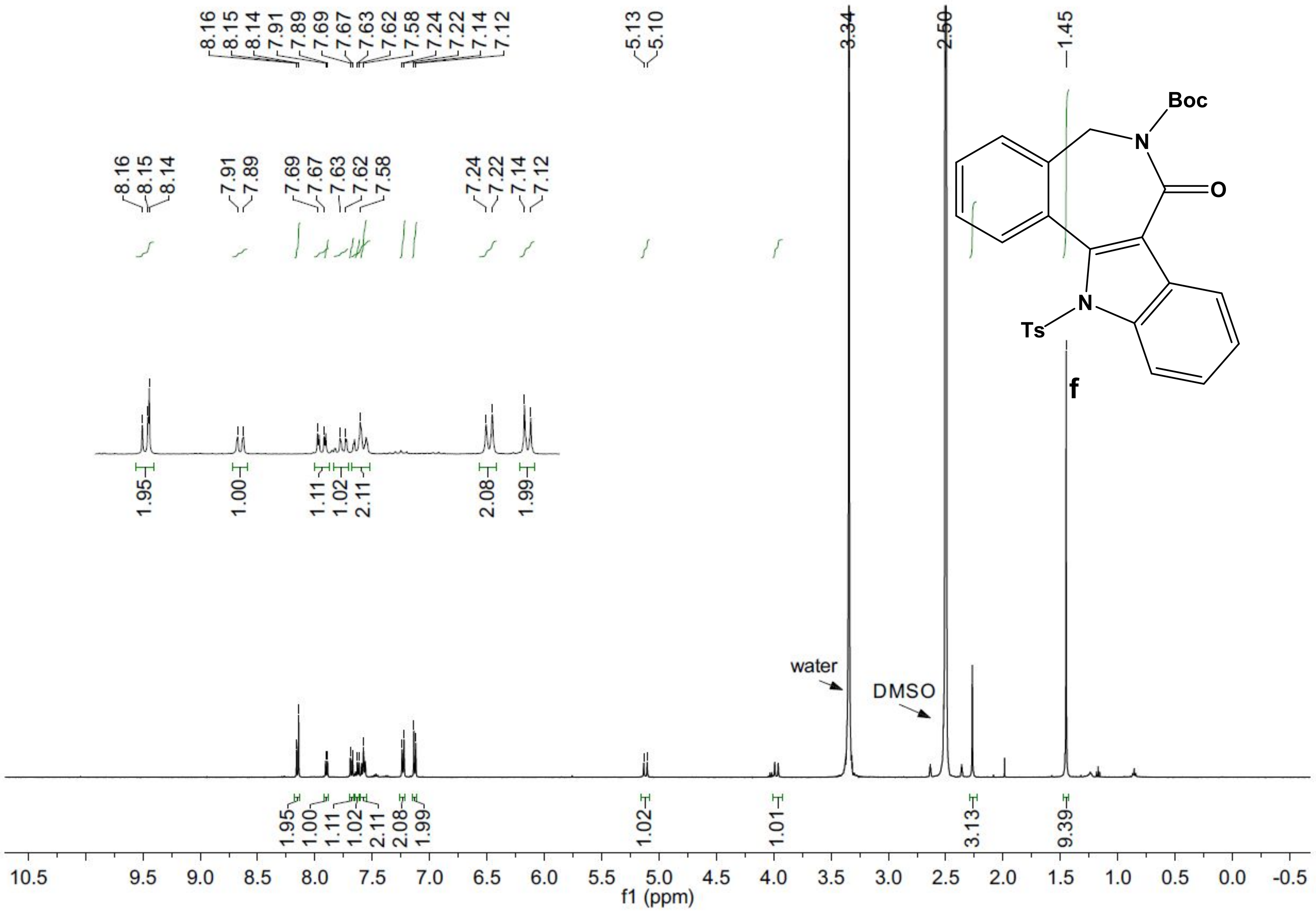

Figure S5. ^{1}H NMR spectrum of **f** in DMSO-d_6.

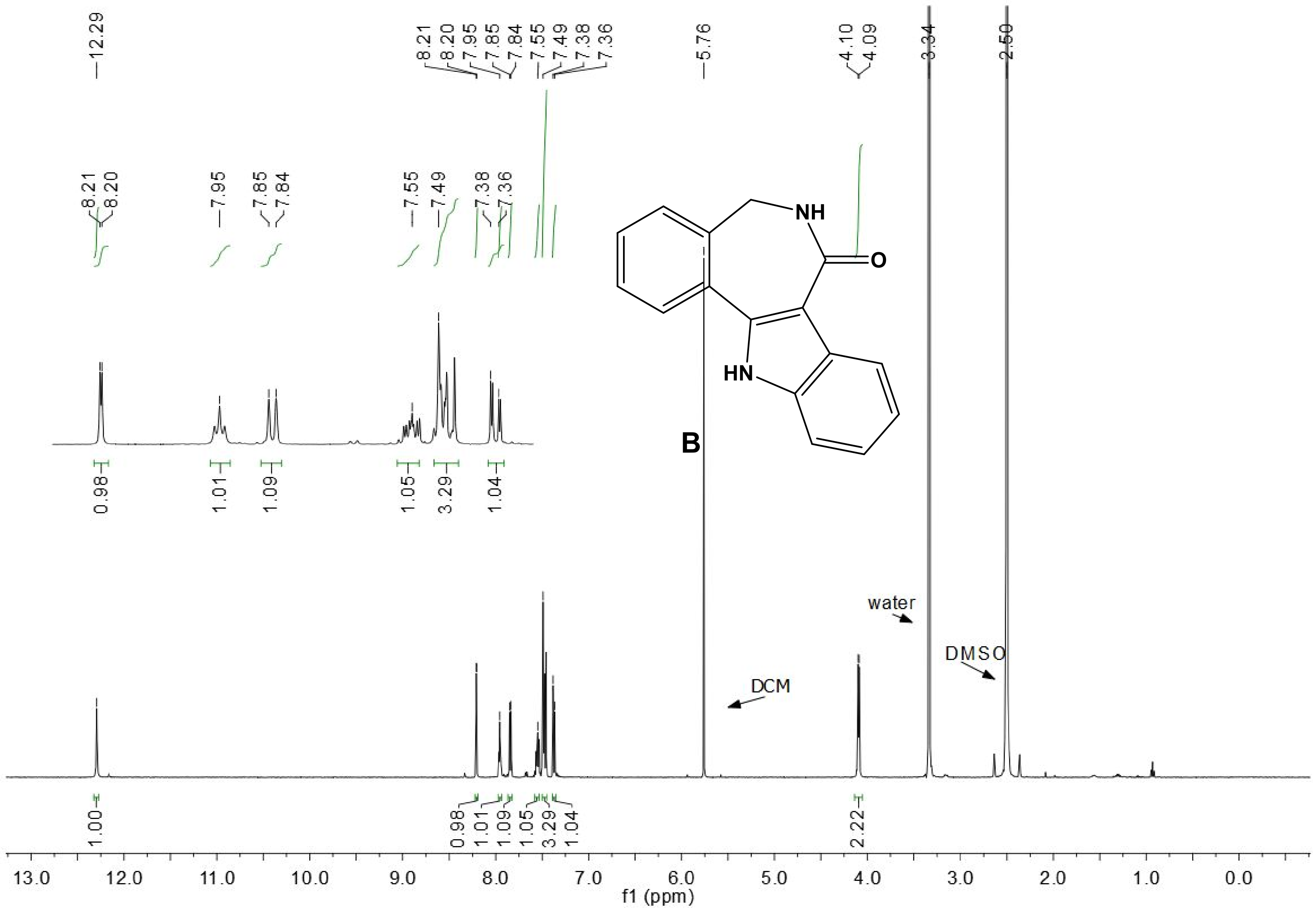

Figure S6. ^{1}H NMR spectrum of **B** in DMSO-d_6.

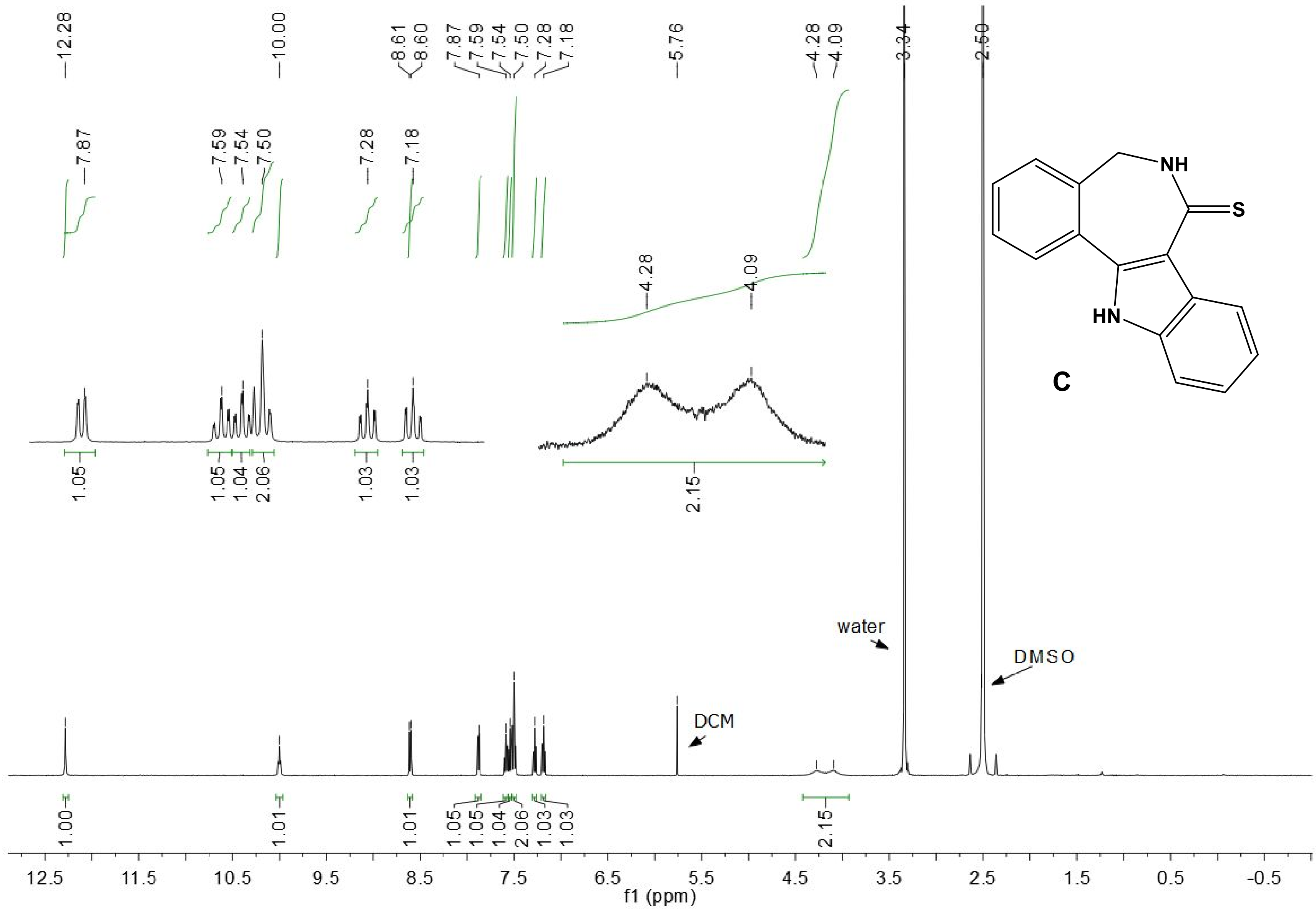

Figure S7. ^{1}H NMR spectrum of **C** in DMSO-d_6.

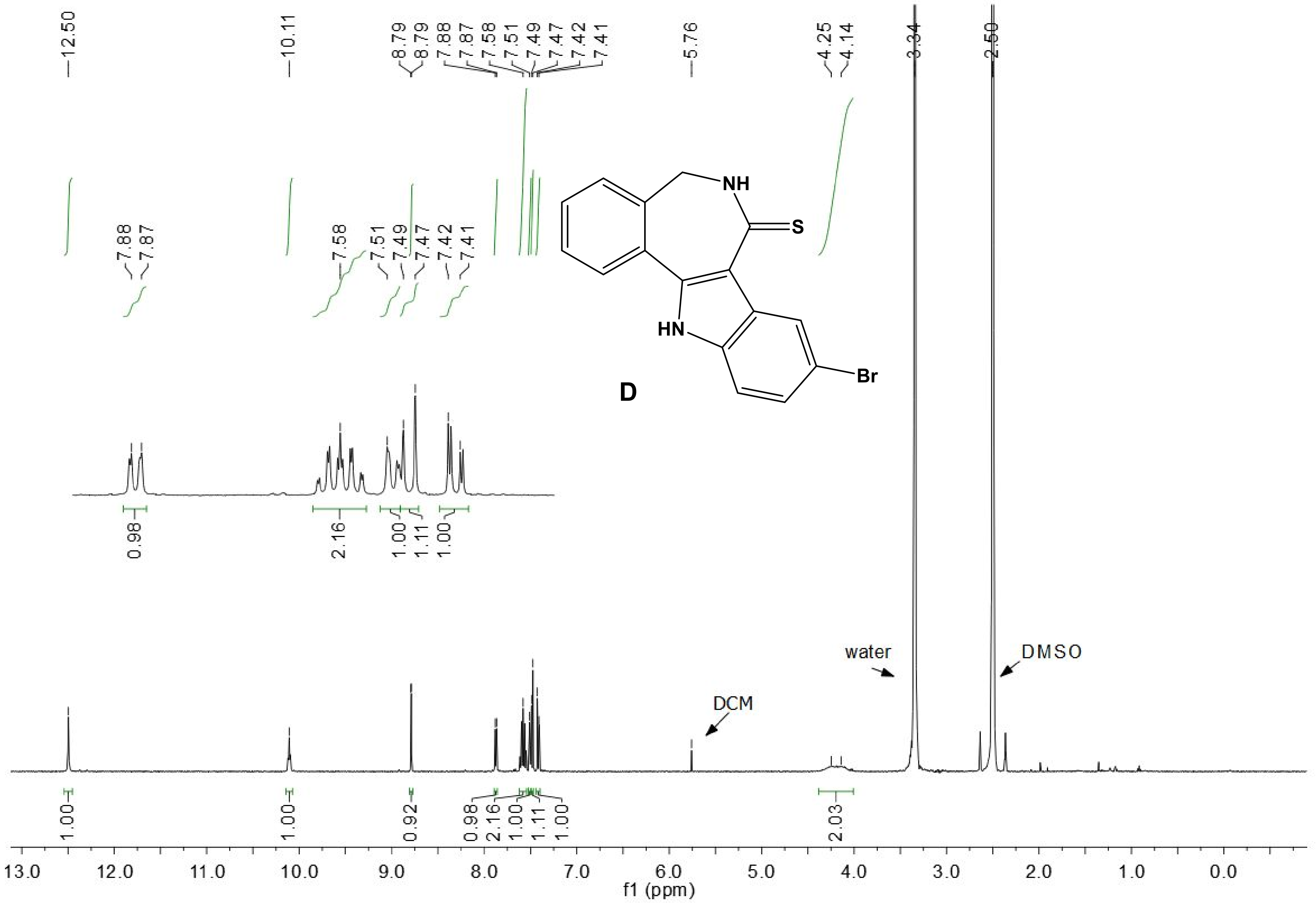

Figure S8. ^{1}H NMR spectrum of **D** in DMSO-d^{6}.

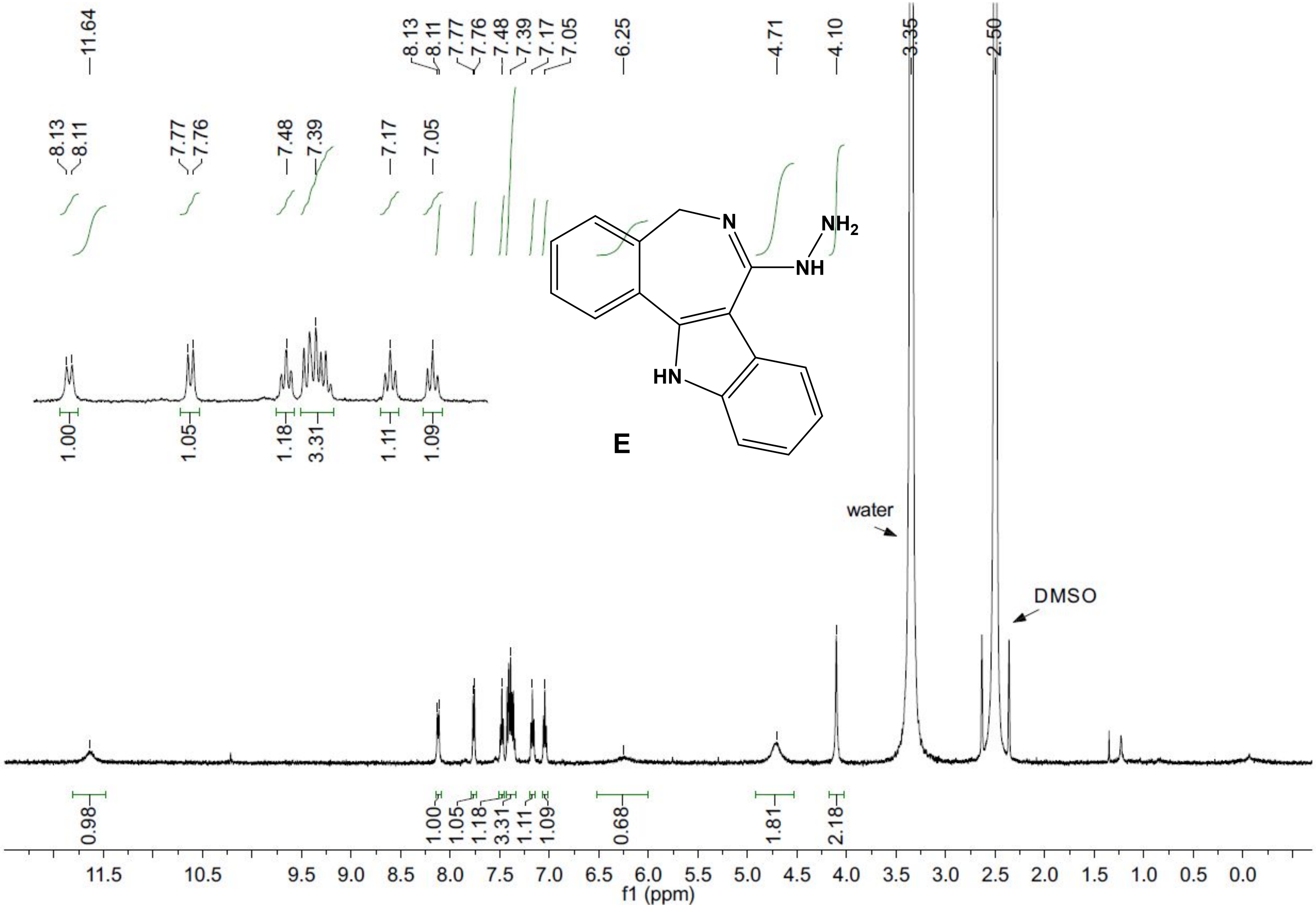

Figure S9. ^{1}H NMR spectrum of **E** in DMSO-d_6.

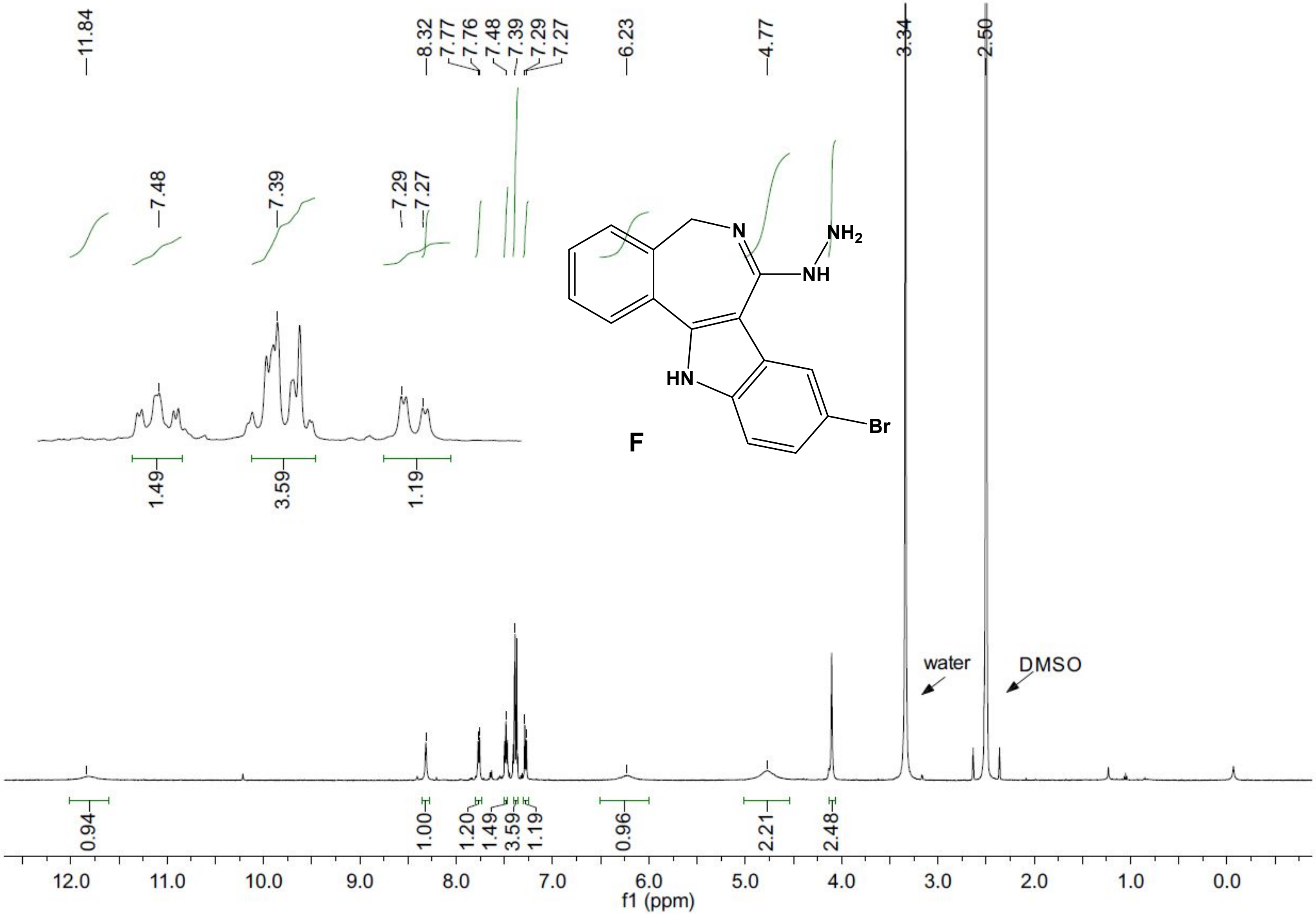

Figure S10. ^{1}H NMR spectrum of **F** in DMSO-d_6.

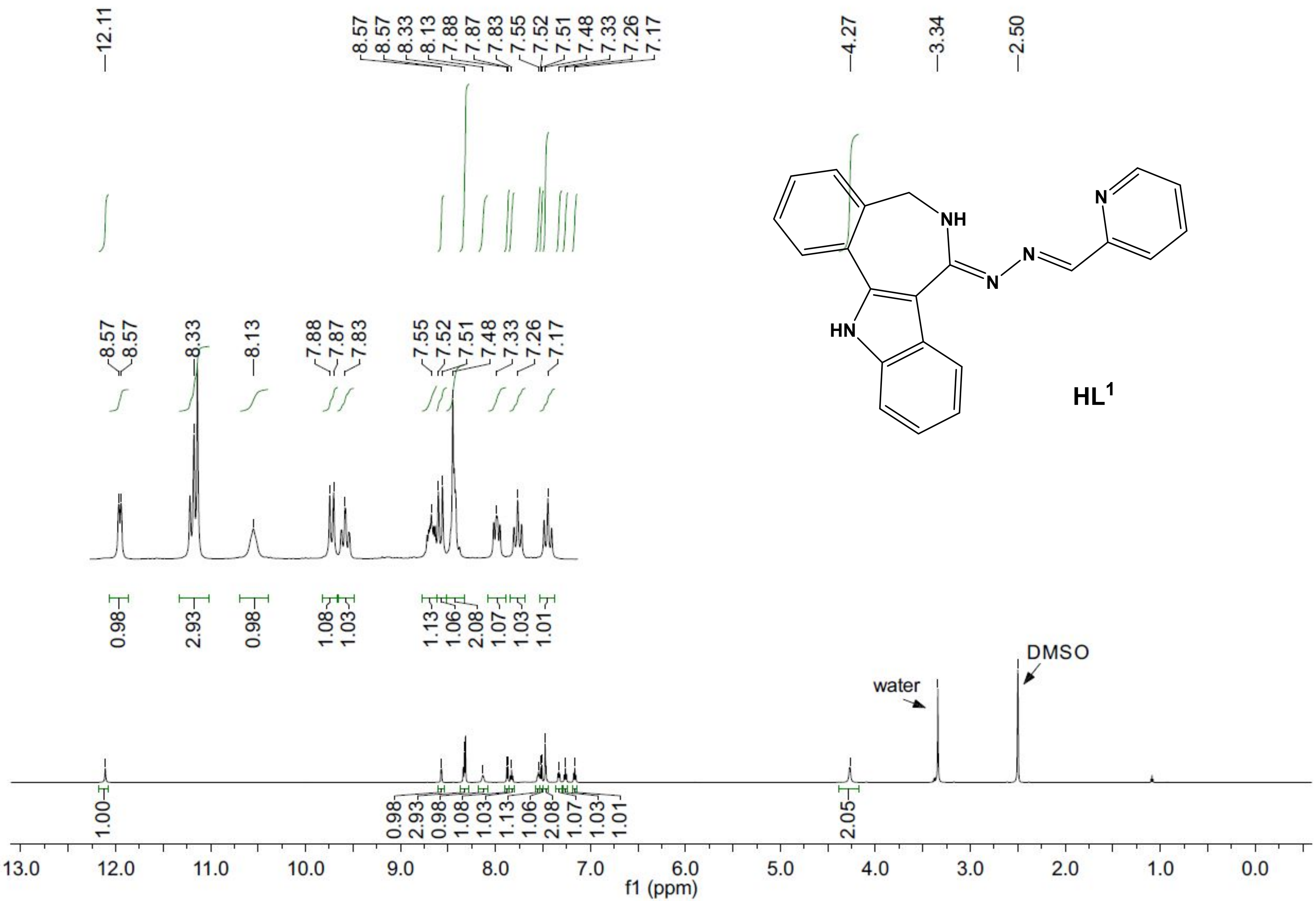

Figure S11. ^{1}H NMR spectrum of **HL^{1}** in DMSO-d_6.

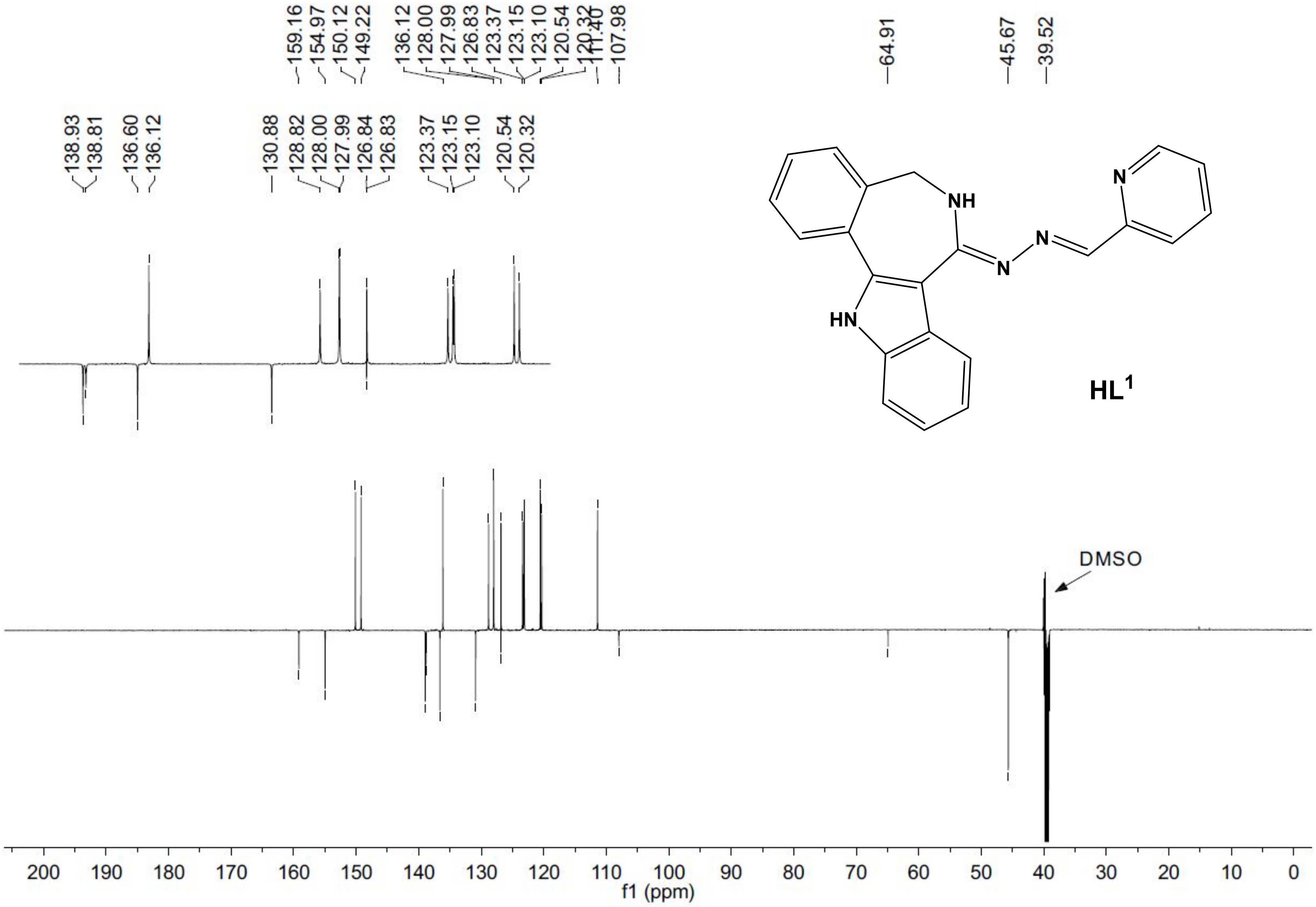

Figure S12. ^{13}C NMR spectrum of **HL^{1}** in DMSO-d_6.

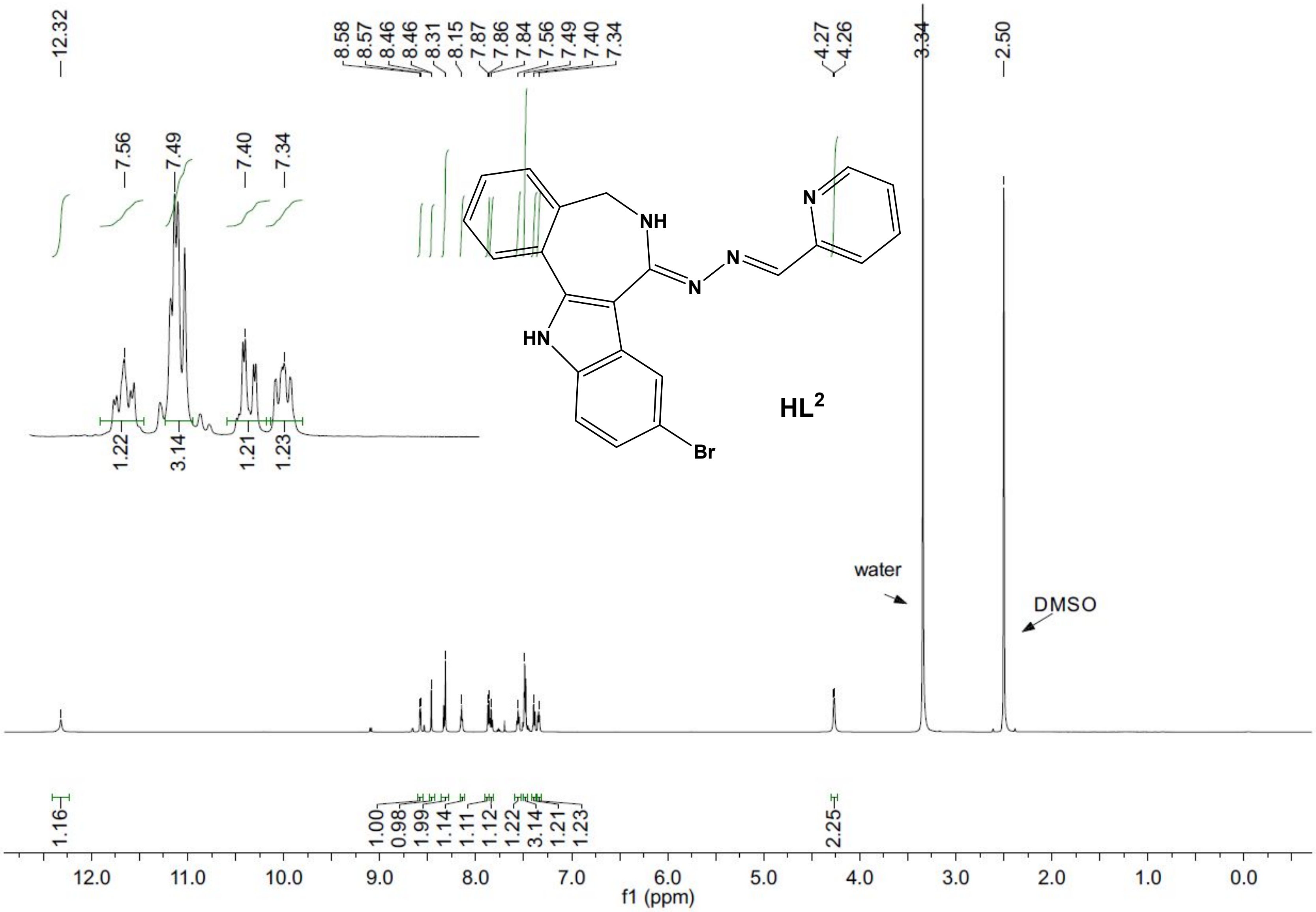

Figure S13. ^{1}H NMR spectrum of **HL^2** in DMSO-d_6.

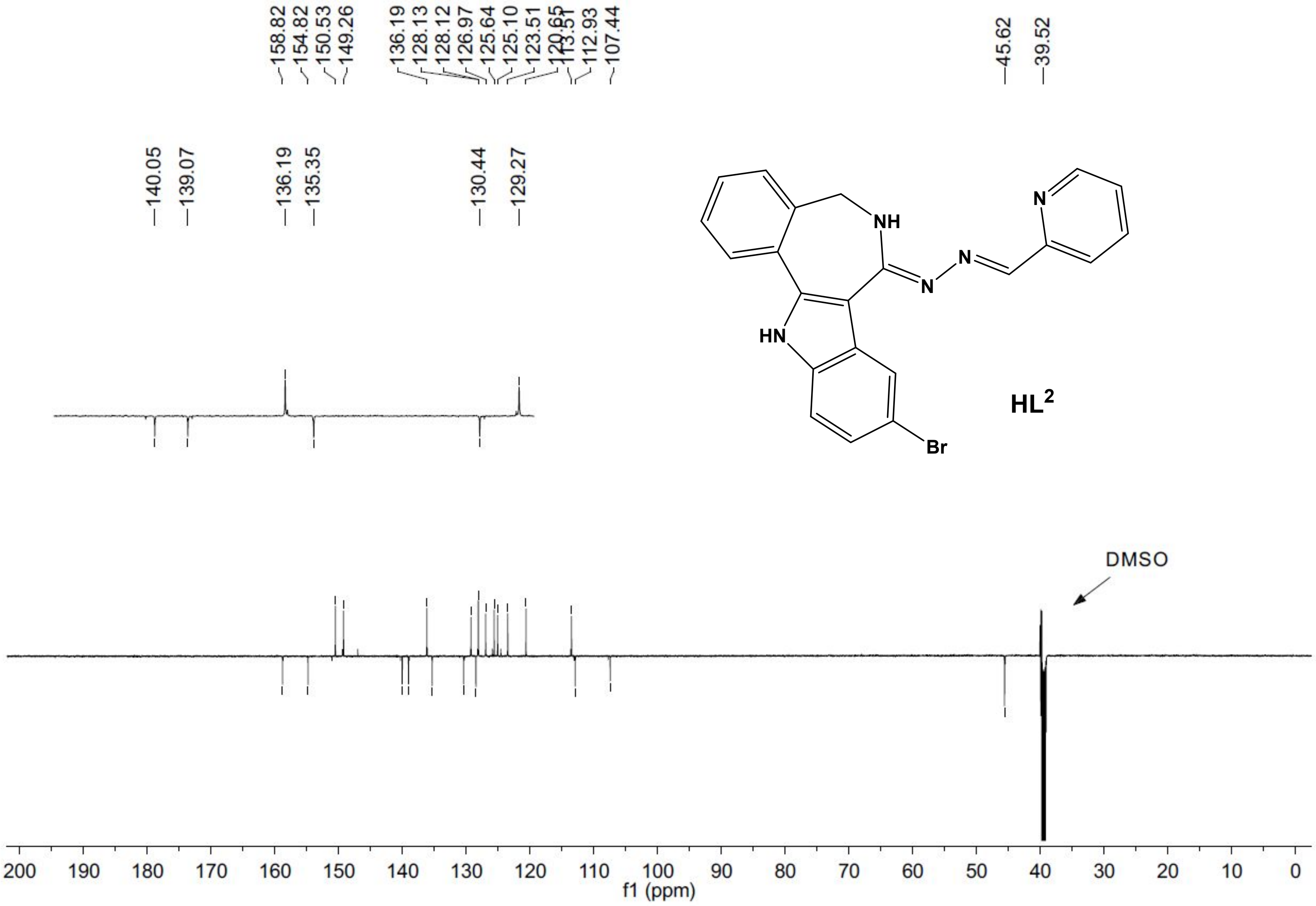

Figure S14. ^{13}C NMR spectrum of **HL^2** in DMSO-d_6.

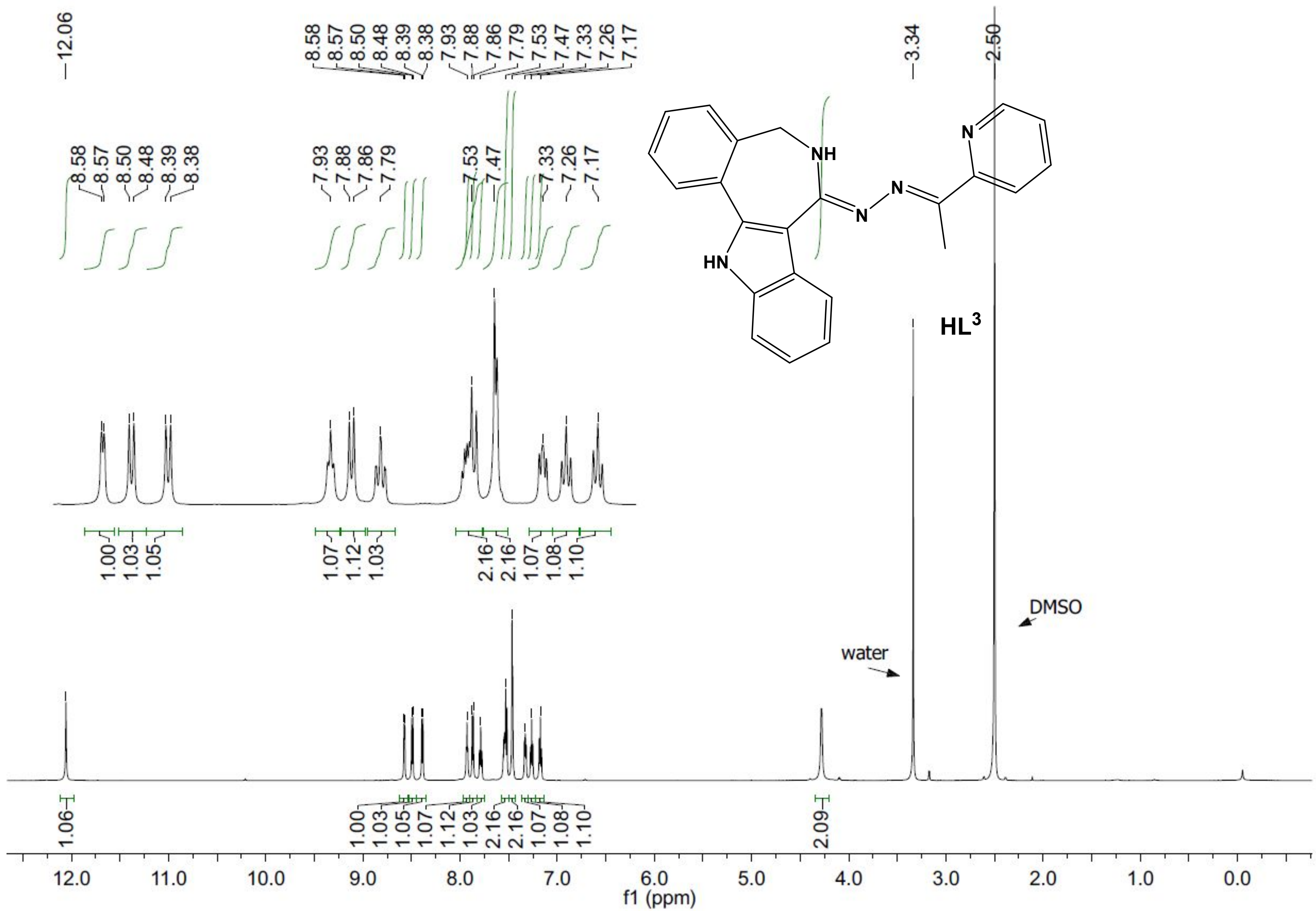

Figure S15. ^{1}H NMR spectrum of **HL^{3}** in DMSO-d_6.

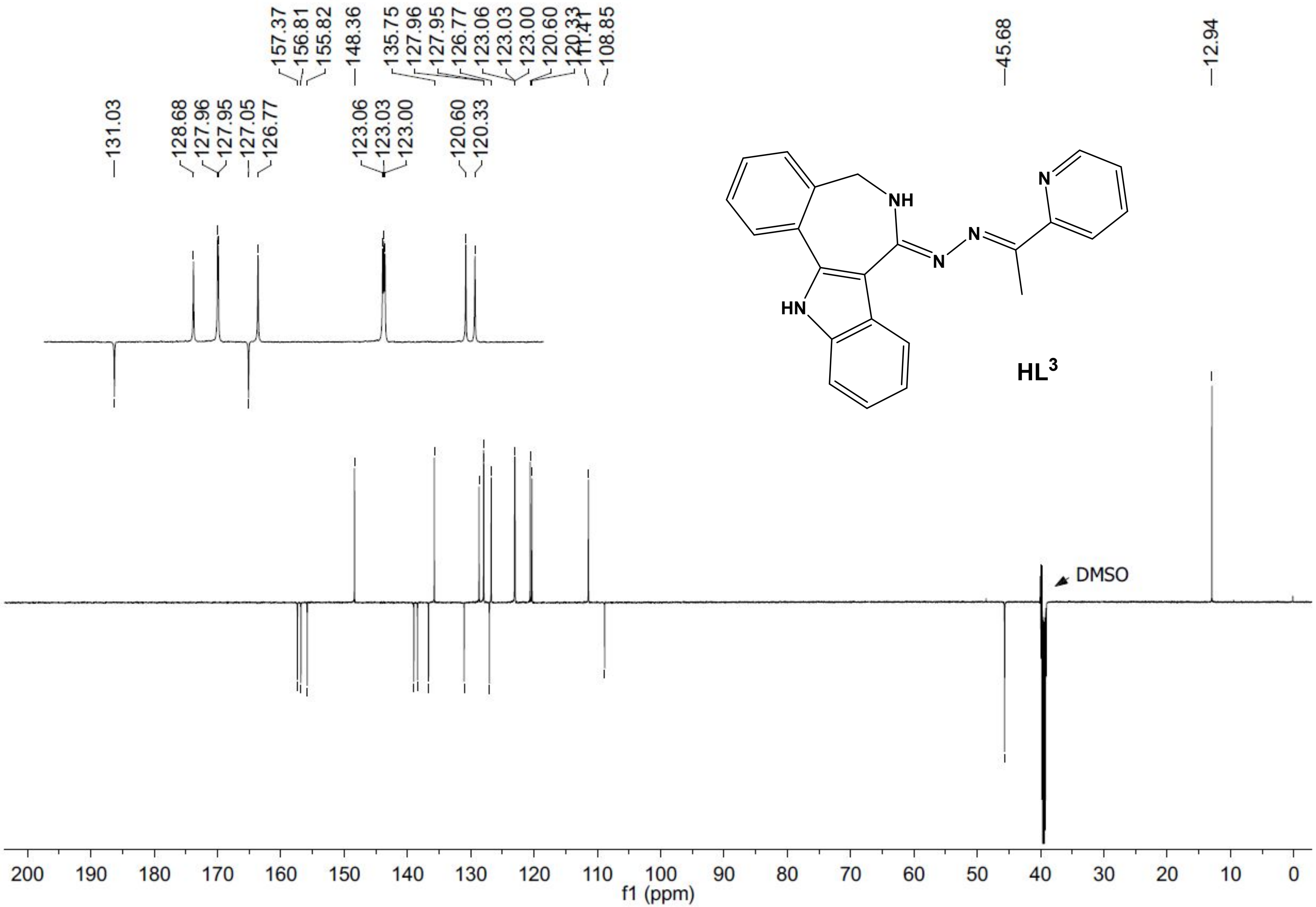

Figure S16. ^{13}C NMR spectrum of **HL^3** in DMSO-d_6.

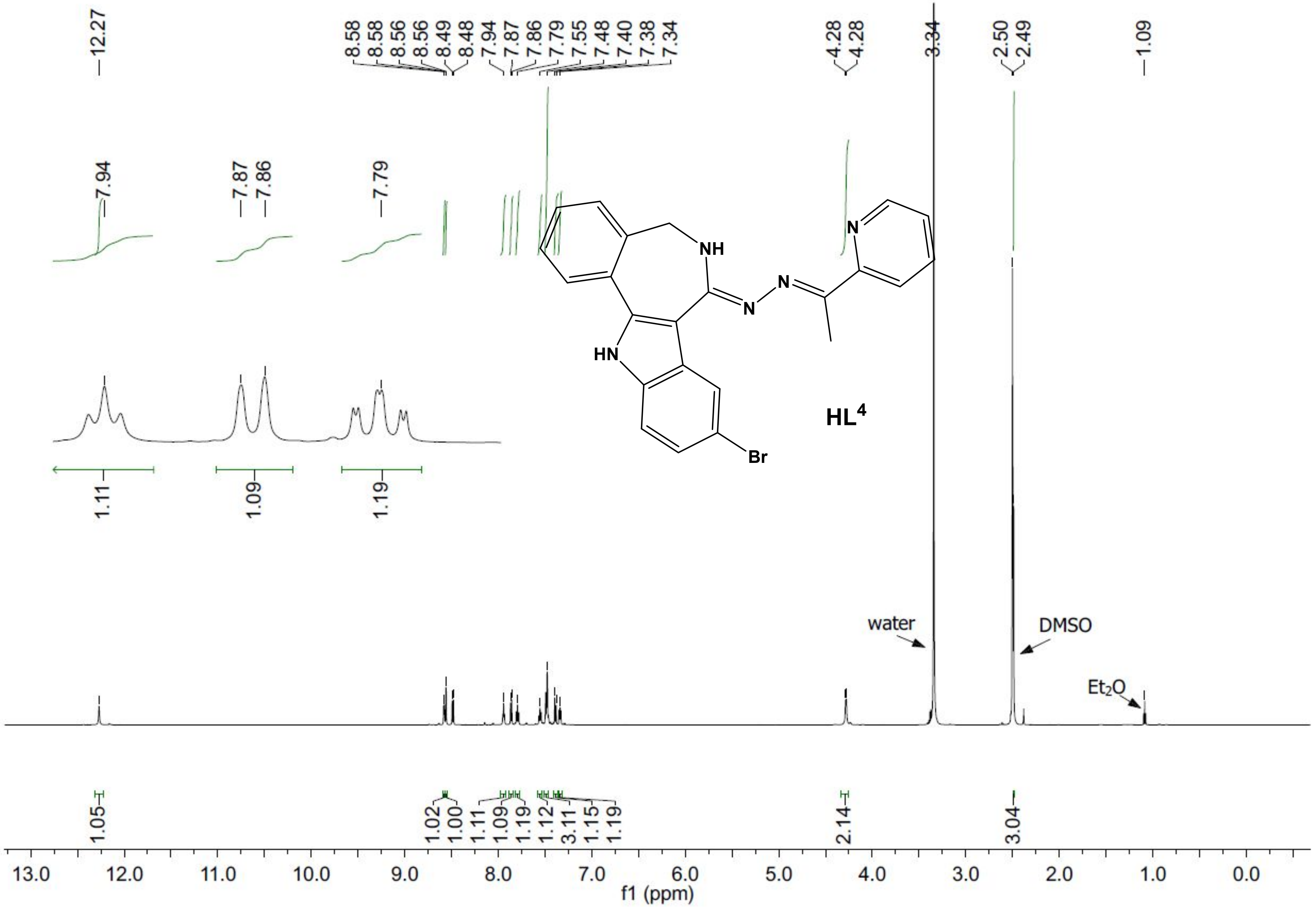

Figure S17. ^{1}H NMR spectrum of **HL⁴** in DMSO-d_6.

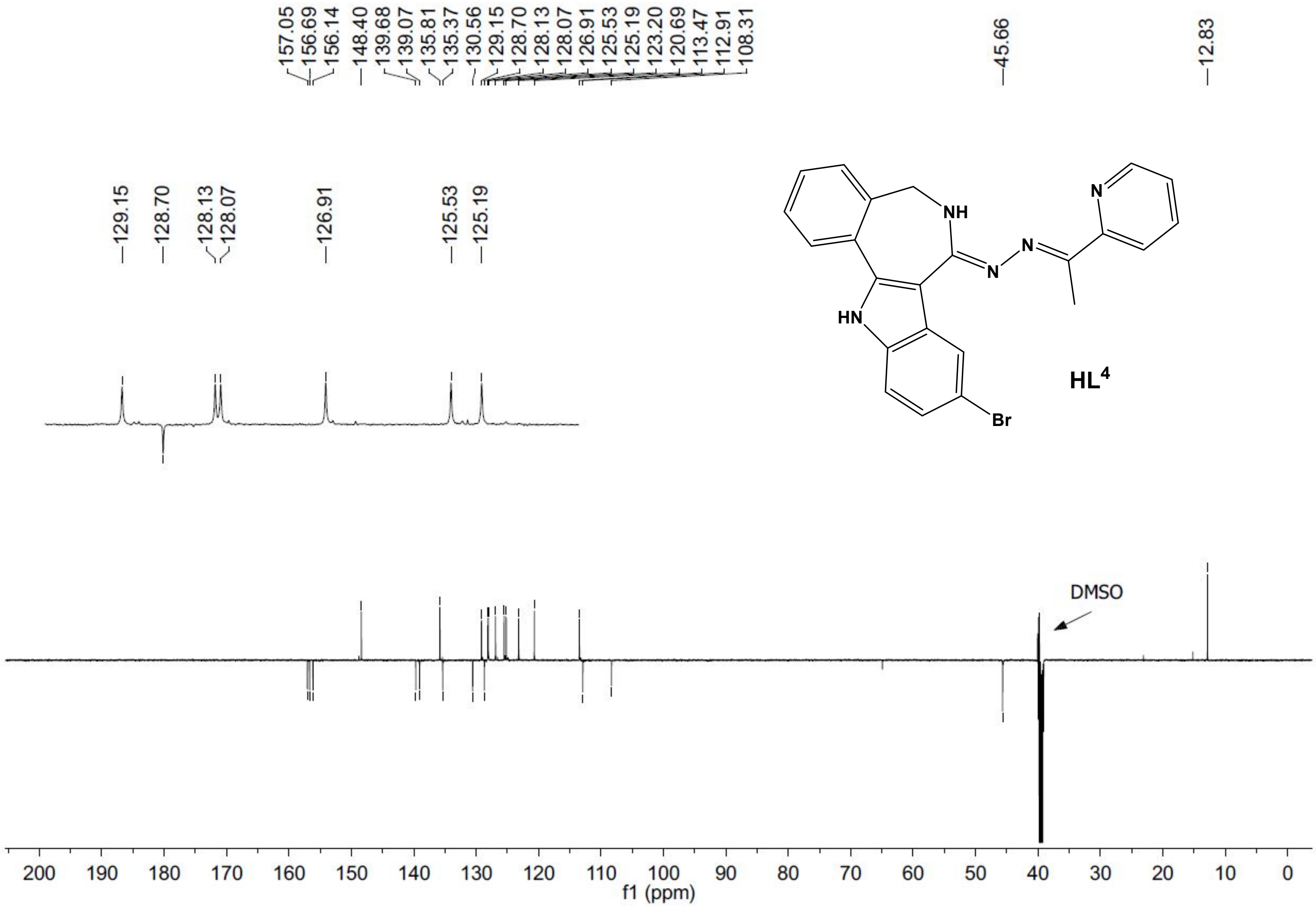

Figure S18. ^{13}C NMR spectrum of **HL⁴** in DMSO-d_6.

Generic Display Report

Analysis Info

Analysis Name	D:\Data\MS_MessService\66934_IRKU140_amazon.d	Acquisition Date	10/18/2019 10:10:50 AM
Method	MSC-Service_direct-injection.m	Operator	MSC
Sample Name	66934_IRKU140_amazon	Instrument	amaZon speed ETD
Comment	Kuznetcova / Anorg.Chem. ACN / MeOH + 1% H2O		

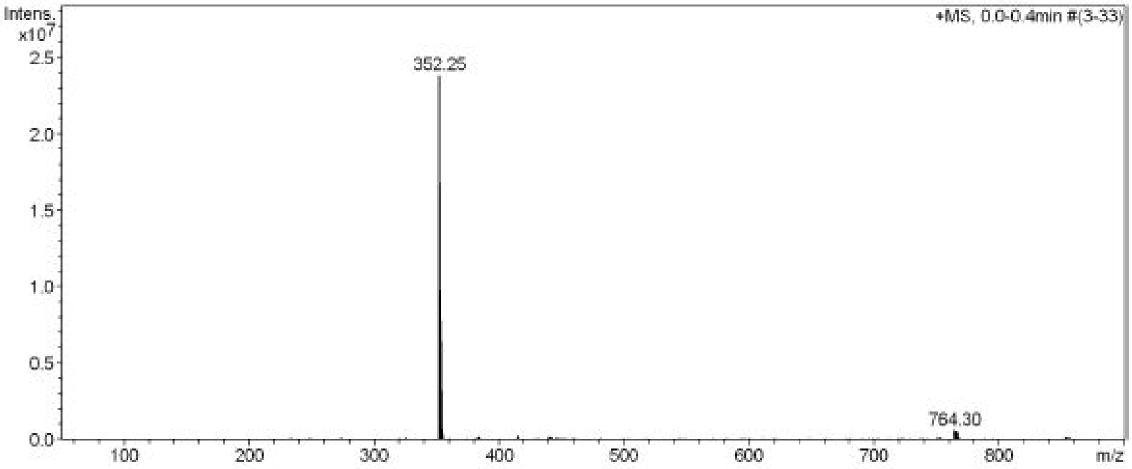

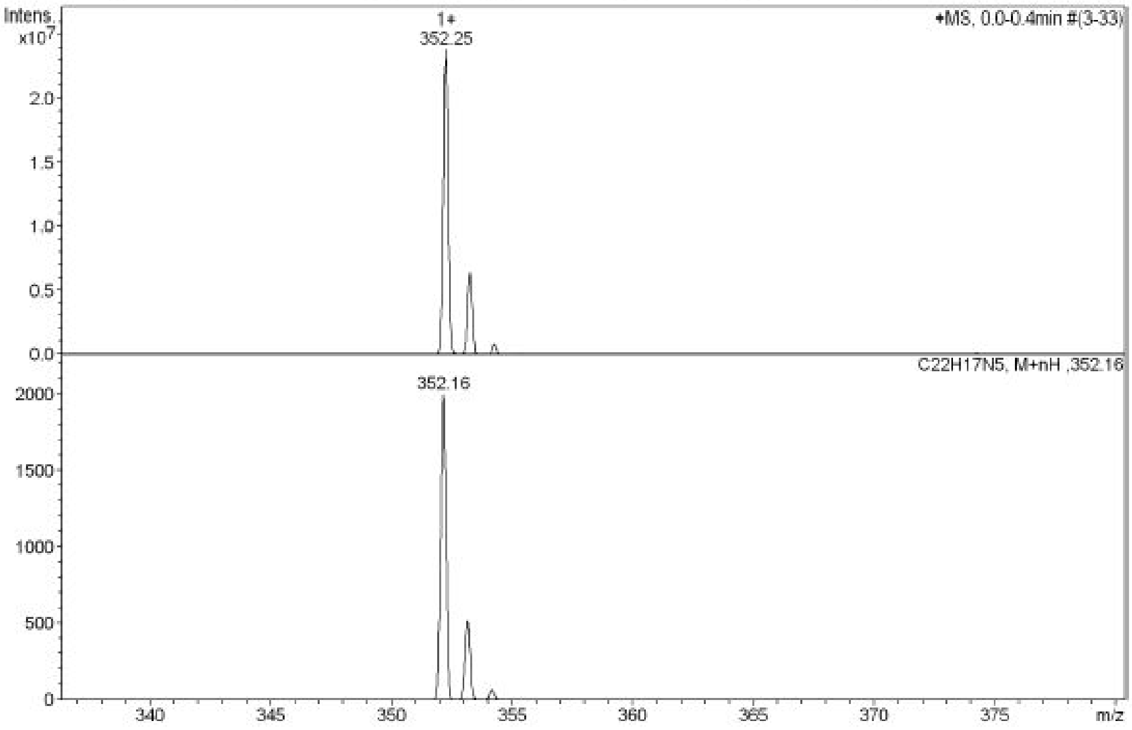

Bruker Compass DataAnalysis 4.0 printed: 10/18/2019 10:13:14 AM Page 1 of 1

Figure S19. ESI mass spectrum of **HL[1]**.

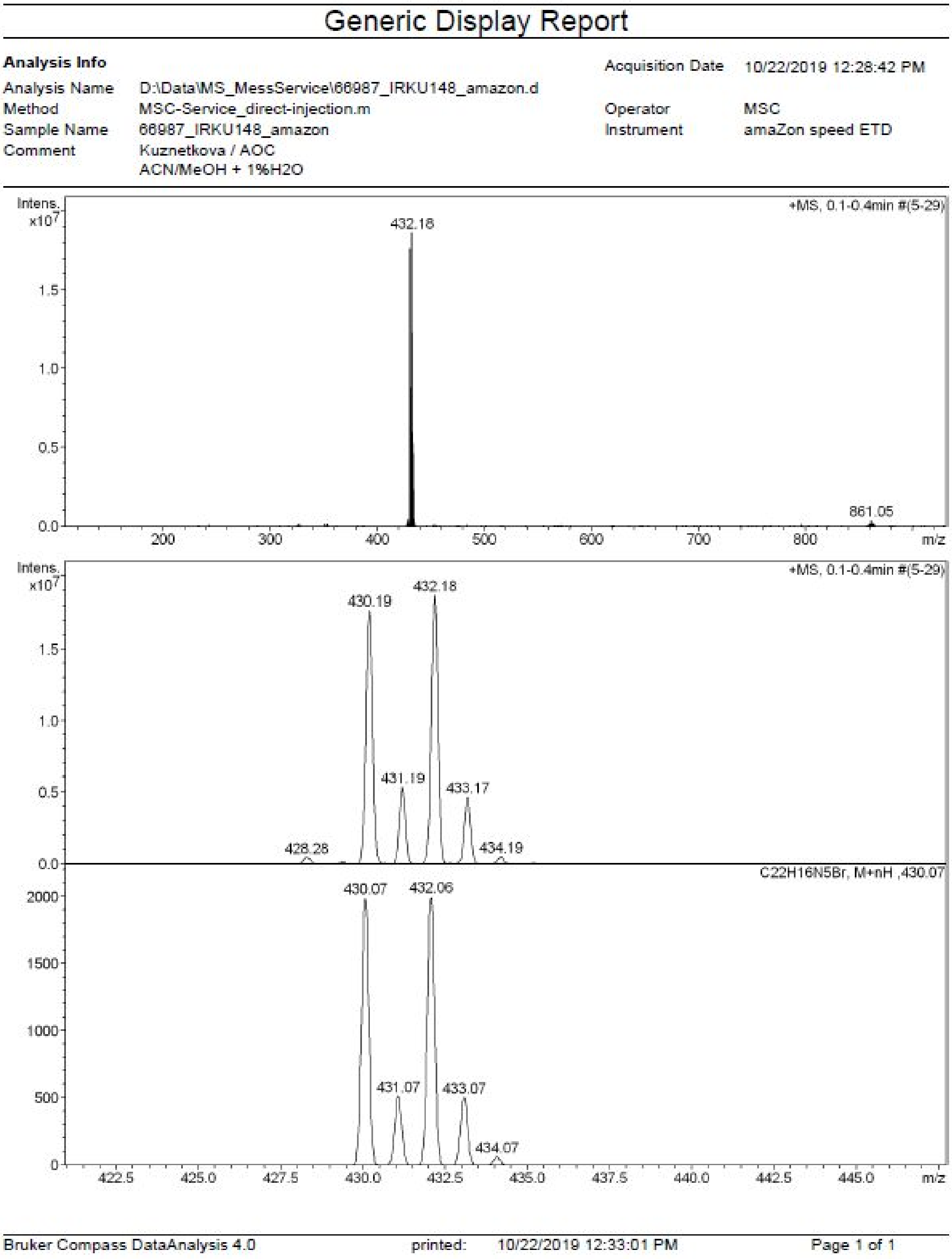

Figure S20. ESI mass spectrum of **HL**$^{\mathbf{2}}$**.**

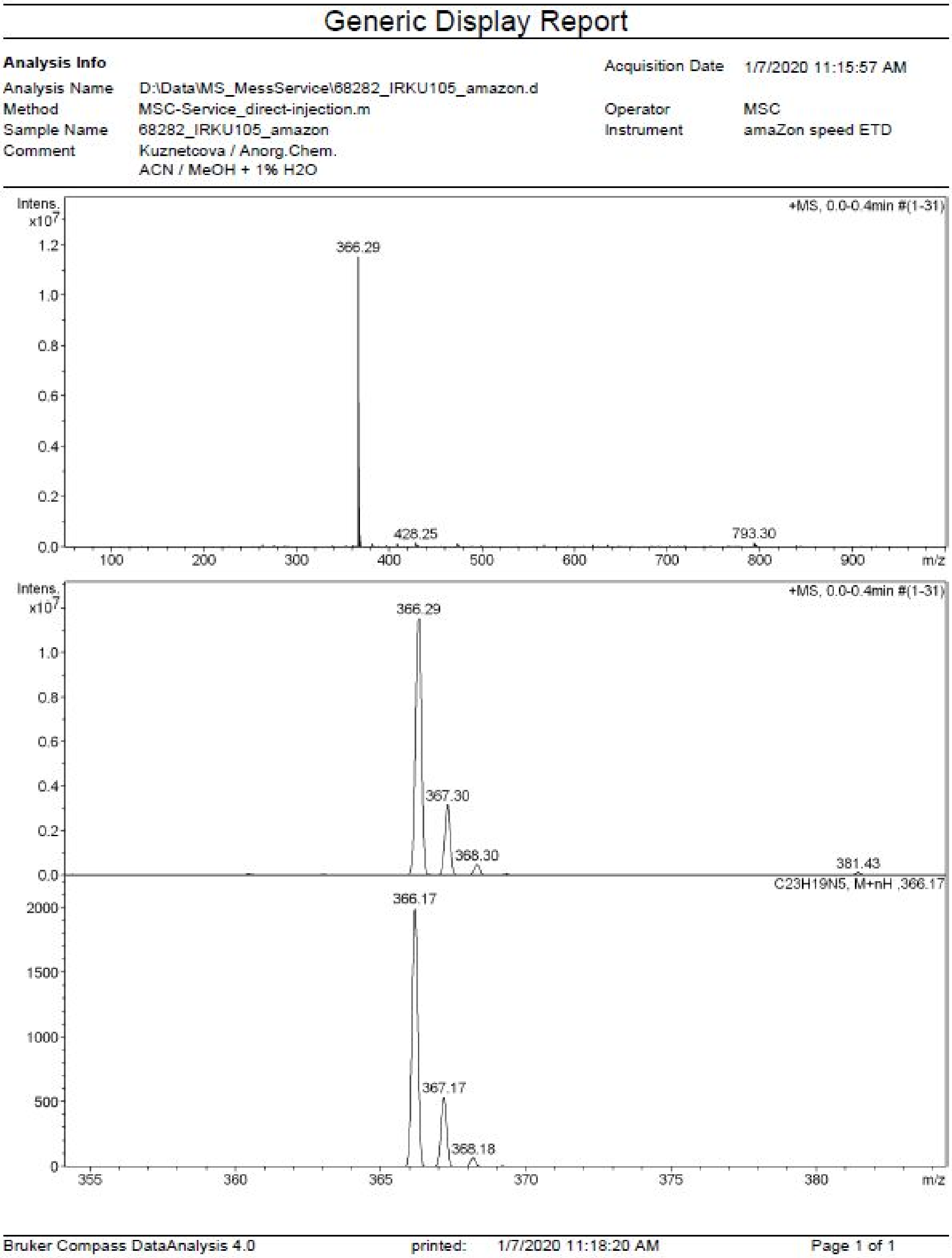

Figure S21. ESI mass spectrum of **HL^3**.

Generic Display Report

Analysis Info

Analysis Name	D:\Data\MS_MessService\66986_IRKU147_amazon.d	Acquisition Date	10/22/2019 12:21:30 PM
Method	MSC-Service_direct-injection.m	Operator	MSC
Sample Name	66986_IRKU147_amazon	Instrument	amaZon speed ETD
Comment	Kuznetkova / AOC<br>ACN/MeOH + 1%H2O		

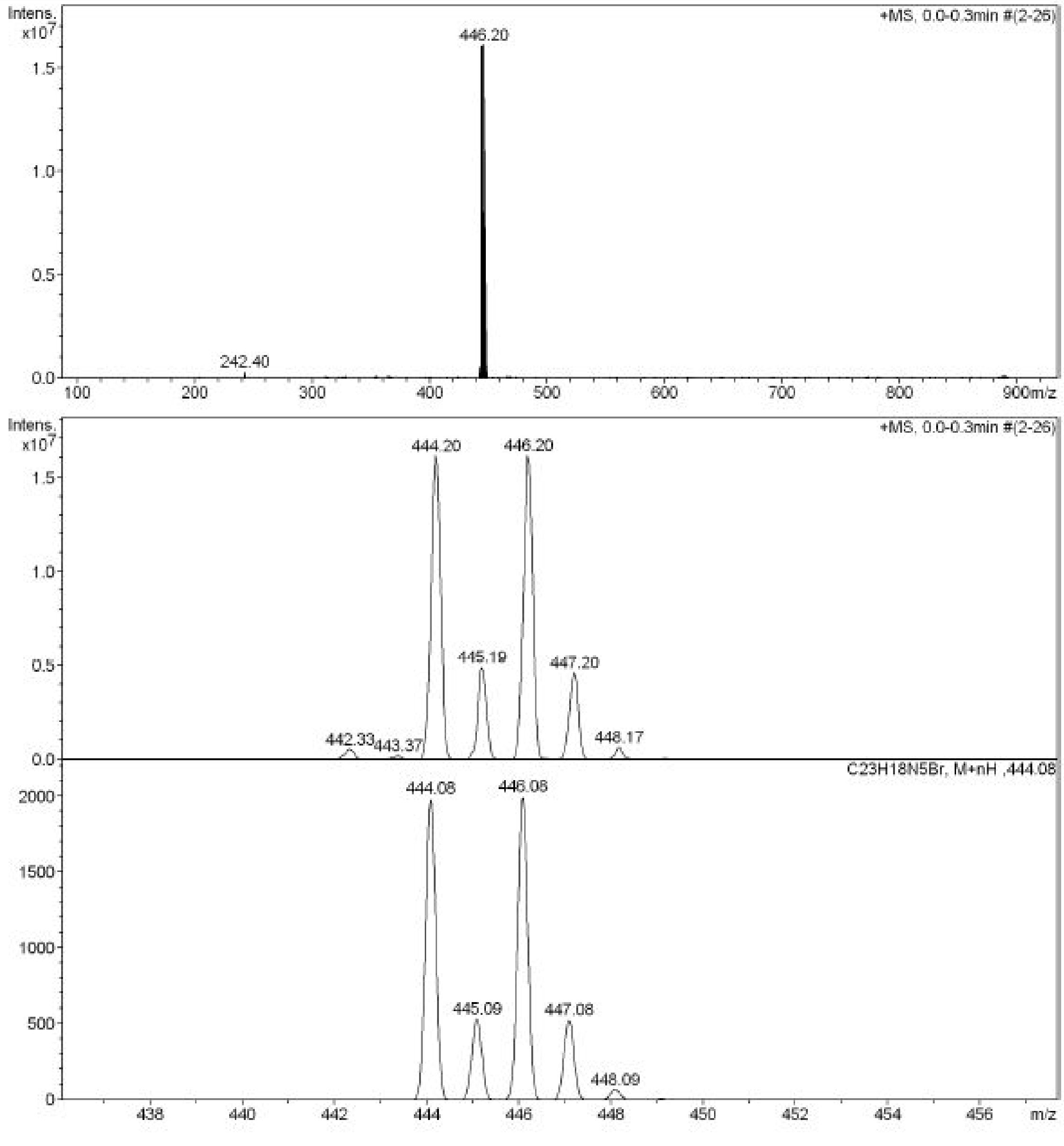

Bruker Compass DataAnalysis 4.0 printed: 10/22/2019 12:25:09 PM Page 1 of 1

Figure S22. ESI mass spectrum of **HL⁴**.

High Performance Liquid Chromatography-MS report of 1

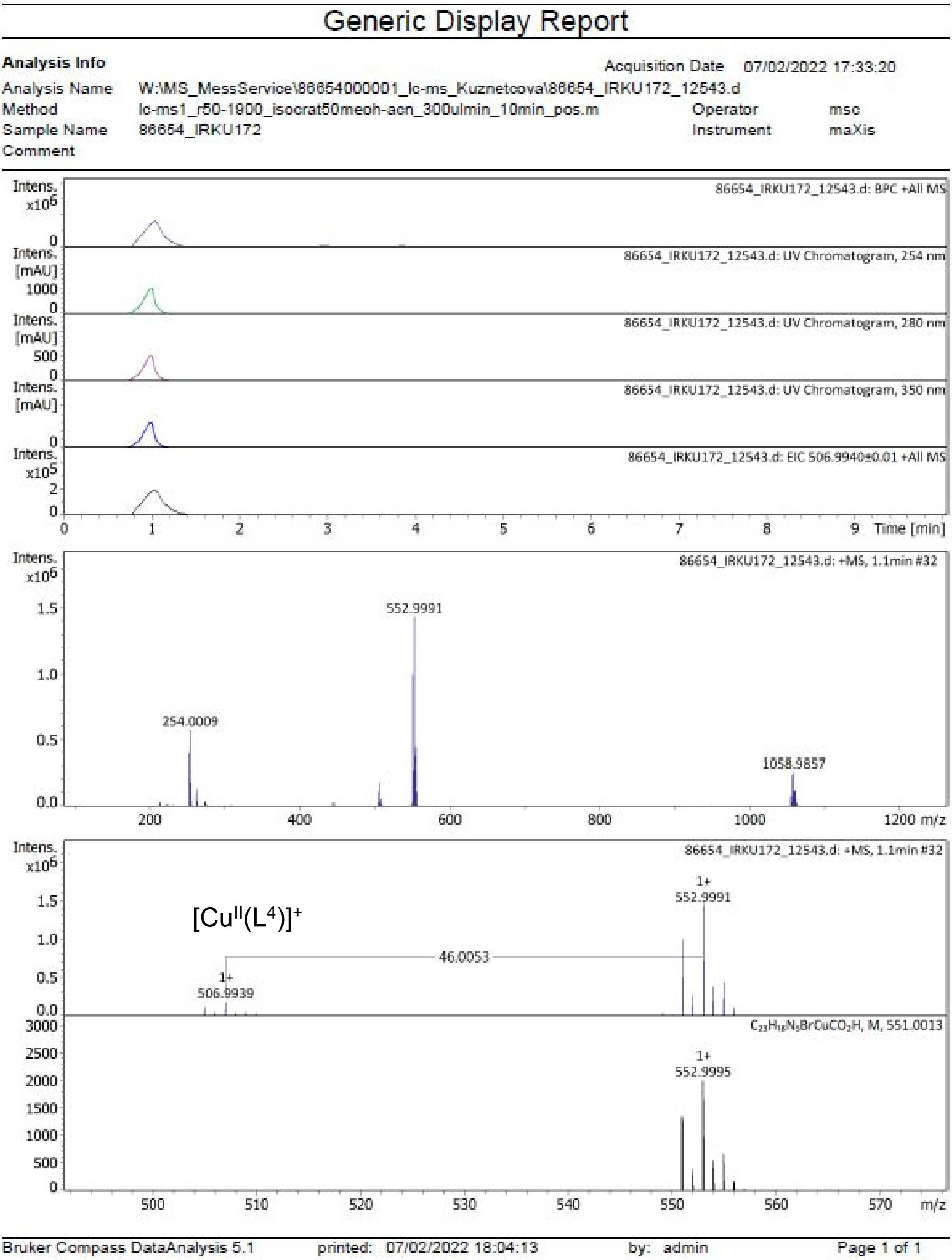

Figure S23. Purity control of complex **4** via HPLC and HR ESI MS. The peak with *m*/*z* 552.9995 is attributed to $[Cu^{II}(HL^4)(HCOO)]^+$.

Generic Display Report

**Analysis Info**		Acquisition Date	10/31/2019 7:02:18 AM
Analysis Name	D:\Data\MS_MessService\67224_IRKU144_amazon.d		
Method	MSC-Service_direct-injection.m	Operator	MSC
Sample Name	67224_IRKU144_amazon	Instrument	amaZon speed ETD
Comment	Kuznetcova/Anorg.Chem. ACN / MeOH + 1% H2O		

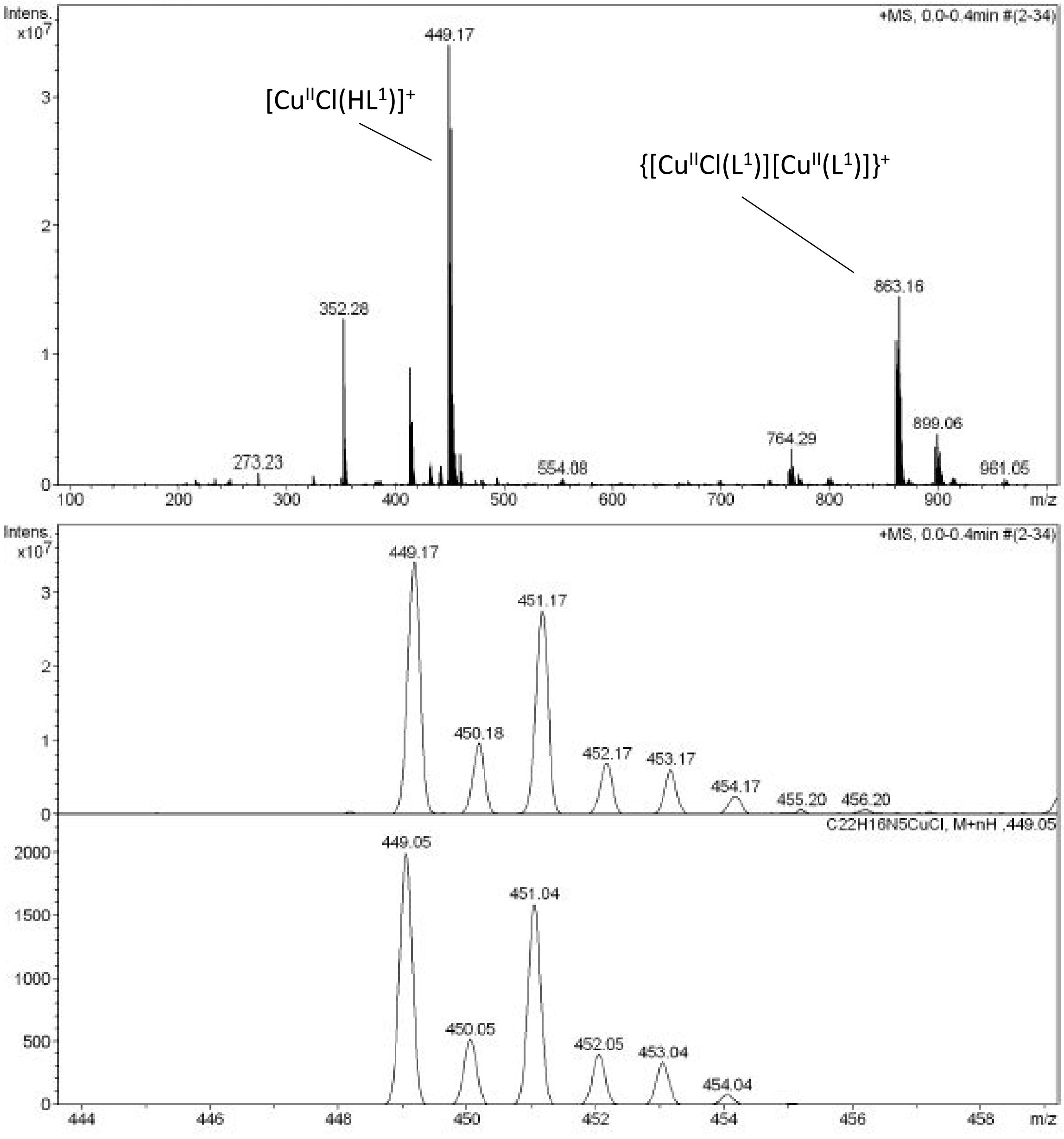

Bruker Compass DataAnalysis 4.0 printed: 10/31/2019 7:09:07 AM Page 1 of 1

Figure S24. High resolution ESI mass spectrum of **1.**

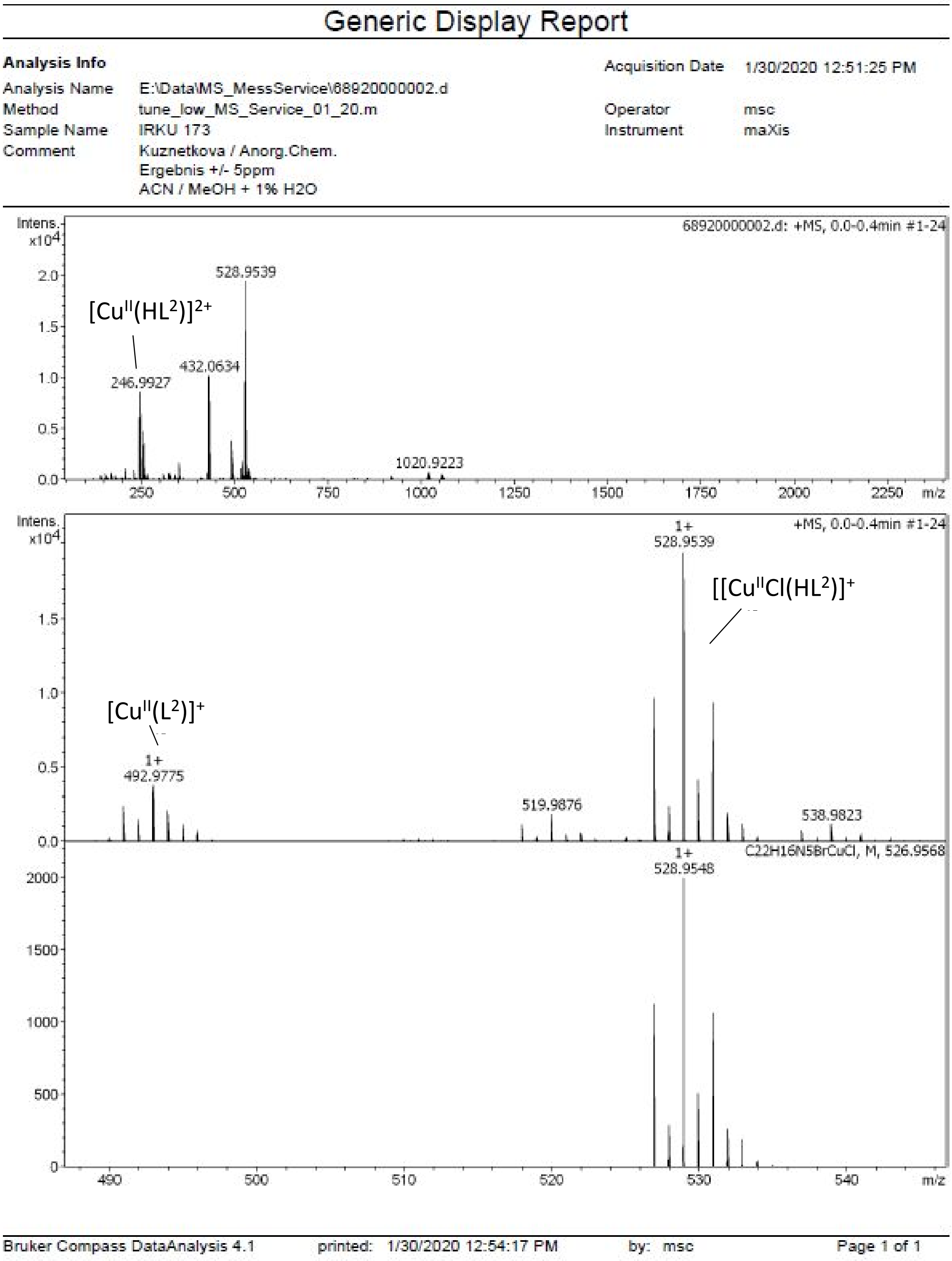

Figure S25. High resolution ESI mass spectrum of **2.**

Generic Display Report

Analysis Info

Analysis Name	E:\Data\MS_MessService\88203000001.d	Acquisition Date	4/14/2022 12:22:34 PM
Method	tune_low_MS_Service_04_22.m	Operator	msc
Sample Name	IRKU121	Instrument	maXis
Comment	Kuznetcova / Anorg.Chem.<br>Ergebnis +/- 5ppm<br>ACN / MeOH + 1% H2O		

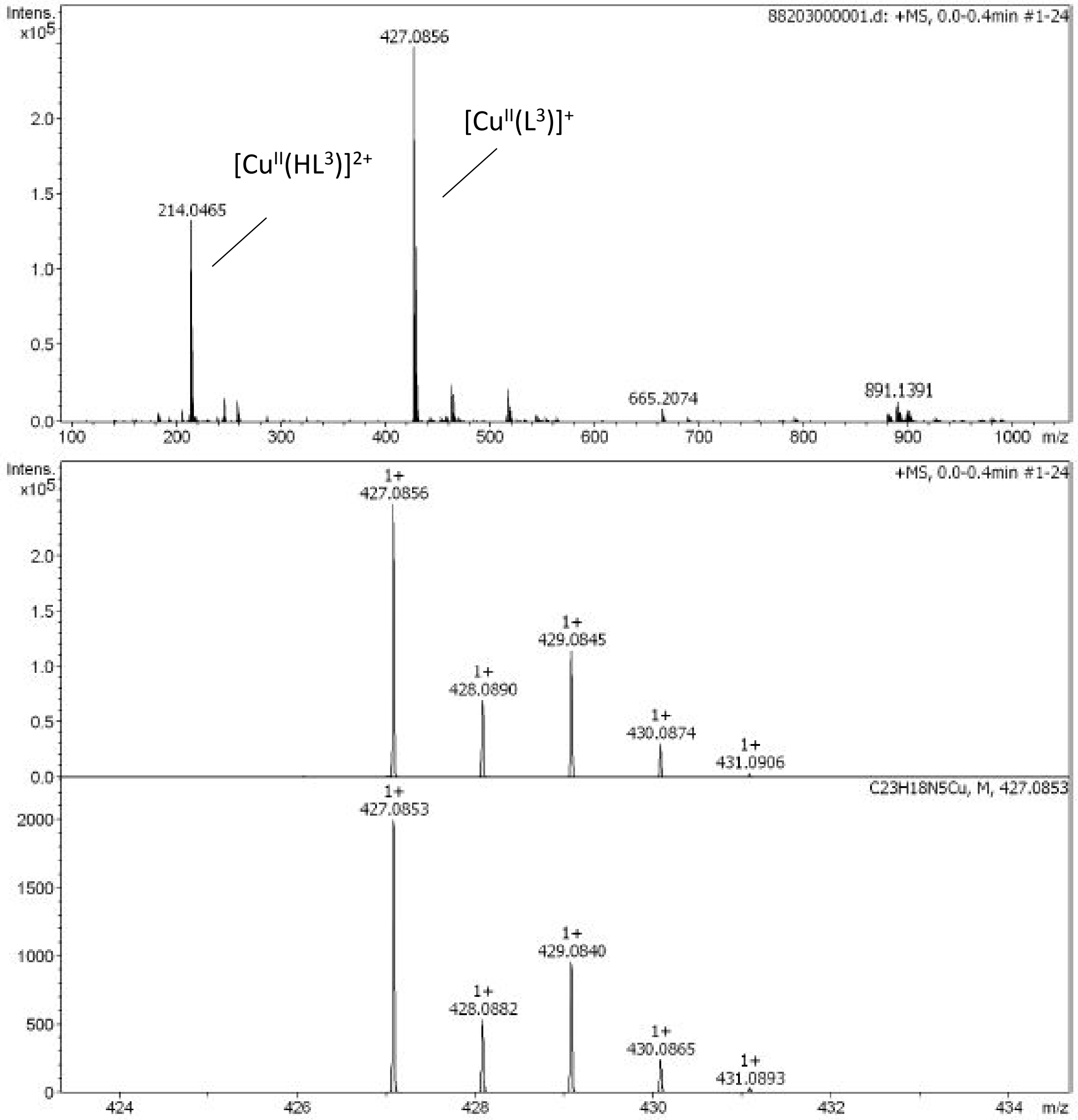

Bruker Compass DataAnalysis 4.1 printed: 4/14/2022 12:26:57 PM by: msc Page 1 of 1

Figure S26. ESI mass spectrum of **3.**

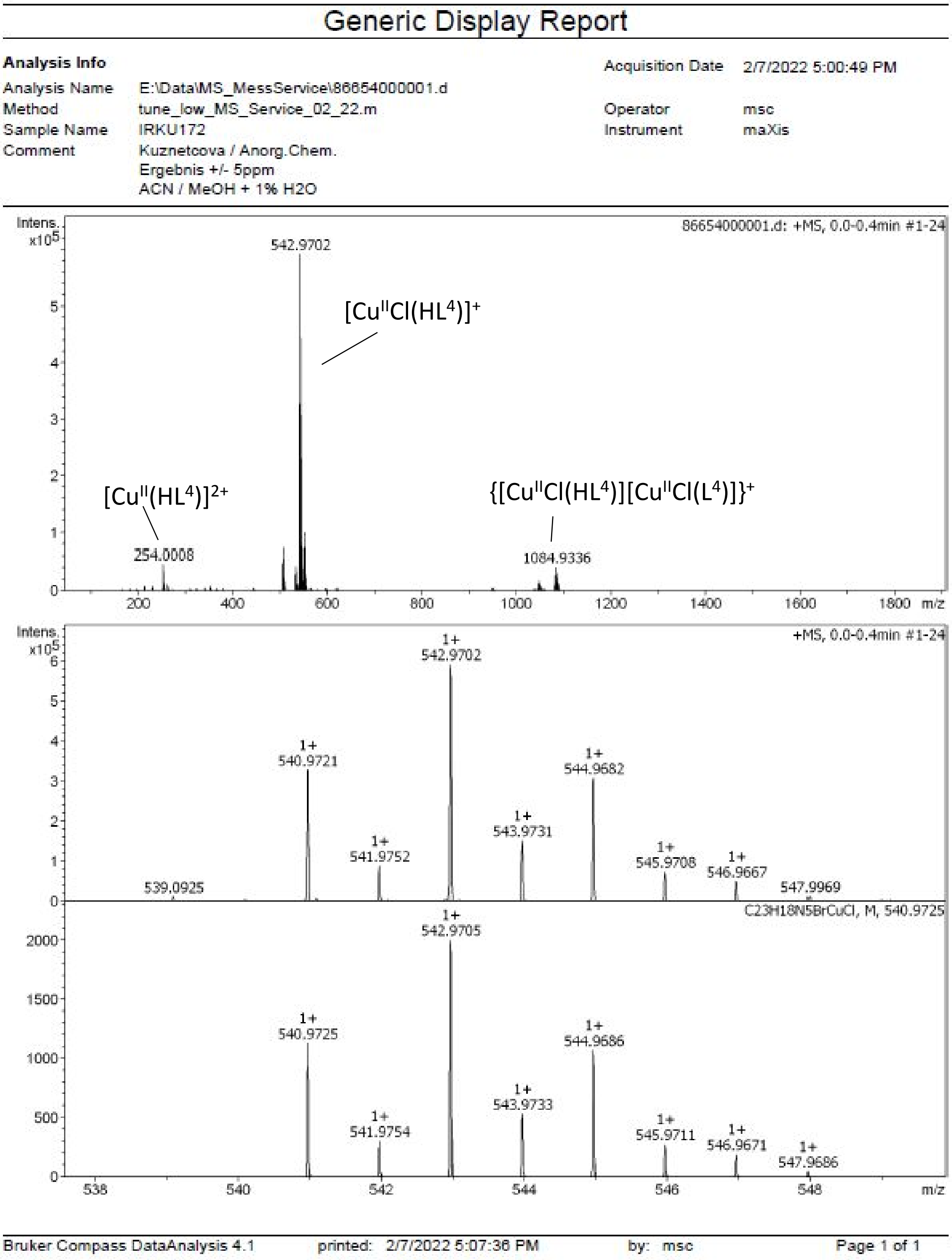

Figure S27. High resolution ESI mass spectrum of **4.**

X-ray crystallography

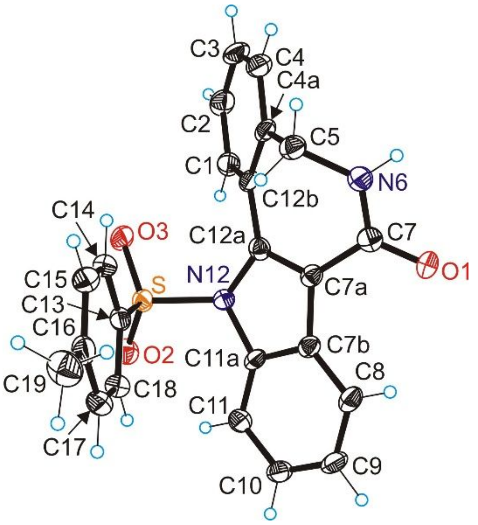

Figure S28. ORTEP view of intermediate species **f** with thermal ellipsoids at 50% probability level. Selected bond distances (Å) and torsion angles (deg): C5–N6 = 1.466(3), N6–C7 = 1.329(3), C7–O1 = 1.252(2), C7–C7a = 1.476(3); $\Theta_{C7a–C12a–C12b–C4a}$ = –37.3(3)°.

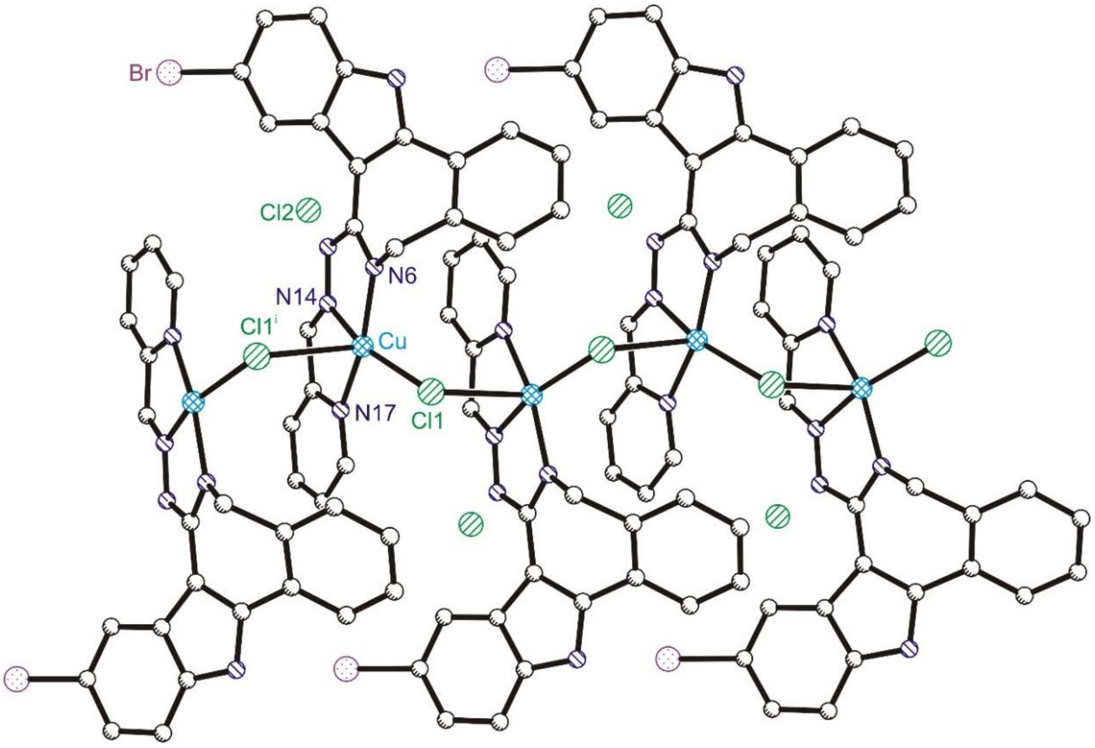

Figure S29. A fragment of the crystal structure of **2** showing the formation of 1D polymeric chain. The atom labeled with (i) was generated via symmetry transformation *x*, –*y*+1, 0.5+*z*.

UV–vis spectra

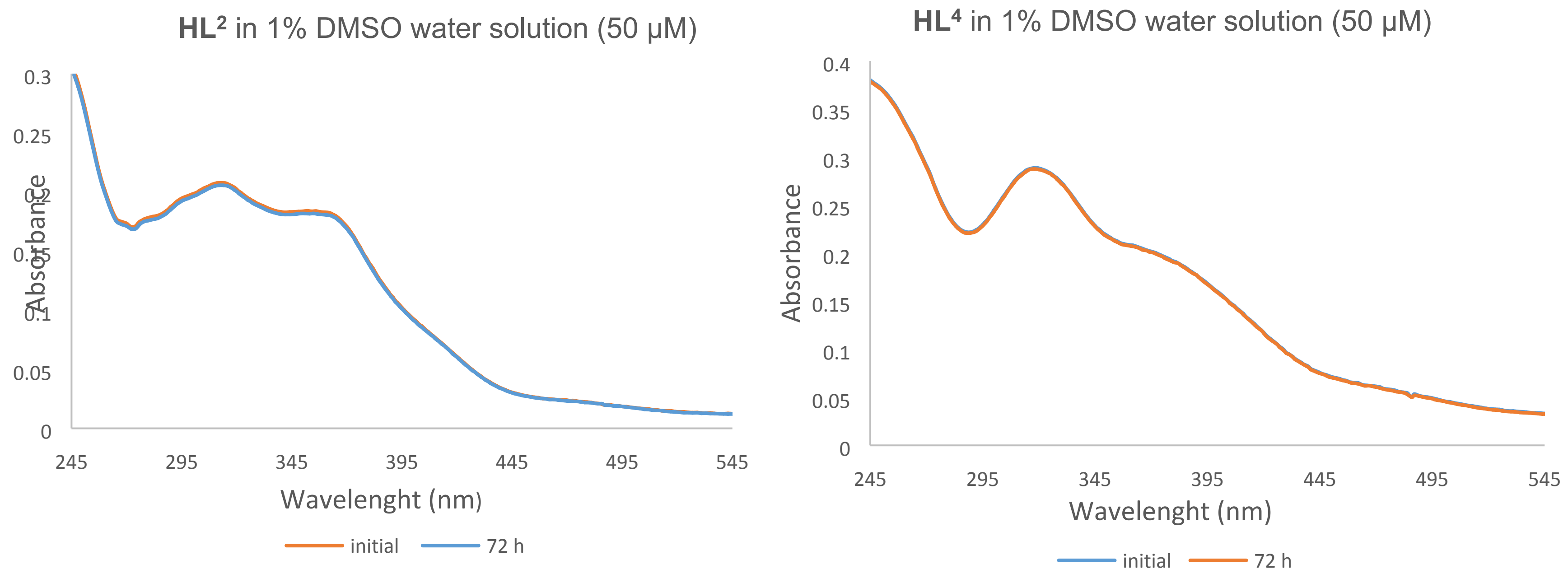

Figure S30. UV–vis spectra for ligands **HL²** (left) and **HL⁴** (right) in aqueous solution containing 1% DMSO (v/v) over 72 h.

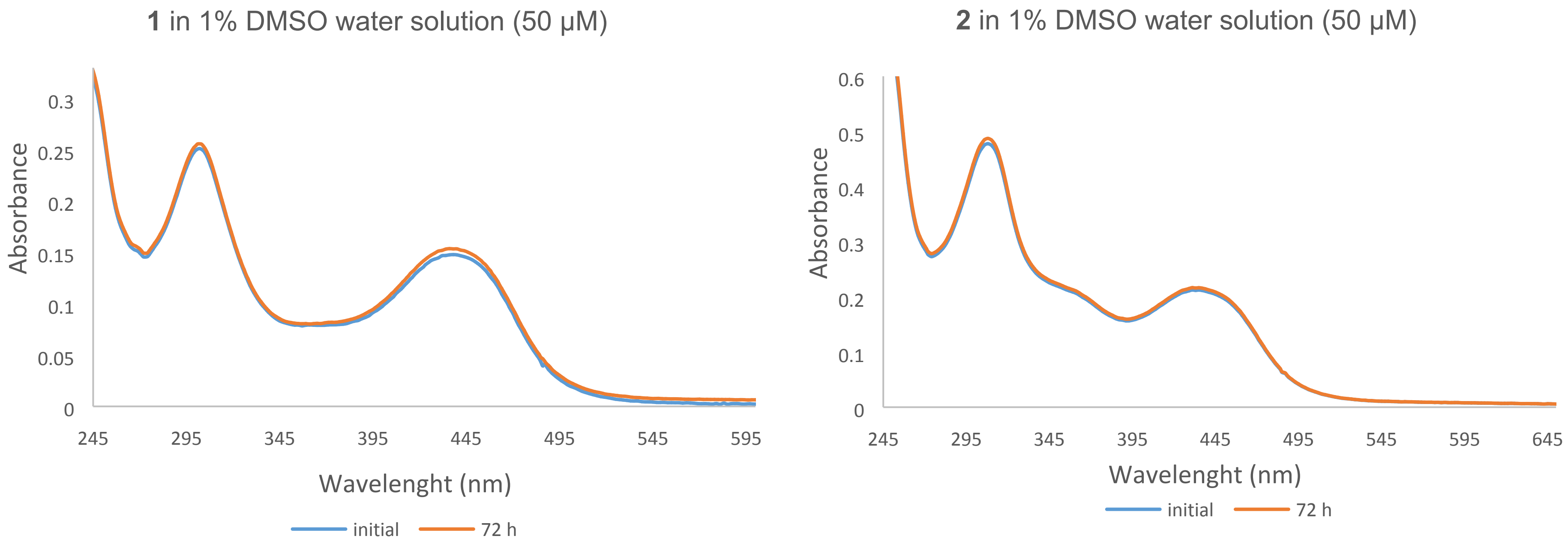

Figure S31. UV–vis spectra for complexes **1** (left) and **2** (right) in aqueous solution containing 1% DMSO (v/v) over 72 h.

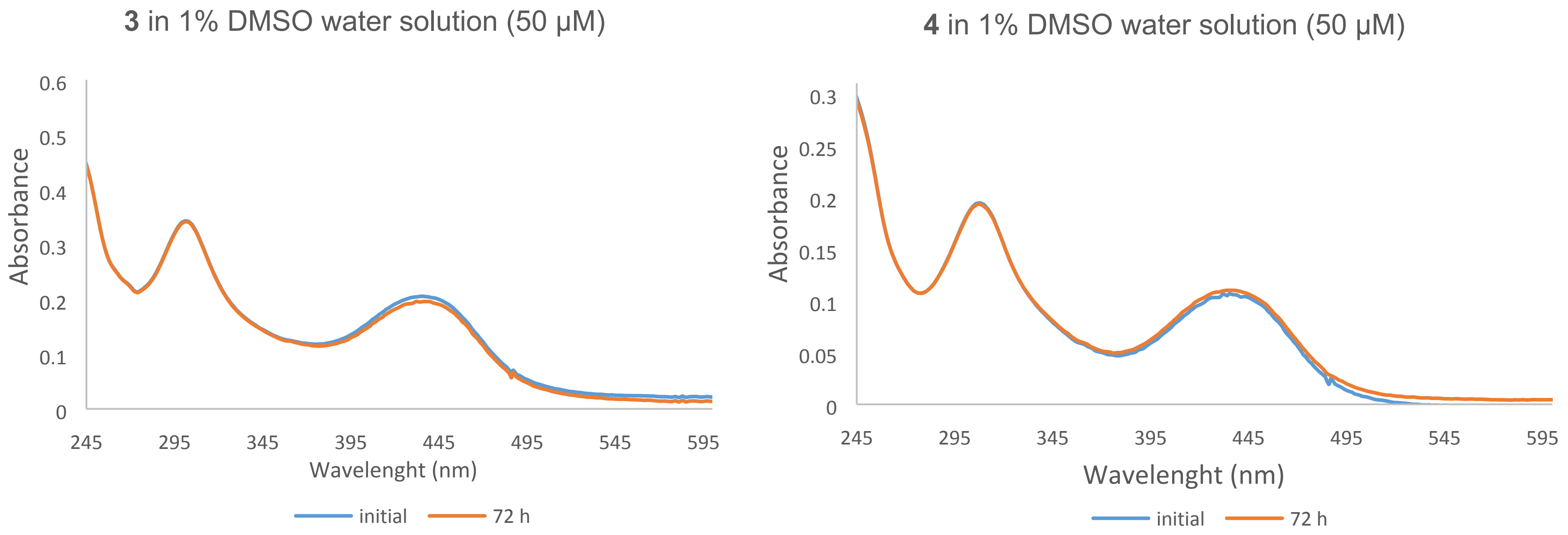

Figure S32. UV–vis spectra for complexes **3** (left) and **4** (right) in aqueous solution containing 1% DMSO (v/v) measured over 72 h.

Table S1. The molecular descriptors based on the Lipinski´s rules[a] as calculated by SwissADME, as well as experimental solubility and pH-values.

Compound	MW, (g/mol)	HD	HA	Log P^b	Log$S$ (S, mol/L) ESOL[c]
**HL[1]**	351.40	2	3	3.58	–4.82 (1.52 × 10^{-5})
**HL[2]**	430.30	2	3	4.23	–5.72 (1.90 × 10^{-6})
**HL[3]**	365.43	2	3	3.85	–5.02 (9.47 × 10^{-6})
**HL[4]**	444.33	2	3	4.41	–5-93 (1.18 × 10^{-6})
**1**	449.39	1	1	2.62	–6.00 (9.92 × 10^{-9})
**2**	528.29	1	1	3.16	–6.91 (1.23 × 10^{-7})
**3**	463.42	1	1	2.90	–6.27 (5.40 × 10^{-7})
**4**	542.32	2	3	4.23	–5.27 (1.90 × 10^{-6})

[a]Lipinski: MW ≤ 500 g/mol; Log$P$ ≤ 5; H-bond donors ≤ 5; H-bond acceptors ≤ 10; [b]LogP = partition coefficient between octanol and water;; [c]Solubility class (Log$S$ scale): insoluble < –10 < poorly < –6 < moderately < –4 < soluble < –2 very < 0 < highly.

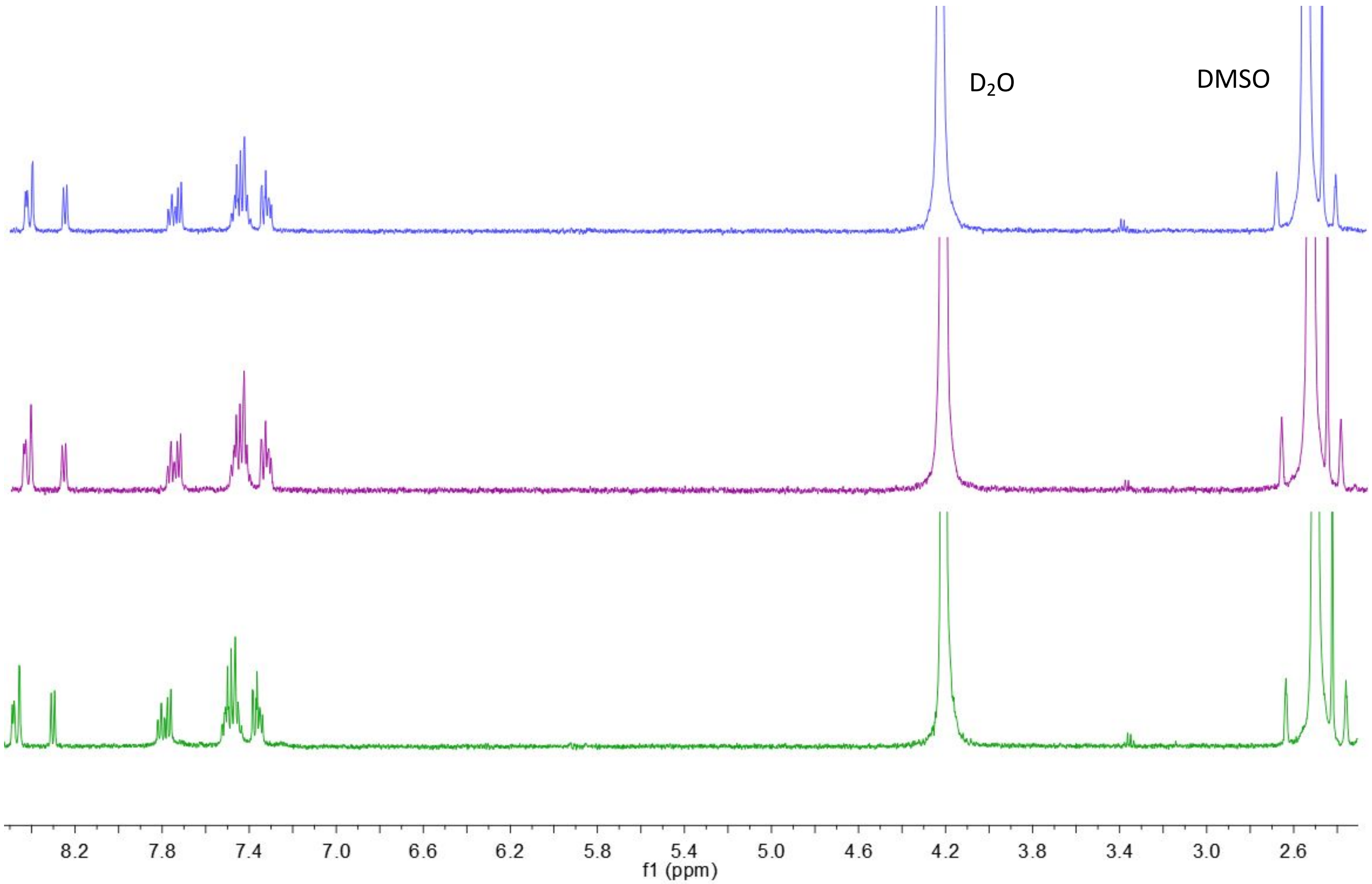

Figure S33. ^{1}H NMR spectra of **HL^{4}** in DMSO-*d*$_6$/D_2O 1:1 measured vs time. From top to bottom: blue: t = 0 min; lilac: t = 1 h; green: t = 24 h.

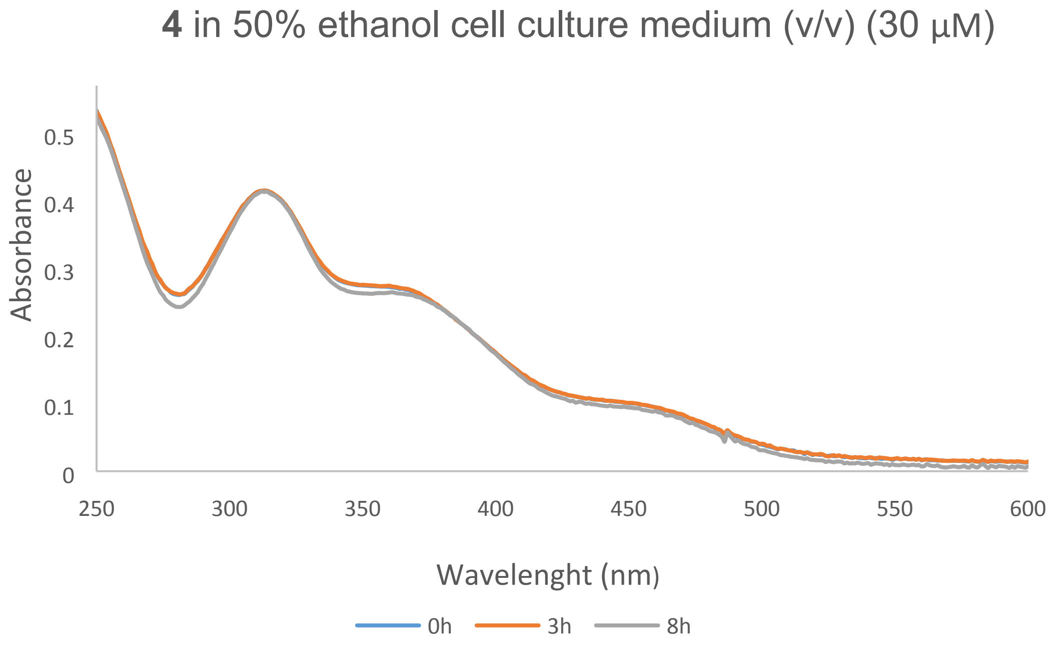

Figure S34. UV–vis spectra for complex **4** in ethanol-cell culture medium 1:1 (v/v) measured over 8 h.

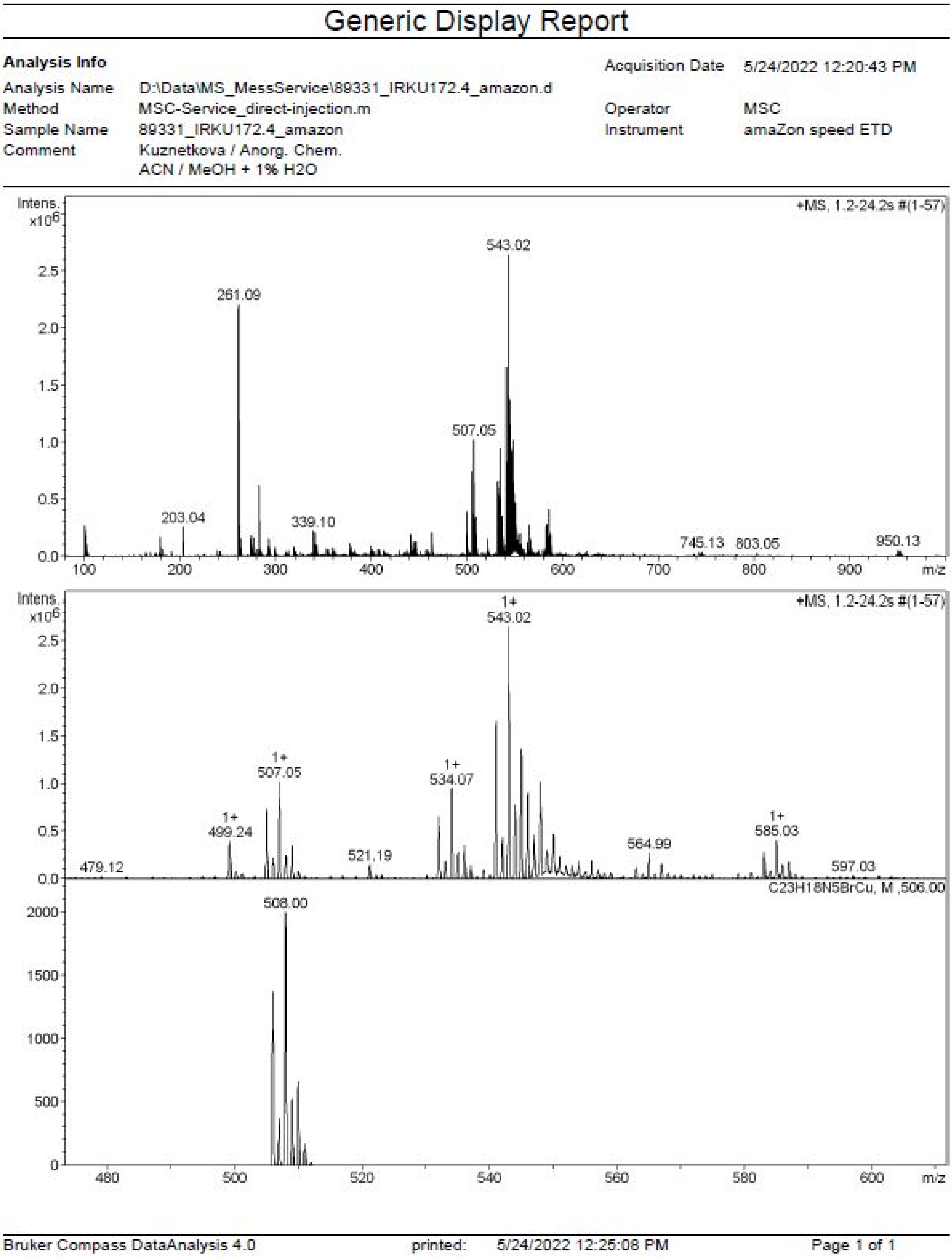

Figure S35. ESI mass spectrum of **4** in ethanol-cell culture medium (v:v) after 8 h incubation.

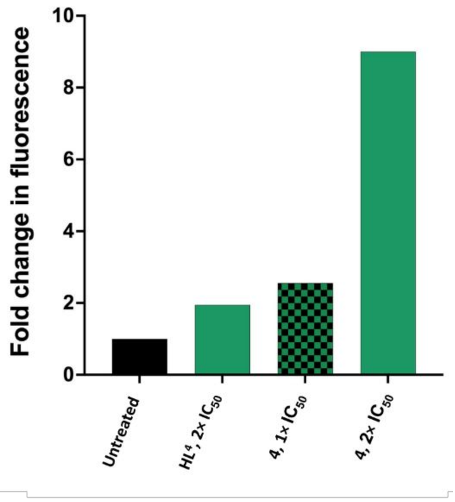

Figure S36. Quantification of ROS generation using fluorescence microscopy (results in Figure 4). Different concentrations of compound **4** (0.25 μM and 0.5 μM) and **HL⁴** (0.4 μM) were used.
